# CyclinPred: A SVM-Based Method for Predicting Cyclin Protein Sequences

**DOI:** 10.1371/journal.pone.0002605

**Published:** 2008-07-02

**Authors:** Mridul K. Kalita, Umesh K. Nandal, Ansuman Pattnaik, Anandhan Sivalingam, Gowthaman Ramasamy, Manish Kumar, Gajendra P. S. Raghava, Dinesh Gupta

**Affiliations:** 1 Structural and Computational Biology Group, International Centre for Genetic Engineering and Biotechnology (ICGEB), Aruna Asaf Ali Marg, New Delhi, India; 2 Bioinformatics Centre, Institute of Microbial Technology, Chandigarh, India; University of California, Berkeley, United States of America

## Abstract

Functional annotation of protein sequences with low similarity to well characterized protein sequences is a major challenge of computational biology in the post genomic era. The cyclin protein family is once such important family of proteins which consists of sequences with low sequence similarity making discovery of novel cyclins and establishing orthologous relationships amongst the cyclins, a difficult task. The currently identified cyclin motifs and cyclin associated domains do not represent all of the identified and characterized cyclin sequences. We describe a Support Vector Machine (SVM) based classifier, CyclinPred, which can predict cyclin sequences with high efficiency. The SVM classifier was trained with features of selected cyclin and non cyclin protein sequences. The training features of the protein sequences include amino acid composition, dipeptide composition, secondary structure composition and PSI-BLAST generated Position Specific Scoring Matrix (PSSM) profiles. Results obtained from Leave-One-Out cross validation or jackknife test, self consistency and holdout tests prove that the SVM classifier trained with features of PSSM profile was more accurate than the classifiers based on either of the other features alone or hybrids of these features. A cyclin prediction server- CyclinPred has been setup based on SVM model trained with PSSM profiles. CyclinPred prediction results prove that the method may be used as a cyclin prediction tool, complementing conventional cyclin prediction methods.

## Introduction

Cyclins were first identified in early 1980s in the eggs of marine invertebrates [1], [2]. Subsequently, cyclins have been discovered in many organisms [3], [4]. Cyclins bind and activate members of the Cdk protein family to regulate the cell cycle. The periodicity of cyclin concentrations during the cell cycle leads to periodic oscillations in Cdk activity that governs the cell cycle control system. Different cyclin-Cdk complexes are activated at different points during the cell cycle [5]–[8]. Cyclins have been classified into four general classes based on function and timing of activity namely, G1, G1/S, S and M cyclins. In most species, diverse multiple forms have been discovered, hence the cyclins were further classified on the basis of amino acid sequence comparisons, such as G1:C, D, E and G2:A, B cyclins and several other classes [9]. Cyclin homologues have also been found in various viruses; for example *Herpesvirus saimiri* and Kaposi's sarcoma-associated herpesvirus [10].

Cyclin family proteins are 30 to 65 kDa in size, sharing considerable sequence heterogeneity but share some common structural motifs known as ‘cyclin fold’. However, the conserved ‘cyclin fold’ is also found in general transcription factors (for example TFIIB) and tumor suppressor retinoblastoma protein [11]. Multiple alignments of the amino acid sequences of cyclins have identified a region of similarity, extending around 100 amino acids, termed as cyclin box [12], [13]. The conserved region has been termed as a cyclin related domain using Hidden Markov models by Pfam database [14] and a cyclin motif in PROSITE database [15]. However, the cyclin signatures are completely absent in several annotated cyclin sequences. Some members of G2/mitotic-specific and cyclin T family cyclins neither possess PROSITE motif nor Pfam cyclin domains, as discussed later in the dataset section. New cyclin genes and proteins have been characterized using various strategies for example using BLAST to identify cyclins in *Caenorhabditis elegans*, *Drosophila melanogastor* and *Homo sapiens* [16], and Hidden Markov Models (HMM) based methods in *Arabidopsis* genome [17]. In general, simple BLAST or advanced PSI-BLAST (Position Specific Iterated BLAST, [18]) is used as a first choice for functional annotation of predicted proteins or proteins of unknown function. The choice of similarity based approaches is reasonable only if query proteins are similar to database sequences or profile generated from them. This limitation is easily overcome by machine learning techniques like Support Vector Machine (SVM).

SVM is a supervised learning algorithm, which has been found to be useful in recognition and discrimination of hidden patterns in complex datasets [19]. Prediction methods based on SVM have been successfully exploited in many research problems involving complex, sequence or biological datasets [20]–[22], like remote protein similarity detection [23], DNA methylation status [24], protein domains [25] and multiclass cancer diagnosis [26]. SVMs learn from a training data sample consisting of both positive as well as negative examples of a classification problem along with their features. The SVMs numerically finds the distinguishing features of a particular class which may be used for classification. In short, SVMs initially map input data (in terms of negative and positive class input vectors) into a high dimensional feature space using a kernel function [27]–[29]. The input or feature vectors in the feature space are then classified linearly by a numerically optimized hyper plane, separating the two classes [30], [31]. The hyper plane depends only on a subset of training examples, called Support Vectors (SVs).

In context to the current study, SVMs learn the features specific to the cyclin sequences and generate support vectors decisive for possible classification of any given sequence as cyclin. For the cyclin classification problem, a feature vector (x_i_) could represent the Amino Acid Compositions (AAC), Di-Peptide Compositions (DPC) of a protein sequence, Secondary Structure Composition (SSC) or any other training feature. AAC gives a fixed input pattern length of 20 whereas DPC gives a pattern of length 400 [32]. The fixed length pattern of training features is a basic requirement of SVM training input. DPC features are better than AAC features since it provides both the fractional composition of each amino acid as well as their local order in the protein sequence. Both the composition features were used as training inputs to classify cyclin sequences from non-cyclin sequences using SVM. Apart from the composition features, we also used the feature vectors of the secondary structure information and PSSM profile of the training sequences, obtained from PSIPRED (Protein Structure Prediction Server [33]) and PSI-BLAST analysis respectively [18]. We used SVM*^light^* package [34], [35] for implementation of the SVM training for classification of sequences.

The optimized classifier was trained with different properties of a non redundant dataset consisting of 68 cyclin (positive dataset) and 72 non cyclin (negative dataset) sequences (for details, see the supporting information files). Different properties of sequences like amino acid composition and dipeptide composition, and PSI-BLAST results were used for training and optimization of the classifiers. Optimization of different classifiers was performed using Leave-One-Out (LOO) cross validation (CV) technique or jackknife test, Self-consistency and Holdout tests. The biasness of selected classifiers was checked by calculating prediction efficiency for an independent dataset sequences not used in the training as well as annotated sequences of *Arabidopsis thaliana*. Pfam and PROSITE search of the training dataset cyclin sequences revealed that cyclin associated domains and motifs are not present in all the cyclin sequences. Principal Component Analysis (PCA) was also performed for the training variables to find out the variables important for distinguishing cyclins from non cyclin sequences.

## Results

### Performance of similarity based search (PSI-BLAST)

Three iterations of PSI-BLAST were carried out at E value 0.001. The performance of PSI-BLAST was evaluated using jackknife cross validation method (identical to jackknife CV test used for the evaluation of SVM models), where each sequence in the training dataset was used as a BLAST query sequence and remaining sequences were used as BLAST database. Thus, in the process of jackknife test, each protein sequence is used as a test sequence, and for other rounds; the sequence is included in the training dataset. In PSI-BLAST output, it was observed that out of 68 cyclin proteins, no significant hits were obtained for 8 proteins, thereby resulting in overall accuracy of 88.2%. This implies that similarity-based search methods alone may not be the best choice for functional annotation of proteins. Therefore, we decided to explore methods based on other protein features for the prediction of cyclin proteins.

### Preference of amino acids in cyclins and non-cyclins

**Figure 1:**
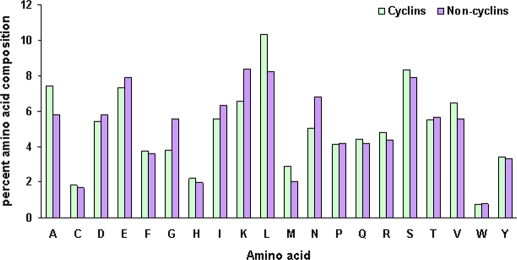
Amino Acid Composition. Frequency of each of the amino acid in cyclin and non-cyclin protein sequences used in the training dataset. The plot reveals the differential amino acid propensities in cyclins and non-cyclin sequences, especially with respect to that of Leu, Gly, Ala, Val, Lys and Asn.

AAC (Amino Acid Composition) analyses of cyclin and non-cyclin protein training sequences (Figure 1) reveal differential amino acid propensities in cyclins and non-cyclin sequences, especially with respect to that of Leu, Gly, Ala, Val, Lys and Asn. The percentage composition of Leu, Ala and Val is higher in cyclin protein sequences whereas the percentage composition of Gly, Lys and Asn is higher in non-cyclin sequences used in the training. AACs of other amino acids were not significantly different in cyclin and non-cyclin protein sequences. It was expected from the observations that the AAC information alone was not enough to classify the cyclins. Hence, we used other sequence features like DPC (Di-Peptide Compositions), SSC (Secondary Structure Composition) and PSSM in conjunction with AAC to train different SVMs based on different kernels.

### Performance of standalone SVM models

We performed training-testing cycles recursively to develop SVM models based on different features as well as combination of all the features (referred to as hybrids) (Table 1). Initially, we performed jackknife test (LOO CV) of AAC, DPC and PSSM by optimization with all the three kernels, namely: linear, polynomial and RBF (Radial Basis Function).

**Table 1:** Performance of SVM classifiers for various combinations of protein sequence features, kernels, parameters and validation methods.

Model	Feature	Dm	Validation	ACC (%)	SN (%)	SP (%)	MCC	F1	Parameters	
									C	γ
Module1	AAC	20	a	**83.57**	83.82	83.33	0.671	0.712	1.5	84
Module2	DPC	400	a	**85.13**	85.29	84.72	0.699	0.734	30	12
Module3	SSC	60	a	**80.71**	76.47	84.72	0.614	0.658	7	10
Module4	PSSM	400	a	**92.14**	92.64	91.66	0.842	0.851	32.5	1
			b1	**97.85**	97.05	98.61	0.957	0.889	47.5	0.5
			b2	**100**	100	100	1	1	0.5	100
			c1	**i) 88.57**	88.23	88.88	0.771	0.787	4	9.6
			c2	**ii)94.28**	91.17	97.22	0.886	0.872	19	0.5
Hybrid-1	AAC+DPC	420	a	**81.42**	82.35	80.55	0.628	0.682	880	0.1
Hybrid-2	AAC+SSC	80	a	**85.71**	89.70	81.94	0.717	0.753	10.8	10
Hybrid-3	DPC+SSC	460	a	**81.42**	79.41	83.33	0.628	0.675	10.8	10
Hybrid-4	PSSM+AAC	420	a	**91.42**	92.64	90.27	0.828	0.84	230	0.1
Hybrid-5	PSSM+DPC	800	a	**91.42**	92.64	90.27	0.828	0.84	30	1
Hybrid-6	PSSM+SSC	460	a	**92.14**	92.64	91.66	0.842	0.851	19.2	1.5
			b1	**98.57**	97.05	100	0.971	0.904	19.2	1.5
			b2	**100**	100	100	1	1	0.5	50
			c1	**i) 88.57**	88.23	88.88	0.771	0.787	0.5	30
			c2	**ii)88.28**	82.35	86.11	0.685	0.753	3	7
Hybrid-7	PSSM+DPC+SSC	860	a	**90.0**	91.17	88.88	0.80	0.815	10	2
Hybrid-8	PSSM+DPC+AAC	820	a	**91.42**	92.64	90.27	0.828	0.84	200	0.1
			b1	**95.71**	97.10	94.46	0.914	0.847	200	0.1
			b2	**100**	100	100	1	1	0.5	100
			c1	**i) 85.71**	73.52	97.23	0.731	0.862	0.5	19
			c2	**ii)91.42**	97.05	86.11	0.834	0.774	0.3	4

Dm: dimension, a = Jackknife test CV, b1 = self-consistency test (mode 1), b2 = self-consistency test (mode 2), c1 & c2 = holdout-test, SN: sensitivity, SP: specificity, MCC: Mathew's Correlation Coefficient, F1: F1 statistics, C: tradeoff value, γ: gamma factor (a parameter in RBF kernel).

#### Composition based models

We found that for the SVM models based on AAC features, the linear and polynomial kernels yielded similar accuracies (83.28% and 83.57%, respectively). Optimizing the parameters with RBF kernel yielded an accuracy of 83.57% which is comparable to that of the other two kernels (Table 1). Hence, AAC based SVMs trained with different kernels have similar accuracies. To check whether these accuracies are due to artifact of the training dataset, we generated another sets of non-cyclin sequences belonging to few non-cyclin families (for example regulatory proteins and membrane bound proteins etc.) and used them as non-cyclin dataset for training AAC based SVM classifier: we found that each classifier had comparable specificity and sensitivity values (results not shown here). For DPC feature based model, the training classifiers based on linear and polynomial kernels yielded accuracies of 83.74% and 81.43% respectively. However, the RBF kernel yielded a higher accuracy of 85.13% (for γ = 12, C = 30). Accuracy of SSC based SVM classifier was 80.71%, which is less as compared to that of the AAC and DPC based SVM classifiers.

#### PSSM profile based classifier model

**Figure 2:**
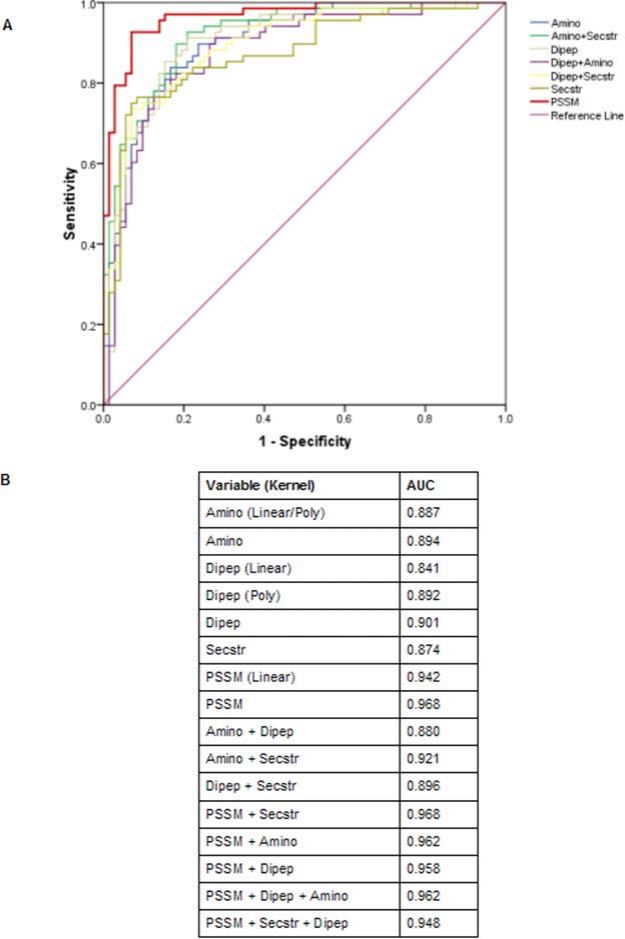
Threshold-independent performance of SVMs. (A) ROC plot of SVMs based on different protein sequence features which depicts relative trade-offs between true positive and false positives. The diagonal line (line of no-discrimination) represents a completely random guess. Closer a point in the upper left corner of the ROC space, better is the prediction as it represents 100% sensitivity (when all true positives are found) and 100% specificity (when no false positives are found). The PSSM based (standalone as well as hybrids) SVM models show a similar prediction having AUC more than 95%. (B) Area under curve (AUC) obtained from the ROC plot. All SVM models are based on RBF kernel unless mentioned.

The optimization with linear and polynomial kernels for PSSM profile based classifier model resulted in ∼91% accuracy for both the kernels. However, SVM optimized with RBF kernel augmented the accuracy to 92.14% (for γ = 1 and C = 32.5), which was the best performance amongst the classifiers based on individual features. It had a sensitivity of 92.64% and specificity of 91.66% with MCC of 0.842. The comparison of results obtained with all the four features; clearly demonstrate that the best classification efficiency was achieved by the PSSM based model which incorporates the information about residue composition, position specific substitutions and evolutionarily conserved residues in the protein sequences. Also, the efficiency of RBF kernel based discrimination between cyclins and non-cyclins as compared to other kernels for AAC, DPC and PSSM and ROC (Receiver Operating Characteristic) plot study (Figure 2B) suggested that RBF kernel is more efficient and appropriate for the cyclin training dataset and hence we used the RBF kernel for evaluation of all the SVM classifiers.

### Performance of hybrid-SVM classifier models

In our efforts to further improve the prediction accuracy, we developed and evaluated eight hybrid SVM classifier models (hybrid-1 to hybrid-8), results of which are summarized in the Table 1.

#### Hybrid models based on two features

The first hybrid-SVM model (hybrid-1) was developed using AAC and DPC features of the training dataset sequences. The prediction accuracy of hybrid-1 model was 81.42% with sensitivity of 82.35% and specificity of 80.55%, which was not better than that of standalone AAC and DPC based SVM models. The hybrids-2 and 3 were based on secondary structure predictions, amino acid and dipeptide compositions. We found that hybrid-2 has greater accuracy as compared to that of hybrid-3 (85.71% and 81.42%, respectively); also better than the accuracy of individual SSC or AAC based SVM models however, comparable to the individual DPC based model. Further, when the training was done using features from both AAC and PSSM (hybrid-4) or DPC and PSSM (hybrid-5), the accuracy increased to 91.42%, as compared to hybrids-1 to 3.

However, amongst the hybrid-classifier models based on two features (hybrid:1–6), the best accuracy of 92.14% was achieved by combining the SSC and PSSM pattern information in hybrid-6 classifier, with a sensitivity of 92.64% and specificity of 91.66%. This SVM model has a MCC value of 0.842, highest amongst the hybrid-SVM classifier models.

#### Hybrid models based on more than two features

Considering the importance of conserved secondary structures in cyclins, we decided to use SSC in conjunction with the training features of the best hybrid-classifier developed till this stage other than hybrid-6 (i.e. hybrid-5 based on PSSM and DPC features). Hence, we developed another hybrid model using the information from PSSM, DPC and SSC features (hybrid-7), with a pattern length of 860 features. Unexpectedly, the accuracy of this hybrid model was 90.01% (γ = 2 and C = 10), lower than the accuracies of hybrid-5 (PSSM+DPC = 91.42%) and hybrid-6 (PSSM+SSC = 92.14%) classifier models. Another hybrid model of PSSM, DPC and AAC (hybrid-8) was developed with which we were able to achieve an accuracy of 91.42%, similar to that of hybrid-4 and 5. Thus, the accuracy achieved by hybrid-6 and PSSM based model were the best amongst all the hybrids and standalone models, further supported by the highest MCC of these models.

### Self-consistency and holdout validation tests

We carried out self-consistency tests of the best models namely: PSSM, hybrid-6 and hybrid-8 to evaluate their learning capability. We used two different modes to carry out the self-consistency test (as described in the methods). Mode-2 self-consistency test gave an accuracy of 100% and a MCC of 1 (with different γ values of RBF kernel) whereas mode-1 showed different accuracies for respective models (Table 1). It is interesting to note from mode 1 that PSSM based model and hybrid-6 have comparable performances (∼98% accuracy) and better than hybrid-8 (∼95% accuracy). A high accuracy and MCC from self-consistency method implies that SVM has inherited the intrinsic correlation between feature vectors and the classification searched for.

Further, the performance of PSSM based model and hybrid-6 (PSSM+SSC) were assessed by holdout tests. The holdout test results are highly convincing in terms of accuracies and MCCs (∼0.8 for PSSM model and ∼0.7 for hybrid-6). The tests results have ruled out any skewness, biasness or variance in the results due to random splitting of the dataset. Also, as holdout tests simulate the random/blind test performance for a large dataset, the results obtained here reflect a strong discriminative power of the classifier.

### Comparison of classifier's performance with random prediction and F1 statistics

Upon comparing the prediction reliability with that of random prediction (Table 2), it was clearly observed that normalized prediction accuracy S, in models trained with features like AAC (67.12%) or DPC (69.98%) or even SSC (61.31%), were not better than that of hybrids (>80% except hybrids 1–3) or highly specialized feature like PSSM (84.27%). The two best models obtained from this comparative study were PSSM based model and hybrid model (hybrid-6, S = 84.27%). Again, F1 statistics of both the models were similar (0.851) and better than that of others. It suggests that precision and recall capacity of the models are good enough to classify the protein classes.

**Table 2:** Estimation of quality for best SVM model for each feature or combinations of features (hybrid models) as compared to random prediction (S).

Model	Feature	Correct (TP+TN)	S (%)
Module1	AAC	117	67.12
Module2	DPC	119	69.98
Module3	SSC	113	61.31
Module4	PSSM	129	84.27
Hybrid-1	AAC+DPC	114	62.85
Hybrid-2	AAC+SSC	120	71.47
Hybrid-3	DPC+SSC	114	62.79
Hybrid-4	PSSM+AAC	128	82.85
Hybrid-5	PSSM+DPC	128	82.85
Hybrid-6	PSSM+SSC	129	84.27
Hybrid-7	PSSM+DPC+SSC	126	80.00
Hybrid-8	PSSM+DPC+AAC	128	82.85

TP: true positive, TN: true negative.

S: percentage of random prediction.

### ROC plot

For each SVM model, threshold-independent performance was measured by plotting ROC curve between TP rate (sensitivity) and FP rate (1-specificity) values. ROC indicates the performance of all SVM models optimized with best parameters. Figure 2A shows the details of AUC (Area Under Curve). The AUC for hybrid-6 and PSSM model is 96.8% which is again the highest amongst all AUCs of the other models. From the Figure 2B, area under curve of simpler feature like AAC based SVM model, standardized with RBF kernel is 89.4%, similar to linear and polynomial kernels whereas more advanced features like DPC and PSSM has a better AUC when optimized with RBF kernel as compared to other two kernels. For example, AUC of DPC based model was 90% (RBF) as compared to 84% (linear) and 89% (polynomial). This clearly demonstrates that RBF kernel has much better ability in distinguishing cyclin from non-cyclin sequences.

### Performance on independent dataset

To evaluate the unbiased performance of the PSSM based and hybrid-6 based SVM classifiers, an independent / blind-test evaluation was carried out on the test set of 54 PROSITE false-negatives (described in the methods). The prediction accuracy of the PSSM classifier was found to be 98.15% as compared to that of hybrid-6 (92.58%). The available cyclin HMM profile (Pfam database) predicts cyclin domain for 50 sequences of this test set; therefore it failed to predict 4 sequences. The PSSM based SVM model was able to predict 3 out of 4 sequences, apart from correctly predicting all other 50; making a total of 53 correct predictions out of 54. However, the hybrid-6 classifier was able to predict 50 sequences, similar to Pfam HMM profile prediction. Therefore, the blind-test performance clearly established that the PSSM based SVM classifier has better prediction capacity with minimum error as false positives/negatives. Also, using the PSSM based SVM classifier, we were also able to predict the 30 newly identified cyclins in *A. thaliana* genome that were predicted earlier by a HMM based method developed by Vandepoele et al. [17]. In addition to these predicted proteins, with high SVM score cutoffs - 34 additional sequences were predicted as cyclins, for which no annotations are available so far. It is important to mention here that 25% of the predicted proteins in the *A. thaliana* genome, 8073 protein sequences, are yet poorly annotated and mostly described as ‘unknowns’. However, an elaborative analysis and supporting evidences may be needed from case to case before any significant remarks can be made regarding these predicted cyclins.

### Feature analysis using PCA

**Figure 3:**
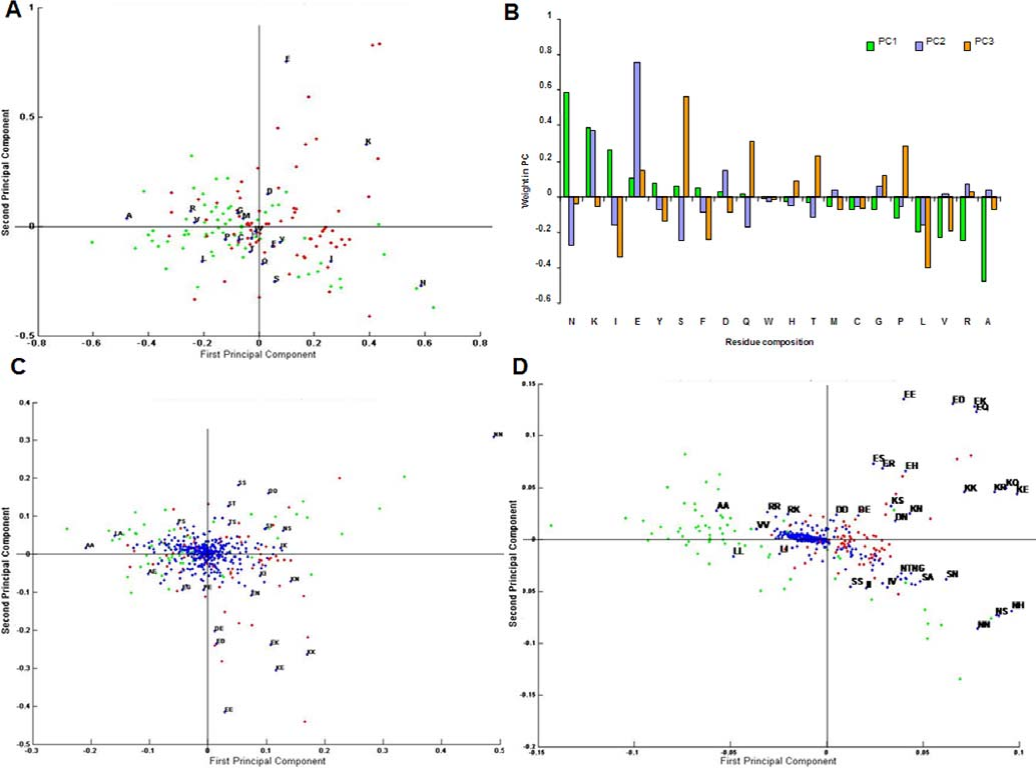
Component loadings for the first two Principal Components (PC). (A, C, D) Superimposed plot of component loadings of features used (AAC, DPC and PSSM) and training dataset from PCA analysis - showing the feature usage variability, thereby showing what degree the original variables contribute to the PCs. The plot signifies the correlations between amino acids by virtue of its loading scores as well as relative abundance in cyclins and non-cyclins to each of the PC analyzed. Green, red and blue spots represent cyclin, non-cyclins and component loadings of feature used, respectively. (B) PC weight plot of each of the 20 amino acids for the first three PCs of AAC model. The plot signifies the discriminative properties of amino acids to specific PCs by virtue of its loading scores.

PCA was carried out to identify important elements from AAC, DPC and PSSM features that are capable of distinguishing cyclin from non-cyclin sequences. The PCA plots between PC1 and PC2 for each sequence feature was studied (Figure 3). From the figure, it is evident that the clustering of positive and negative examples of the training set and their mutual segregation in two dimensional space gradually increased as we moved from the model based on AAC (simplest of all features used) to a more advanced PSSM feature based model. A brief description of each of the feature is described below.

#### Amino acid composition

The component loadings plot for first two principal components (PCs) describing amino acid frequencies (Figure 3A) revealed the following facts:- PC1 explains 28.1% of the total variance of the dataset; however, first three PCs give a cumulative variability of 59%, exceeding to 75.7% for first 6 PCs. The strongest contribution to PC1 is by Asn, Ala and Arg amino acids as AAC. Asn (loading 0.586) and Lys shows positive correlations, whereas Ala (loading −0.477) and Arg shows negative correlations to the component. Similarly, PC2 (19.16% explained variance) correlates with Glu (loading 0.754) and is negatively related to Ser residue contribution (loading −0.253). Interestingly, Lys contributes almost equally to first two PCs (loadings as 0.389 and 0.374 for PC1 and PC2 respectively). It was observed that Ser contributes maximally to PC3 (11.6% explained variance) with component loading value of 0.56 and is negatively related to Leu (loading −0.401). The findings were closely correlating with the PC weight plot for AAC (Figure 3B). Similar results were observed when PCA was performed for each of the randomly splitted equal halves of the dataset; consisting of approximately same number of cyclin and non-cyclin sequences of AAC training dataset (identical to the holdout evaluation method in SVM) (data not shown here).

These findings are in agreement with the observed prevalence of amino acids in cyclins and non-cyclins (Figure 1) which indicates Ala and Leu in cyclins and Asn and Lys in non-cyclins give major contributions to first three principal components. This analysis offers some hints about distinguishing determinants in AAC model. However, the importance of Gly residue needs a closer look as it was not very prevalent distinguishing element in PC loadings plot though its sequence composition is substantially different between cyclin and non-cyclin sequences.

#### Dipeptide composition

Component loadings for the first three principal components explain only 21% of the variability of the dataset (Figure 3C). The high amino acid composition of Asn is clearly reflected in dipeptide composition too and it is principally contributing to PC1 (loading −0.489). In fact, most of the dipeptide compositions were following the observed trend of prevalence of amino acid composition. For example, dipeptides of Ala with Ala, Leu, Arg, and Ser have higher weightage towards PC1, correlating with that of the cyclin sequences. However, Ser-Ser, Ser-Ala, Ser-Asn dipeptides and their vice-versa were predominantly correlating with PC2 and non-cyclin proteins in majority. Although, a very strong correlation of training dataset examples and its dipeptides seems to be present, but as compared to PCA of AAC, a better partitioning of feature (dipeptides) among positive and negative examples of the training dataset was observed.

#### PSSM profile

The principal component loadings of PSSM profile reveal that PC1 is capable of gathering 27.7% of total variance of the dataset (Figure 3D). In totality, first three components grasped upto 58% of the variance with individual variances of 18% and 12% for PC2 and PC3 respectively. Conservative substitution of amino acids is a general phenomenon in protein sequences and was also observed in the scatter plot like Ala by Ala, Lys by Lys, Arg by Lys or Glu by Asp. The Ser and Asn substitutions were observed to be clustered more cohesively as compared to clustered distribution of Ala, Arg, Ile in cyclins and Lys and Glu in non-cyclins. Some of the outliers in the plot were observed to be unusual substitutions such as Asn by His, Asn by Thr and Lys by Gln, Glu by Ser which might be due to dominance of certain amino acids specific to the certain cyclin sub-classes under study.

### Implementation of best classifier as CyclinPred web server

**Figure 4:**
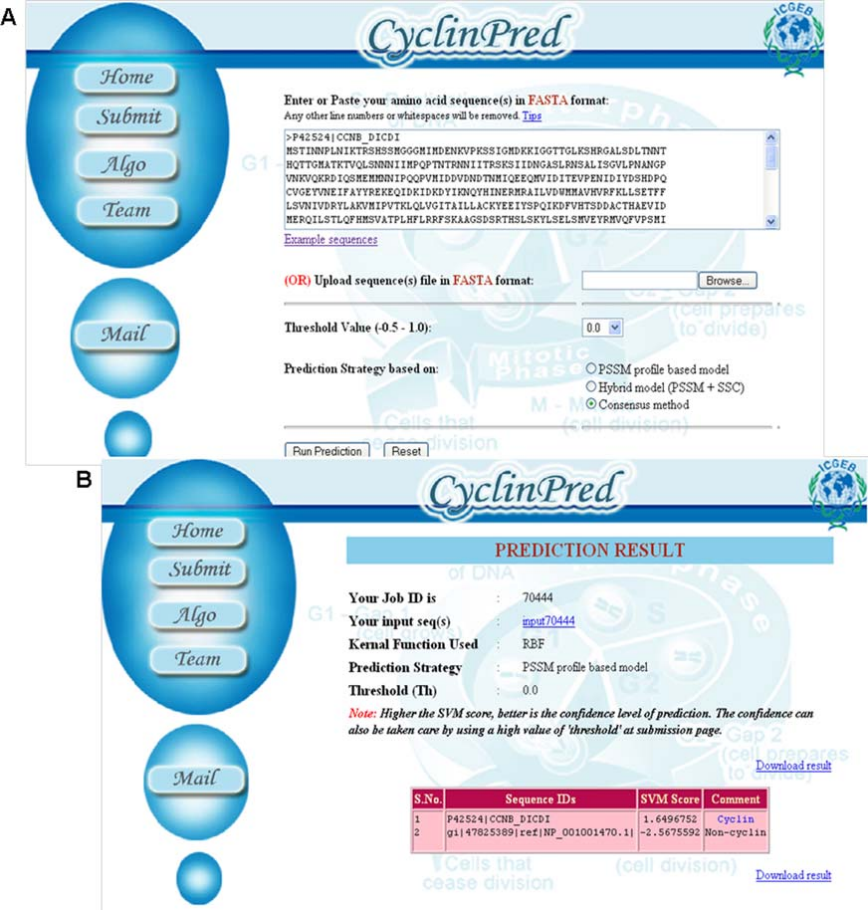
CyclinPred server. (A) Snapshot of CyclinPred server (B) Sample prediction result.

The best classifiers developed in the current study are publicly available at “http://bioinfo.icgeb.res.in/cyclinpred” The home page of the webserver is simple and designed using HTML and PHP (Figure 4) which accepts a protein sequence inputs in FASTA format. One may choose either of the given prediction strategies on the server: PSSM profile based model (the best model as default model), Hybrid model (PSSM+SSC) and Consensus method (a sequence will be predicted as cyclin only if both hybrid and PSSM models predict the sequence as cyclin). To check the time and memory constraints of running predictions on the server (CyclinPred is currently hosted on a AMD 852 4 processor server), we submitted two test sequences: the shortest cyclin sequence in the training dataset and the longest amino acid sequence (a titin protein sequence) in the SwissProt (release 55.2). The server returned the results were in 0.47 and 11.8 seconds respectively for the sequences. In a similar test run, complete results for all *A. thaliana* proteins were returned in 5 minutes and 42 seconds. The results reflect that the prediction method is not too computational intensive.

## Discussion

Recently, artificial intelligence based techniques like SVM have been used in solving problems in computational biology – including extraction of complex patterns in biological sequence databases and its use in training and classification using SVMs. Although the major limitation of SVM based methods is the requirement of fixed-length input patterns, however it is completely independent of sequence similarity. This makes SVM based methods a unique tool for analyzing proteins with very low sequence similarity. Here, we have implemented a SVM kernel-based method to develop a robust method for identification of cyclins.

Initially, the fixed length pattern of 20 was used based upon amino acid composition for the classification of proteins. This feature was earlier used to develop a method for subcellular localization of proteins [36], [37]. However, this feature provides no information about the local order of amino acids in the protein. This deficit was overcome by using dipeptide composition of amino acids giving a length of 400 [38]. The performance tuning and efficiency of the models based upon these features; individually, has been evaluated using jackknife CV, self-consistency and holdout methods. An accuracy of 83.57% and 85.13% with RBF kernel was achieved by jackknife test for classifiers based on AAC and DPC features, respectively. Such values are impressively high in context of differentiating cyclins from non-cyclins. The protocol also demonstrates that it doesn't rely upon remote sequence homology for cyclin prediction. Since, the cyclin box of cyclins contains several conserved alpha helices and extended beta sheets joined by short turns [13], this secondary structure information is a valuable feature to provide global information in conjunction with amino acid and amino acid pairs compositions. However, the SVM model trained with PSIPRED SSC features was found to have an accuracy of 80.71% (Table 1), lower than other models. The precise conformation of an amino acid also depends, along with other factors, on factors such as the neighboring residues, physico-chemical nature, and local folding stress. The preference of an amino acid for each of the three secondary structures (helix, sheet and coil) can provide us more elaborative features of the structure of a protein. Such feature vectors will encompass more valuable information as compared to mere percentage of secondary structures. However the boundaries (and sequence lengths) of secondary structure patterns are not conserved, hence it is difficult to convert this information into feature patterns of fixed length suitable for training SVMs. This suggests that structural information alone may not be sufficient enough to distinguish cyclins from non-cyclins. This is also due to sharing of some common structural folds by cyclins and non-cyclin proteins, as is evident in the case of TFIIB and retinoblastoma proteins. However, remarkable accuracy of 92.14% was achieved with PSSM based SVM model. The embedded information of amino acids composition, their position-specific conservation and evolutionary relationship between different cyclins in the generated PSSM model makes it a robust model for achieving higher accuracy with strict maintenance at training-error level.

With a view to further enhance the prediction accuracy; we developed SVM classifiers trained with combinations of different features to generate hybrid models. We used amino acid, dipeptide, secondary structure composition and PSSM profile for generating the hybrid models. Amongst all the hybrid models developed, the SVM hybrid-6 model trained with secondary structure composition and PSSM profile optimized with RBF kernel, achieving the competing prediction accuracy with that of PSSM based model of 92.14% with sensitivity and specificity of 92.64% and 91.66%, respectively. Also, the AUC obtained from ROC curve was best for PSSM model and hybrid-6 model with 96.8%. It reflects that highly comprehensive but condensed and meaningful information is required to attain such high prediction accuracy.

However, the prediction accuracy on independent dataset; representing a true unknown prediction showed that PSSM based model was able to predict 53 out of 54 sequences (as compared to 50 by hybrid-6 and HMM profiles of Pfam database). Overall, PSSM based model was able to achieve an accuracy of 98.5% as compared to 95% by hybrid-6.

Thus, these results not only confirmed that optimized SVM is able to learn crucial features responsible for the accurate classification but also, it helps us to understand that prediction accuracy can be increased by providing more comprehensive information of a protein sequence. Moreover, our studies reiterate that biological patterns generally follow non-linear equations/functions which may not be easily predictable by conventional computational methods. This is fact is well exemplified by the classification task undertaken in our study in which we found that the models optimized with linear kernel functions are less accurate as compared to those trained with non-linear functions. For example, with linear kernel, the accuracy achieved in case of DPC based model was 81.43% as against 85.13% with RBF kernel. Similarly, PSSM based model achieved 92.14% accuracy when optimized with RBF kernel as compared to ∼91% with linear kernel.

The reason behind better performance of the model developed by us is mainly because we have used a statistical learning method which generates prediction models by learning from large complex datasets, also taking care of over-fitting problem. In most of the cases, a small fraction of total examples in the training dataset were used to gather all the information required for generating the classification model, thus the original dataset is filtered to make more informative and representative dataset in terms of Support Vectors (SVs) of representative sequences. In the study, the number of SVs finally chosen by SVM for training (PSSM and hybrid-6 based models) are 53 and 77 (out of 140 training sequences) which is just 38% and 55% of the total dataset, respectively. In the hybrid-6 model, training with information from both – the PSSM and secondary structure information as input to the classifier enhances both the prediction accuracy and reliability of the models generated.

Furthermore, the PCA analysis of the features has proven that the features which encapsulate comprehensive information about sequences help better segregation of training examples and enhances their correlation with the features under study. This is exemplified by the case of PSSM based model as compared to other feature based models.

The advantage with the present prediction method lies in the fact that classifier is independent of any sequence or structural similarity to any known proteins. The absolute inheritance of cyclin domains or motifs in proteins is not a mandatory object for prediction. The method can even predict those cyclins which have high sequence variability like cyclin M, T or some G2-specific cyclins. The results obtained from current implementation of features are highly encouraging. In future, more cyclin specific features may be included for enhancing the efficiency of the SVM model and subfamily specific classifiers may be developed. To develop subfamily-specific SVM classifiers, sufficient number of sequences representing each subfamily will be required. Currently, the number of cyclin sequences from each subfamily, is too low for developing subfamily-specific classifiers. Almost all the subfamilies have less than or equal to five sequences per group in the training dataset. Only cyclin B subfamily is represented by more than 30 sequences (Table S1, also see Table S2 for list of organisms and corresponding cyclin sequences in the training set). However, with the increase in the number of annotated cyclins in future, representing each subfamily, it should be possible to develop subfamily-specific SVM classifiers.

## Materials and Methods

### Data Source and Generation of Non Redundant Training Datasets

Classification efficiency of a SVM model depends a great deal on the quality of dataset used in training. The cyclin and non-cyclin sequences used as training dataset in the present work were obtained from different sources of manually curated and annotated sequence databases, including SwissProt [39], RefSeq [40] and other organism specific databases. Protein sequences annotated as ‘hypothetical’, ‘truncated’, ‘fragmented’ or ‘partial’ were excluded from the dataset. It is known that both transcription factor IIB and retinoblastoma proteins contain the cyclin associated conserved cyclin-box fold [11] and hence these proteins were included in the negative dataset. The manual filtering yielded a dataset of full-length and annotated cyclin and non-cyclin protein sequences.

Subsequently, the redundancy in the dataset was brought down to 30 percent using PISCES algorithm [41]. This implies that no two sequences in the training dataset are more than 30 percent identical to each other. After scaling down the redundancy, the final dataset used for training consists of 68 cyclin (Dataset S1) and 72 non-cyclin (Dataset S2) sequences. These sequences were used to generate features for the SVM training-testing protocol used in the study (available at the CyclinPred server). The percentage identity of most of the training dataset cyclin sequences lie between 15–25% (there is a pair which is 100% non-similar) whereas amongst non-cyclins most of the sequences are 15–20% similar. The identity of cyclin and non-cyclin sequences in the training dataset varies between 16–20%. The dataset includes cyclins from all cyclin subfamilies (20 different subfamilies); however the number of sequences representing each family was reduced due to sequence redundancy reduction. The highest score between most divergent cyclin sequence, Q9DEA3|PCNA_CHICK and any non-cyclin sequence is 18.9% identity with a sequence of transcription initiation factor IIB from *Guillardia theta* (closest non cyclin sequence). The lowest score is 0.8% identity with a Cytochrome b protein from *Dictyostelium discoideum*.

### Dataset for Blind-test performance

It has been reported that the performance of N-fold CV is not completely unbiased [21]. In order to assess the unbiased performance of our final SVM classifier, we evaluated its performance on a dataset not used in the training or testing. For this, we used a dataset of 61 false negative sequences from PROSITE database (cyclin sequences which do not have the cyclin PROSITE motif PS00292). Positive dataset training sequences common to these sequences were excluded; leading to an independent dataset of 54 cyclin sequences (Dataset S3). The accuracy, thus obtained reflects the true blind predictions for the test set. The performance of the best classifiers was also compared with available cyclin domain HMM profiles from the Pfam database (cyclin_N: PF00134, cyclin_C: PF02984 and cyclin: PF086 13).

### Assessment of training dataset for the presence of cyclin domain and motif

We performed pfscan search to look for PROSITE cyclin motifs in the 68 positive dataset sequences used in the SVM training. The cyclin motifs were found in only 35 out of the 68 cyclin sequences. We also found that 61 protein sequences had one of the cyclin associated Pfam domains. Comparisons of these annotated cyclin sequences showed that 6 proteins neither contain the Pfam cyclin domains nor the PROSITE cyclin motifs. This emphasizes the fact that all the known annotated cyclins are not represented by Pfam domain families and/or PROSITE signatures.

### SVM algorithm and problem formulation

SVM*^light^*, an implementation of Support Vector Machines (SVMs) in C language was used for the current study. The SVM*^light^* (http://svmlight.joachims.org/) allows optimization of SVM models by changing a number of parameters, including types of kernels (linear, polynomial, radial basis function or sigmoid) to perform the classification task. It is similar to solving a quadratic optimization problem (QP) and the decision function can be solved as
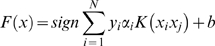
(1)where xi € R (real number) is the pattern used for the classification and y_i_ is the corresponding target value, which is +1 for cyclins and −1 for non-cyclins. α_i_ is the value provided by QP. The separating hyperplane generated by SVM model is given by
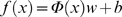
(2)Where, w is a vector normal to the hyperplane and b is a parameter that minimizes ∥w∥^2^ and satisfies the following conditions:
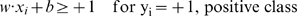
(3)

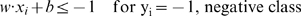
(4)


### Evaluation Methods

The entire dataset of ‘N’ number of sequences was divided into training and testing sets for which SVM parameters were optimized by Cross Validation (CV) method. The parameters were optimized by CV over the training set by maximizing the accuracy and minimizing the training error. In the study, we implemented three methods of evaluation to check the performance of classifier models, namely- Leave-One-Out CV (LOO CV) or Jackknife CV test, self consistency and holdout tests.

In the first method, the jackknife test is a CV technique in which one dataset sequence is used as a testing data (for validation of the generated model) while the remaining dataset sequences are used as the training data to develop a model. This is iterated N times till each sequence in the dataset become the testing data exactly once. In each of the iterations, the parameters corresponding to the best accuracy are recorded and then averaged for the final overall evaluation of the model. We also performed self-consistency test in which the entire dataset is used for the training as well as for testing to validate the generated model. This test indicates the confidence level of the model as it inherits the internal knowledge of the training dataset. Self-consistency test was carried out in two modes, differing in the use of kernel parameters. In mode-1, the performances of the models were checked by using the best kernel parameters as evaluated by jackknife CV test performed earlier. In mode-2, the performances of the models were checked by using new kernel parameters to achieve the best accuracy during the self-consistency tests.

The rationale behind using two modes was that mode-1 would provide the self-learning capability of the model on those parameters which have been obtained from cross-validation with jackknife test as well as this model will be used for prediction purpose whereas mode-2 would provide the self-learning capability of the model on those parameters which have not been involved any cross-validation or partitioning of the dataset and therefore, reflects the consistency of the prediction model.

In the third method of evaluation, namely the holdout test, the dataset is randomly split into two equal halves, with approximately equal numbers of positive and negative dataset sequences. SVM is then trained with one of these subsets with Jackknife CV test to find best parameters. The performance is then evaluated by testing the optimized parameters on the second subset. This test indicates the prediction efficiency of the models for sequences independent of the training.

We also evaluated the performance of the SVM model by the following measures:-

1. Sensitivity: percentage of cyclin protein sequences that are correctly predicted as cyclins.
2. Specificity: percentage of non-cyclin protein sequences that are correctly predicted as non-cyclin sequences.
3. Accuracy: percentage of correct predictions, for cyclins as well as non-cyclin sequences.
4. Mathew's Correlation Constant (MCC): employed for the optimization of parameters and evaluation of performance [42].

MCC = 1 signifies perfect prediction while MCC = 0 suggests completely random prediction. The above mentioned evaluations may be represented mathematically as given below.
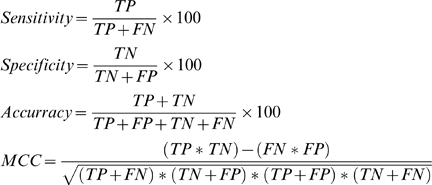
(5)where, True Positive (TP) and True Negative (TN) are correctly predicted cyclin and non-cyclin sequences, respectively. Similarly, False Positive (FP) and False Negative (FN) are wrongly predicted non-cyclin and cyclin protein sequences, respectively.

All the above mentioned measures are threshold-dependent i.e. the prediction performance is evaluated for a fixed SVM cutoff score or threshold. To calculate threshold-independent performance, a Receiver Operating Characteristic (ROC) curve was plotted between TP rate (sensitivity) and FP rate (1-specificity) [43]. The Area Under the Curve (AUC) describes inherent tradeoff between sensitivity and specificity and thereby measures the accuracy of the SVM model.

Another important consideration is whether the present prediction method is better than a random prediction. To check the reliability of the method, we first calculated R, an anticipated number of proteins that are correctly classified by random prediction [44]:
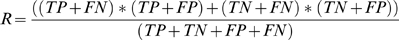
(6)


Subsequently, we also calculated S, the normalized percentage of correctly predicted samples better than random i.e. the method is independent of the scale of total samples in the dataset and R:
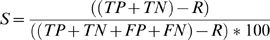
(7)Therefore, value of S = 100% stands for a perfect prediction and S = 0% for a worst prediction.

Among all common statistical measures like accuracy, specificity and sensitivity, F1 statistic is a more robust measure as other measures can overstate the performance of the classifier. F1 statistic is a harmonic mean of precision and recall (or between sensitivity and positive predictive value), given by equation 8:
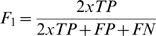
(8)Finally the best SVM model on the basis of accuracy, MCC, S (random prediction), F1 statistic and other statistical measures is validated against the test set not used in the training using the blind test independent datasets.

### Calculation of Protein Features

#### Amino acid composition (AAC)

The following equation was used to calculate AAC,
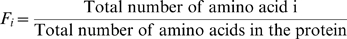
(9)where *i* is any of the 20 amino acids.

#### Dipeptide composition (DPC)

The fraction of each dipeptide in the protein was calculated by the following formulae,
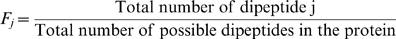
(10)where *j* can be any of the 400 dipeptides.

#### Secondary Structure Composition (SSC)

Secondary structure is an important feature of cyclins due to its characteristic helical domains assuming helix rich cyclin-folds, containing the cyclin-box [13], [45]. Secondary structure prediction was carried out by PSIPRED v2.4 which is a simple and reliable prediction method. The method incorporates two feed-forward neural networks that perform an analysis on the output obtained from PSI-BLAST v2.2.4. PSIPRED predicts secondary structure for each residue and provides a confidence score for three types of secondary structures: helices, β-sheets and coil regions. For each of the amino acids, the scores corresponding to helix, sheet and coils are extracted and averaged respectively, thereby making a matrix of 60 (20×3::amino acids×secondary structures). Following equation was used to calculate the features corresponding to secondary structure prediction,
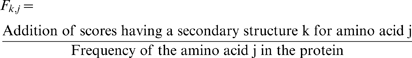
(11)where *k* can be any of the 3 secondary structures (helix/sheet/coil) and *j* can be any of the 20 amino acids.

#### Position Specific Substitution Matrix (PSSM) profile

This model was designed using PSI-BLAST v2.2.4 output. For each amino acid, PSI-BLAST give 20 substitution scores in the PSSM which provides the evolutionary information about the protein at the level of residue types. Three iterations of PSI-BLAST were carried out at cut-off E-value of 0.001. Each value in the PSSM represents the likelihood of a particular residue substitution at a specific position of a protein class and it was normalized between 0 and 1 using the logistic function as shown in equation 12 [46]

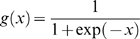
(12)


Where x is the raw value in PSSM profile and *g(x)* is the normalized value of x. Following this, the normalized matrix is organized into a composition matrix of fixed length pattern of 400 (20×20, for each amino acid, there are 20 substitution scores from normalized matrix). Normalization was done for other features also.

### Principle Component Analysis (PCA)

PCA leads to a linear combination (projection) of the original variables of high-dimensional data and thereby, is used to calculate orthogonal variables from raw data matrices. PCA analysis was performed to identify different feature element variables from AAC, DPC and PSSM that are important for distinguishing cyclins from non-cyclins. Thus, it was performed with the objective of obtaining new variables that are uncorrelated among themselves (i.e. orthogonal) from the original ones and to reduce the dimension of data to unity with the minimum loss of information. Using “princomp” function of MATLAB, we extracted the PCs by using the same data matrices which were also used as input for SVM training. The scatter plots of scores of first two principal components for AAC, DPC and PSSM matrices are shown in the Figure 3A, C and D.

## Supporting Information

Table S1 Training data set sequences from different cyclin subfamilies used in training the SVMs
(0.04 MB DOC)

Table S2 List of organisms and corresponding cyclin sequences included in the training data set. Cyclin sequences from 58 distinct organisms have been used in the training data set.
(0.07 MB DOC)

Dataset S1 Cyclin sequences (Positive data set) used for training the SVMs using different features.
(0.06 MB DOC)

Dataset S2 Non cyclin sequences (Negative data set) used for training the SVMs.
(0.08 MB DOC)

Dataset S3 Independent sequences (Independent data set) used for blind-test performance.
(0.05 MB DOC)
