# Supplementary material for: CyclinPred: A SVM-Based Method for Predicting Cyclin Protein Sequences
Source: PLoS One. 2008 Jul 2;3(7):e2605. doi: 10.1371/journal.pone.0002605 (PMC2435623; doi:10.1371/journal.pone.0002605)
Supplement: Table S1 — Training data set sequences from different cyclin subfamilies used in training the SVMs (0.04 MB DOC) [file pone.0002605.s001.doc]

**Figure S1. Training data set sequences from different cyclin subfamilies used in training the SVMs**

|  | | | |
| --- | --- | --- | --- |
| **S. No.** | **Cyclin Subfamilies** | **Number of Sequences** | |
|  |  | **Before 30% Redundancy** | **After 30% Redundancy** |
| 1 | cyclin-A | 17 | 8 |
| 2 | cyclin-B | 40 | 32 |
| 3 | cyclin-C | 13 | 5 |
| 4 | cyclin-D | 16 | 4 |
| 5 | cyclin-E | 13 | 5 |
| 6 | cyclin-F | 6 | 3 |
| 7 | cyclin-G | 8 | 2 |
| 8 | cyclin-H | 5 | 4 |
| 9 | cyclin-I | 3 | 1 |
| 10 | cyclin-J | 1 | 1 |
| 11 | cyclin-K | 2 | 1 |
| 12 | cyclin-L | 8 | 4 |
| 13 | cyclin-M | 8 | 2 |
| 14 | cyclin-S | 2 | 2 |
| 15 | cyclin-T | 9 | 4 |
| 16 | cyclin-V | 2 | 1 |
| 17 | cyclin-Nterm | 26 | 8 |
| 18 | cyclin-PCNA | 9 | 2 |
| 19 | cyclin-mitotic | 9 | 5 |
| 20 | cyclin-Others | 22 | 11 |
|  | **TOTAL** | **219** | **105** |
