# Supplementary material for: CyclinPred: A SVM-Based Method for Predicting Cyclin Protein Sequences
Source: PLoS One. 2008 Jul 2;3(7):e2605. doi: 10.1371/journal.pone.0002605 (PMC2435623; doi:10.1371/journal.pone.0002605)
Supplement: Table S2 — List of organisms and corresponding cyclin sequences included in the training data set. Cyclin sequences from 58 distinct organisms have been used in the training data set. (0.07 MB DOC) [file pone.0002605.s002.doc]

**Figure S2. List of organisms and corresponding cyclin sequences included in the training data set. Cyclin sequences from 58 distinct organisms have been used in the training data set.**

| **Organism** | **Sequences** |
| --- | --- |
| *Aeropyrum pernix* | PCNA |
| *Anguilla japonica* | Cyclin-B2,B1 |
| *Antirrhinum majus* | G2/Mitotic-cyclin1,cyclin2 |
| *Arabidopsis thaliana* | Cyclin-B1,D1,D2,D3,PCNA |
| *Arbacia punctulata* | G2/Mitotic Cyclin-B |
| *Asterina pectinifera* | G2/Mitotic Cyclin-B |
| *Bos Taurus* | G1/S-Cyclin -D1, Cyclin-T1,G1 |
| *Caenorhabditis briggsae*. | G1/S-Cyclin-E |
| *Caenorhabditis elegans*. | Cyclin-H,B,L,T,T1,G2/Mitotic-CyclinA1,B1,G1/S-CyclinE |
| *Candida albicans* | G2/Mitotic-Cyclin CYB1,G1/S-Cyclin-CLN1,CLN2 |
| *Canis familiaris* | G1/S-Cyclin-D1,G2/Mitotic-Cyclin-B3 |
| *Carassius auratus* | Cyclin-A1, G2/mitotic-specific cyclin-B1 |
| *Catharanthus roseus* | PCNA |
| *Chlorohydra viridissima* | G2/mitotic-specific cyclin-B |
| *Colletotrichum gloeosporioides* | G1/S-specific cyclin |
| *Danio rerio* | Cyclin-A1,A2,B2,G1,L,T2,F,I, G1/S-specific cyclin-E1,D1 |
| *Daucus carota* | PCNA |
| *Dictyostelium discoideum* | G2/mitotic-specific cyclin-B |
| *Drosophila melanogaster* | Cyclin-T, G2/mitotic-specific cyclin-A,B, G1/S-specific cyclin-E |
| *Emericella nidulans* | G2/mitotic-specific cyclin-B |
| *Encephalitozoon cuniculi*. | G2/MITOTIC SPECIFIC CYCLIN 2 |
| *Gallus gallus* | PCNA,Cyclin-D3,E2,,K,S,A2,L1, G2/mitotic-specific cyclin-B2,B3, G1/S-specific cyclin-E1,D1 |
| *Glycine max* | G2/mitotic-specific cyclin S13-6 |
| *Guillardia theta* | Cyclin-B |
| *Halobacterium salinarium* | PCNA |
| *Hemicentrotus pulcherrimus* | G1/S-specific cyclin-E |
| *Homo sapiens* | Cyclin-B3,I,M2,J,M3,M4, A2,A1,T2,K,G2,L2,F, G2/mitotic-specific cyclin-B1,F,B2, G1/S-specific cyclin-E1,D2 |
| *Marthasterias glacialis* | G2/mitotic-specific cyclin-B |
| *Medicago varia* | G2/mitotic-specific cyclin-1,2 |
| *Methanopyrus kandleri* | PCNA |
| *Mesocricetus auratus* | Cyclin-A2, G2/mitotic-specific cyclin-B1 |
| *Mus musculus* | Cyclin-G2,C,B3,D,L1,M1,M2,M3,M4,L2,A1,A2, G2/mitotic-specific cyclin-B1,B2,B3,F, G1/S-specific cyclin-E,E1,E2 |
| *Oryza sativa subsp. Indica* | Cyclin-B2 |
| *Oryza sativa subsp. japonica* | PCNA,Cyclin-C1,B2 |
| *Oryzias javanicus* | G2/mitotic-specific cyclin-B2 |
| *Oryzias luzonensis* | G2/mitotic-specific cyclin-B1,B2 |
| *Pan troglodytes* | Cyclin-T1 |
| *Paramecium bursaria chlorella virus* | Mitotic cyclin-CYC1a,PCNA |
| *Patella vulgate* | G2/mitotic-specific cyclin-A,B |
| *Plasmodium falciparum* | Cyclin-4,PCNA |
| *Populus nigra* | PCNA |
| *Pyrobaculum aerophilum* | PCNA |
| *Pyrococcus Kodakaraensis* | PCNA |
| *Rana japonica* | G2/mitotic-specific cyclin-B2 |
| *Rattus norvegicus* | Cyclin-G1,H,A2,PCNA, G2/mitotic-specific cyclin-B1, G1/S-specific cyclin-D1,D2,D3,E |
| *Saccharomyces cerevisiae* | Cyclin CCL1,pch1, CLG1,1,2, Meiosis-specific cyclin rem1, G1/S-specific cyclin pas1,5,6,CLN1,CLN2,CLN3, G2/mitotic-specific cyclin cdc13,cig1,1,2,3,4,cig2 |
| *Sarcophaga crassipalpis*. | PCNA |
| *Schizosaccharomyces pombe* | G2/mitotic-specific cyclin cdc13,cig1,cig2, G1/S-specific cyclin pas1, Meiosis-specific cyclin rem1, Cyclin pch1 |
| *Spisula solidissima* | G2/mitotic-specific cyclin-A,B |
| *Sulfolobus acidocaldarius* | PCNA |
| *Styela clava* | PCNA |
| *Thermoplasma Volcanium* | PCNA |
| *Toxoplasma gondii*. | cyclin-H |
| *Trypanosoma brucei*. | Cyclin-5,8, G2/mitotic-specific cyclin-1, |
| *Trypanosoma cruzi*. | Cyclin-4,5,6 |
| *Xenopus laevis* | Cyclin-I,G1,F,H,E2,G2,A1,A2, PCNA, G2/mitotic-specific cyclin-B1,B2, G1/S-specific cyclin-D1,D2,E3 |
| *Leishmania Major* | Cyclin N-terminal,10,3 |
| *Eimeria tenella* | PCNA |
