# Supplementary material for: CyclinPred: A SVM-Based Method for Predicting Cyclin Protein Sequences
Source: PLoS One. 2008 Jul 2;3(7):e2605. doi: 10.1371/journal.pone.0002605 (PMC2435623; doi:10.1371/journal.pone.0002605)
Supplement: Dataset S1 — Cyclin sequences (Positive data set) used for training the SVMs using different features. (0.06 MB DOC) [file pone.0002605.s003.doc]

**Data set S1. Cyclin sequences (Positive data set) used for training the SVMs using different features.**

>Q9DG97|CCNB1_ORYLU G2/mitotic-specific cyclin-B1 - Oryzias luzonensis (Luzon ricefish).

MALRVTRNRLASARAELGGKTCSVAGPTQKPRAALGEIGNVAVINKDVTKKNVKTEVAKKTKVPAKAEKIEQPKAAVVPVKPAPEVQVPAQPEPASPTPMETSGCEPADLCQAFSDVILNTAIRDVDADDYNNPLLCSEYVKDIYKYLRQLEVEQSVKPNYLEGQEVTGNMRALLIDWLVQVSLKFRLLQETMYMTVGIIDRFLQDHPVPKKQLQLVGVTAMFLASKYEEMYPPEISDFAYVTDKAYTTAQIRDMEMTILRVLKFQLGRPLPLQFLRRASKIYEVTAEQHTLAKYLLELSMVDYAMDHFPPSMVASAALALTLKVLDAGEWDVTLQHYMAYTADTLTPVMAHIAKNVVKVNNGQTKHMTIKGKYFTSKQMRIATIFQLKSSVVKDLATQIS

>Q8TWK3|PCNA_METKA DNA polymerase sliding clamp - Methanopyrus kandleri.

MVEFRAYQEEARYFKYAFNAAGKVVEEAPLIVTENGIVSRAMDASHIAMAVLEMPWEMFDEYEPPSDELMYGLDMEEVTRIVRRARVTDEITLEGEDEEEVIIKLGSSGYEREFRLRSIDIDDIPDEPELDFAVEVTVVPDFIQDAVRDADLVSDTVKVGAKGNTFYFKAEGERGRVIPKVQEGAEALLTFEVEEDVETAYPLDYLKDMIQAAQGAESVRIRLGQDMPLELTFRIGPAGEGKLTFYLAPRVEE

>Q9ZW35|PCNA2_ARATH Proliferating cell nuclear antigen 2 - Arabidopsis thaliana (Mouse-ear cress).

MLELRLVQGSLLKKVLEAVKDLVNDANFDCSTTGFSLQAMDSSHVALVSLLLRSEGFEHYRCDRNLSMGMNLGNMSKMLKCAGNDDIITIKADDGSDTVTFMFESPTQDKIADFEMKLMDIDSEHLGIPDAEYHSIVRMPSGEFSRICKDLSSIGDTVVISVTKEGVKFSTAGDIGTANIVLRQNTTVDKPEDAIVIEMNEPVSLSFALRYMNSFTKATPLSETVTISLSSELPVVVEYKVAEMGYIRYYLAPKIEEEEDTKPE

>Q9GP34|CCNH_ECHMU Probable cyclin-H - Echinococcus multilocularis.

MAYVTSTQLYNWRFTQDELANQRQECNAAVRRRLSAQYRAKAEVKGGDVPAEPTFLTAEEEFIIVKRYIFAMKELFHQFSDSGLPVDVFGFAATYLKRFYLNNSVMDYFPREMMLTALYLACKAADYPLGLQTFAAHIPRNREHYSEIVVHSELFLMEKLQYDIWIHTPYRPLSGLLVDLLAYRKTQLGEPMTIEAGEVATADMMTSLKKEGYEIIHKWFQTDLCLTHSPSQFALAVLLELGQKHPDLGVEKFVKNSVCEYNSSDELMEQKWTALNEKMEQIQTMIGEFEFLSDLTYGSDLEAVLMQCRNPLYDPLSEEYAAAKEQAEKLMSFLD

>O10308|PCNA_NPVOP Probable DNA polymerase sliding clamp - Orgyia pseudotsugata multicapsid polyhedrosis virus (OpMNPV).

MEATFATAAAFQSIVGALRGLLTHATFDCDAHGMRLHSLDVERVALADLRLRRAGFARYACERKLSFSVPVRGLVKIVRTADPSAPLTMRVAARDDRVRLAYETARRAVSCTLAQISLDADRLGVPDKEYTCVLAVPSAELTRACADLARLGATTVEMSSGTTGLSFAAHADDGVRVRVLLRGSPQRPLTQAFACCYLNKLARASALSETVDVCMDASMPLRLRFRLGPLGALDLYLAPRVPSVGESAQ

>Q5ZJP9|CCNL1_CHICK Cyclin-L1 - Gallus gallus (Chicken).

MAAGSGSAAAVAAVAGGGPAGPHAAGVTAGAVTTGSGAPVPGPGAVLIGDRLYSGVLITLENCLLPEHTLRFTPSMSSGLDPDTETELRVTGCELIQAAGILLRLPQVAMATGQVLFQRFFYTKSFVKHSMEHVSMACVHLASKIEEAPRRIRDVINVFHRLRHLREKKKPVPLILDQEYVNLKNQIIKAERRVLKELGFCVHVKHPHKIIVMYLQVLECERNQHLVQTSWNYMNDSLRTDVFVRFQPESIACACIYLAARTLEIPLPNRPHWFLLFGTTEEEIQEICLKILQLYTRKKVDLSDLESKIEKKKLAIEEAKAQAKGLLPEGAPVLDNTSGFSPLPKNESPKEVKGNKPSPLPVQAMKNAKRKAEGAKRTGSNSPVNGVQKGRESRSRSGSRDQSYSRSPSRSASPKHRKSESYSTSSGSKSHSRSRSRSGSPPRQFNHSSTYKSSKMRSYKKSKDYKYSAHKARKSRSRSSSRSRSRSRERSDHSGKYKKKSHYYRNHRHERSRSYERASHRYDRDHPGHSRHRR

>Q06712|PREG_NEUCR Nuc-1 negative regulatory protein preg - Neurospora crassa.

MLTRSPASAAATSPTTTVTVTVTAAATSPRPSRGAAPHGHFHYAPTPSLREAASRIPALSSRRQSATAPATSSTSLPISIQSRHGPAPVASHTSSPVASDPGLGFPPSSRPQRWAGVDVVAQYSPMEPMDYTTGALLSSQAASSSNNQAVARGSIESRPSHIPNTVGPMASHEEQASALASTSEATAAPIPHGPTVAVAVPPTPGVYSAAPQPHPLSHTPQTGTPVKRRNSQGDVTHNQVEAASAGQSGPHSPKRARLAPKIVSRRYEHCPVEDLVVLIAHMLGELIELNDEAAQKVGQRHNLTRFHSRTTPGISVLDYLHRLAKHAYLSPPILLSMVYYIDRLCALYSDFTINTLTVHRFLITAATVAAKGLSDSFLTNTLYARVGGVRVAELNMLELEFLHRVDWKIVPDPDVLVAYYGGLVARCPGYILECPEPEEADDEDEDEELDESDAIGDDDDDIDGEGGEREEETSSSQRDRHAT

>P41179|CCN1_TRYBB G2/mitotic-specific cyclin-1 - Trypanosoma brucei brucei.

MMTNLNVRTIKGCFPGSLPDALNDMSVINHILSLTGRFDEGQAALASTVNVVYVGTAVYDIPRYREEYTKNFIARGCGISEVCVAEARSTGATPCAAATMVTPDQLQHLAEAHIILLPEVTPFSLYDAGRKRASMRVSGQVAARGVVLVGGGCCFRAEHSDSANPKTCAQYMLSRENEVDSGRPVEMEEGGAKWEYLCVHGLSVLPGIFCPQHSSRDATGLLLNESFSKMLKRHPTERGIGVDCRAVLLLMGDGRYQVLTIANREGRTASVKDINIQIKDVVEGNVQTTTIQQQGSVEELLRKPCGPVVRDPFEAYYAMANPTALTEKLLCAPR

>Q8ZTY0|PCNA1_PYRAE DNA polymerase sliding clamp A - Pyrobaculum aerophilum.

MAKQVLTYIDAKEFAYIIDSISVLVEEANFLIRNDGLYLRALDVSRTAMVDLAIPKESFEEFPEVEELRFGLNFKELKKLLRRVKKGDKISMEFEEGRVRIKLIGKSVRSIVVPSIEVVGEELPTPKVVYTAMVKAASDVLATAVKDADAVADEVKFEASEEALIISASSDKGEVEVKLDKNSELVYEFDVKEPASARFSLEYLVDITSKTSKISDIVTIELATAKPIYLSFDIPAGGKISYFIAPRVE

>Q382D4|Q382D4_9TRYP Cyclin 9 - Trypanosoma brucei.

MAGFTETPSWPSLSCEQSSAAAVSVSYSPFVVEMSYYTIAIQAYSMTRYVVENLRLDSAVVGTAMTYWHIFVSVHGLKKVDEIVLSAACTFLASKVEHHKVRLSDIVALVFEVDPVGGVMESWRDVVGQAELLLCYTLKFNFQVIHPVNRINELVFDGNKAVLECAQRLFLLSFVTPLCTRASAEEIVEALVYLAADGAERLEVYANGFNITSSERRDGIISVMLDALVAMRKTTKLPKIDNVISARRKRLREQESTRPSVACSSVGTASTDNTPCAPSVE

>P24870|CG23_YEAST G2/mitotic-specific cyclin-3 - Saccharomyces cerevisiae (Baker's yeast).

MHHNSQSLSSGHIRSPEDENVAPIGNLKHRTGSLSHISSAHPRVALSDVTNIVATNSSNNSISKPKVAPIKERLDSAAIIEEERLDANSVAQRKEADHNDLLTDREQEEPVEDDGESEEDEEEDQEPLLLQHYASDTLVWEHAFRTYYRTTLDPNDDDVYDVVMVAELSNEIFEYMRKLEDLYKPNPYYMDKQPELRWSFRSTLIDWIVQVHEKFQLLPETLYLCINIIDRYLCKEVVPVNKFQLVGAASLFIAAKYEEINCPTIKDFVYMSENCYSRNDLLDAERTILNGLEFELGWPGPMSFLRRISKADDYEHDTRTLAKYLLESTIMDHRLVSAQPSWLAAGAYFLSKIILGQNQWSLAHVYYSNYTQEQILPLATIILENCRYASKRHNAIWRKYSSRRYLHSSQIVAKWIALAEHRVERSN

>Q4Q9F8|Q4Q9F8_LEIMA Mitotic cyclin, putative - Leishmania major.

MPAYVVAKQEAERMRKEAWRPLTFRMIQQRIRDHFVRDLDDETELRSSRYILTTAQVERFLFPLFQRADAKAVRVLGEVWGRSRDPSRKLSDQIVAVLTRRQHVLLQGTELTLMELKEKVLLAARLQEPLTACEVRQLAIQLGPSNRVWVEEWLRAHPAEEAVDSLALCTALRDAVQQRFGAFTFSGAYYPTVLDDLIDMDELAQSSMVYPPKQGVPAQSVRARACEELFIFTIFCGVPLSLDAYFLAVALLDRFLARRSTPKEQLRLYSMAALLLASKCDHSWPTLDPHFVSVKMKLAQEDVMTAEEEIVQTLQFDTTVSTLHHFCEALLLHQDPPASPEQRQLLEYLIASLSVHTYYGQYRQSCLAAAALHSSRHAARLATGEPSESVRVLLPVVYAALQKNNVERSPGNLIKQIYAQPERHAVSLTPTAVLFPSLSCCSSLSASE

>P41002|CCNF_HUMAN G2/mitotic-specific cyclin-F - Homo sapiens (Human).

MGSGGVVHCRCAKCFCYPTKRRIRRRPRNLTILSLPEDVLFHILKWLSVEDILAVRAVHSQLKDLVDNHASVWACASFQELWPSPGNLKLFERAAEKGNFEAAVKLGIAYLYNEGLSVSDEARAEVNGLKASRFFSLAERLNVGAAPFIWLFIRPPWSVSGSCCKAVVHESLRAECQLQRTHKASILHCLGRVLSLFEDEEKQQQAHDLFEEAAHQGCLTSSYLLWESDRRTDVSDPGRCLHSFRKLRDYAAKGCWEAQLSLAKACANANQLGLEVRASSEIVCQLFQASQAVSKQQVFSVQKGLNDTMRYILIDWLVEVATMKDFTSLCLHLTVECVDRYLRRRLVPRYRLQLLGIACMVICTRFISKEILTIREAVWLTDNTYKYEDLVRMMGEIVSALEGKIRVPTVVDYKEVLLTLVPVELRTQHLCSFLCELSLLHTSLSAYAPARLAAAALLLARLTHGQTQPWTTQLWDLTGFSYEDLIPCVLSLHKKCFHDDAPKDYRQVSLTAVKQRFEDKRYGEISQEEVLSYSQLCAALGVTQDSPDPPTFLSTGEIHAFLSSPSGRRTKRKRENSLQEDRGSFVTTPTAELSSQEETLLGSFLDWSLDCCSGYEGDQESEGEKEGDVTAPSGILDVTVVYLNPEQHCCQESSDEEACPEDKGPQDPQALALDTQIPATPGPKPLVRTSREPGKDVTTSGYSSVSTASPTSSVDGGLGALPQPTSVLSLDSDSHTQPCHHQARKSCLQCRPPSPPESSVPQQQVKRINLCIHSEEEDMNLGLVRL

>P46277|CCNB1_MEDVA G2/mitotic-specific cyclin-1 - Medicago varia (Alfalfa).

MKFSEEKNVSNNPTNFEGGLDSRKVGQNRRALGVINQNLVVEGRPYPCVVNKRALSERNDVCEKKQADPVHRPITRRFAAKIASTKTSNAEGTTKRSNLAKSSSNGFGDFIFVDDEHKPVEDQPVPMALEQTEPMHSESDQMEEVEMEDIMEEPVMDIDTPDANDPLAVAEYIEDLYSYYRKVESTSCVSPNYMAQQFDINERMRAILVDWLIEVHDKFDLMHETLFLTVNLIDRFLEKQSVVRKKLQLVGLVAMLLACKYEEVSVPVVGDLILISDRAYTRKEVLEMEKVMVNALKFNISVPTAYVFMRRFLKAAQADRKLELLAFFLIELSLVEYAMLKFSPSQLAAAAVYTAQCTMYGVKQWSKTCEWHTNYSEDQLLECSSLMVDFHKKAGTGKLTGAHRKYCTSKFSYTAKCEPASFLLENEL

>Q4QAE2|Q4QAE2_LEIMA Cyclin 10 - Leishmania major.

MQIGLAGCSSSLHTSPSSGSAAGSGFYGARGQEAAASATVNSSGANGLCAGATMSSPSATPTLPPLPSTMDEPVDLFPLTPSVSMLQRHSKRSGRLLNESETMQEKHGQEYRFLVPWLAYAIDCTIAAHEVLRQKHGIPAHVSSPASPSANTLNGATDVKRSSRGSANGDPTYADTAGGSSGSGQAALPSPQAELNAFSTREVPAISVHDYLKRIVKYTYVSPSVLVCGCLYLDRLLCMHPCMLLHPYNVFKLFLTSTRMASKIMDTRTLNNHDFSVVGGVTNDDLNALEFLMVELLQNRLYFSRDTFDEYCRPLRLQAAHLTEEASDWGAETAMETPVETRSGVQHQPTRPFLARCNGSNTRSRRSSILSQNEYSVSIASQSGVSPLRSASRPAFPPSASTARSISVDTESIGPARAPASRGGSASTSAPRHPLGTSYSPALPHVPHTAANGGSSPALGAMGGLSGRGASGEDMLRVATHTNSIYQSSNGVWTGQTPPDSVTVLVGVEERGDERARSISLSAPRCGATTSWDATGRPSRLLAASTSTMAGLCTAALNGGRGGYESSPNVCAAPTYNRASASSSRGITASAEDFGAGSMVISYSDRTPTRRAFSHASREGIALPPVVCSGRSERSASVIGVAAQGQRLPPPPDQPRV

>Q9Y9V7|PCNA2_AERPE DNA polymerase sliding clamp B1 - Aeropyrum pernix.

MFRLVYTASSKFKYIAQTLAKINDEGVFEFSLDGLRAWIMSPDKTSLAILEMPSLSFEEYMVEEEMRVVLRTDELNKISKRATRNDDIIFQWNAEEQALEVELRDRKLGFSRKFLVPATSVGAEEMRRLKLEPTVSFTILTDDLKAMIQDVKVVGDFAEFEASEGQVVVRSQAEEKEYEWVMKPGDVLLSLEVEEDAKSIYSRQVLEIATKPVGAAESVKVSFASDYPMKIEYTFPNGERMELYMAPSLAG

>Q4QF35|Q4QF35_LEIMA Proliferating cell nuclear antigen - Leishmania major.

MLEAQVQYASLWKRLVECINGLVNEANFDCNPGGLSIQAMDSSHVALVHMLLRDDCFVKYQCERNIILGLNLASLSKVLKIVDGNDSLSLRHDDDSDVVTLTSENPEKTRKCEYQLKLLEIEAESMGIPEMDYRSTVTLNSAEFAKIVRDMQVFGDTVTIAISKEGVKFSSSGDVGQGYTFLQAAGVSDRSAKSEVKSEVKAEARDEDEHEPISRRYNKAEGGNGAIGVEVAMEEPITLSFALRFMGIFAKGSTLSERVTLKFAKDSPCMVEYGIDNVGYLRYYLAPKVDDAE

>Q4J9A8|PCNA2_SULAC DNA polymerase sliding clamp 2 - Sulfolobus acidocaldarius.

MKVKVVDALGFSYIFKTLSQYVSEATLLFGNDGFKVKGMDPSKVVYIDIFVPKDYFEEYNLENEMKIGVSLKDVNEVIKNVSKEDILYLELEKDKIMFTLDGEYLRTFSLPVLSPDEVETPSINLEFPFRANILTSTFGDLLDEFDQIGGDSIRFKAQNGKLYLSVMGDMGESIVELSLENGGLLESTGTDAESLYGLEHVSNTTKMRRPSDTLEIAFGSQLPLKLRYNLPKGGYADFYIAPRSE

>P93411|CCC11_ORYSJ Cyclin-C1-1 - Oryza sativa subsp. japonica (Rice).

MAANFWTSSHCKQLLDQEDVDKVPQADSDRGITLEEFRLVKIHMSFHIWRLAQQVKVRQRVIATAVTYFRRVYTRKSMTEYDPRLVAPTCLYLASKVEESTVQARLLVFYIKKMCASDEKYRFEIKDILEMEMKLLEALDYYLVVYHPYRPLLQLLQDAGITDLTQFAWGIVNDTYKMDLILIHPPYMIALACIYIASVLKDKDITLWFEELRVDMNIVKNISMEILDFYDTYKIDPQRGLPEDKIAPVMNKLPSKA

>Q2KI22|CCND1_BOVIN G1/S-specific cyclin-D1 - Bos taurus (Bovine).

MAHQLLCCEMETIRRAYPDANLLNDRVLRAMLKAEETCAPSVSYFKCVQKEILPSMRKIVATWMLEVCEEQKCEEEVFPLAMNYLDRFLSLEPVKKSRLQLLGATCMFVASKMKETIPLTAEKLCIYTDNSIRPDELLHMELVLVNKLKWNLAAMTPHDFIEHFLSKMPVAEENKQIIRKHAQTFVALCATDVKFISNPPSMVAAGSVAAAAQGLHLGSANGFLSYHRLTRFLSKVIRCDPDCLRACQEQIEALLESSLRQAQQQNLDPKAAEEEEEEEEVDLACTPTDVRDVNI

>Q9DEA3|PCNA_CHICK Proliferating cell nuclear antigen - Gallus gallus (Chicken).

MFEARLVQGSVLKRVLEALKDLITEACWDLGSGGISLQSMDSSHVSLVQLTLRSEGFDTYRCDRNIAMGVNLNSMSKILKCAGNEDIITLRAEDNADTLALVFEAPNQEKVSDYEMKLMDLDVEQLGIPEQEYSCVVKMPSAEFARICRDLSHIGDAVVISCAKDGVKFSANGELGNGNIKLSQTSNVDKEEEAVTIEMNEPVQLTFALRYLNFFTKATPLSPTVTLSMSADVPLVVEYKIADMGHLKYYLAPKIEDQQEGS

>Q9HN45|PCNA_HALSA DNA polymerase sliding clamp - Halobacterium salinarium (Halobacterium halobium).

MFKAIVSADTLQETLDSVSVLVDECKIHLDDDTLSIRAVDPASVGMVDLDLAATAFESYEADGGLIGVNLSRLEDIAGMADAGQLIQLELDEETRKLHIQIDGLEYTLALIDPDSIRQEPDIPDLDLPAHVAIEGRDIDRAVTAADMVSDHIALGVDTGDDLFYVNAEGDTDDVHLELAPDQLIDLDAGDAHSLFSLDYLKDMNKAIPTTAEVELELGDEFPIKLHFDIADAQGHVTYMLAPRIQSN

>P39948|CCND1_RAT G1/S-specific cyclin-D1 - Rattus norvegicus (Rat).

MEHQLLCCEVETIRRAYPDTNLLNDRVLRAMLKTEETCAPSVSYFKCVQREIVPSMRKIVATWMLEVCEEQKCEEEVFPLAMNYLDRFLSLEPLKKSRLQLLGATCMFVASKMKETIPLTAEKLCIYTDNSIRPEELLQMELLLVNKLKWNLAAMTPHDFIEHFLSKMPEADENKQIIRKHAQTFVALCATDVKFISNPPSMVAAGSVVAAMQGLNLGSPNNFLSCYRTTHFLSRVIKCDPDCLRACQEQIEALLESSLRQAQQNIDPKATEEEGEVEEEAGLACTPTDVRDVDI

>Q4U9A0|Q4U9A0_THEAN Cyclin, putative - Theileria annulata.

MNCHRDDMVEKNNVSGIEFDWVKNSTHYKNWLIDSIDKLNSIQLNTYNNAVKLLQKRKFEYKIPSFNDELWLIKYYSFQLSQFVNSNNLKPSVKETSLVLFNRFYLRRSLLEYDPRIIMFTCITLATKLEDMWRSVYIDKLLYKINNLNITKVFEMESIVCDVLNFNLNILHLNDSIYILIQLLISYIKESLDVDGINEYIGIILYMLKEAEMSALIMNESPSLTFLYTPPQLTLSIILQLIKNTSLSTILSIESFVLKLLNGDEKNLMKLLSLTQSIMDEYNEFLAFRNELNQSKALPTPKCMVISFYSQVYTNKSIE

>Q9MAY3|PCNA_POPNI Proliferating cell nuclear antigen - Populus nigra (Lombardy poplar).

MLELRLVQGSLLKKVLESIKDLVNDANFDFSSSGFSLQSMDSSHVALVALLLKSEGFEHYRCDRNTSMGMNLGNMSKMLKCAGNDDIITIKGDDGSDTVTFMFESPTQDKIADFEMKLMDIDSEHLGIPEAEYHAIVKMPSAEFARICKDLASIGDTVVISVTKEGVKFSTRGDIGTANIVLRQNTTVDKPEDATVIEMNEPVSMTFALRYMNSFTKATPLSNTVTISMSPDLPVVVEYKIAEMGYVRFYLAPKMEEDEPEPGA

>Q84513|PCNA1_PBCV1 Probable DNA polymerase sliding clamp 1 - Paramecium bursaria Chlorella virus 1 (PBCV-1).

MDDDNVLFHIRTLQGNVIKSLFDCLKEILHDVMLSFGPTGIRISALDGAKVSLVHLKLDSESFEEYKCEHTYELGVNVLNMFKLLRSAGSHDSILFRYLKNDPHMIELTIQNFEKNSLTKFNMKLIEIDSVEIEVGDIEFDTIIVMPANYFQRICRDMSDITDHLVIVKKGDEVSFNSDYTCVTDFASQKTIIGDSDNGQITCNNDTDYESKFSLKYLTSFCKASGMSSAVEIYLKESYPLILKYTVGSMGALKFVIAPILS

>Q7KQJ9|Q7KQJ9_PLAF7 Proliferating cell nuclear antigen, putative - Plasmodium falciparum (isolate 3D7).

MFECRIDGQFFKKLFETLKDICTEVNLECDENGIKMQSMDCSHVSLVDLNIVSDFFQHYRCDKNCVLGISINFMLKILSVIKEKSTVFLFKEDNENDAVLNIGIIDEEEQSSADDSLEIQVKLINAQKEHLEIPQSEYHCQCTMKSKKFQEFTKYLNSIGDNVSISMKKDAMILSTTGSDIKVTKQFTNDMTDISITCTKSVSQEFATRYLVMFSRASSLSDEVLISLSPHIPISIKFNFKQQLTDLQDPSHLTFFLAPKIGDY

>P37882|CCNB1_MESAU G2/mitotic-specific cyclin-B1 - Mesocricetus auratus (Golden hamster).

MALRVTRNTKLNTENKAKVSMAGAKAVPVTLAAASKPGLRPRTALGDIGNKVSEQAQARLPLKKELKTSVTGKVSAKIPPPKPLEKVPPVSEPEVELAETHEPEPVMDEKLSPEPILVDNPSPSPMETSGCAPAEEYLCQAFSDVILAVSDVDADDGADPNLCSEYVKDIYAYLRQLEEEQSVRPKYLLGREVTGNMRAILIDWLIQVQMKFRLLQETMYMTVSIIDRFMQDNCVPKKMLQLVGVTAMFIASKYEEMYPPEIGDFAFVTNNTYTKHQIRQMEMKILRVLNFSLGRPLPLHFLRRTSKIGEVDVEQHTLAKYLMELTLLDYDMVDFAPSQIAAGAFCLALKILDNGEWTPTLQHYLSYTEESLLPVMQHLAKNVVMVNHGLTKHMTIKNKYATSKHAKISTLAQLNCTLVQNLSKAVAKA

>Q8IEW8|Q8IEW8_9TRYP Cyclin 6 - Trypanosoma brucei.

MNPTALREVSNLSTGSGDVKQRLAQRRSANLESCRYPYSSSAVRHGSGSLLSLRSSLYSPVGTAGKESSVSPHADRLRENNPVYCSDVIKDITTMYMESEKEAIYGQVLPSPRYLTYQPEINEKMRMILVDWLIDVHLKFKLHPETLYLTVSIVDRYLSSVNTRRTTGRYIPRSKLQLVGITAILLAAKYEEIWPPEVKECVYICANTYTRDEVIRMEREMCTELSFRFTVPTPFPFIVRLLDVVEGLERQQQTQQLSHQLQQIQSQSQSQSQQTQMQASQSQPQSLSRSRSQSRSQPRSDEECTAYVVQLRHTAFFFLDHGMLDYKCLQFSPSQQAKAALFLALVTLHMKEQGSSYVLNNDIIWTRQLQYYSKAQVRDFKACARTMLDFVSYVSTTRYQSVRRKYSSSKYGEVAKLTMPSEVPDF

>P87049|CGP1_SCHPO G1/S-specific cyclin pas1 - Schizosaccharomyces pombe (Fission yeast).

MSKVNMRALLESFSTVILSIYPLAKSTNQQSHSHSLSLTQFLYETIRRSRVDYTTLLLALYYFIRFRDAASSKPTNYIPLLCGRRLFLVCLMAATKFLQDRSFSNRAWSRLSGLPVDKLLVLEYMFYQCIDYRLVVPKHIFARWSLLVGECCAHATARYDSTDPNVASFGSVAQYWVSLFSNVQSSLDDLFSIACLTKIAHRRMNANAALKNQATRKPSSSPQTTQDSSPILTMAPSTPVSVGSTPPSTPSVLPIAKQLAPMNVCKAHIQASNQSRTLTTASPPEQIPLMEPQVYVNPQVLPGRLSSLSKPVSLPPTPSSPKVGVYRPMTSKSNGGVAYCYNAQKLNNATGPVTFNMPFASVLPLAVSVSCDLGTASAYVASLPQPCSQKRHLEEDYSCLTEHSAKRRSYF

>Q9R1A0|CCNH_RAT Cyclin-H - Rattus norvegicus (Rat).

MYHNSSQKRHWTFASEEQLARLRADANRKFKCKAVANGKVLPNDPLFLEPHEEMTLCKYYEKRLLEFCSVFKPAMPRSVVGTACMYFKRFYLNNSVMEYHPRIIMLTCAFLACKVDEFNVSSPQFVGNLRESPLGQEKALEQILEYELLLIQQLNFHLIVHNPYRPFEGFLIDIKTRYPMLENPEILRKTADDFLSRIALTDAYLLYTPSQIALTAILSSASRAGITMESYLSESLMLKENRTCLSQLLDIMKSMRNLVKKYEPPRSEEVAILKQKLERCHSSDLALNMVTKKRKGYEDDDYVSKKPKQEEEEWTDDDLVDAL

>Q10653|CCNB1_CAEEL G2/mitotic-specific cyclin-B1 - Caenorhabditis elegans.

MLRATNNRRTSNNVEKDSLQMAKHGNGPLKPVNAQGLQTKREAREILALKPSNPAPVETAQKSQRINLQDAETKCLAMADDIYKYLVHHEKKYLLEECFMEGGEPTPKMRRILVDWLVQVHVRFHLTPETLHLTVFILDRMLQKKVTSKADLQLLGISAMFVASKFEEVYLPDIHDYEFITENTYSKKQILAMEQTILNSLNFDLSCPSSLVFLRCLSRILSENDASPIDNQAFCYTYNISKCLGELALLDSVMASTPRSHIASASMIIALEVHPVDGIEAENAVSVICKQLGASKKVIEDAVALLAEVSYKNFKQGKLVAIKNKYQSSKLAQVSNLMTDDVLEKINRMGQNAKVDASEME

>P37366|CCL1_YEAST Cyclin CCL1 - Saccharomyces cerevisiae (Baker's yeast).

MTDIQLNGKSTLDTPSATMSAKEKEAKLKSADENNKPPNYKRISDDDLYRHSSQYRMWSYTKDQLQEKRVDTNARAIAYIEENLLKFREAHNLTEEEIKVLEAKAIPLTMEEELDLVNFYAKKVQVIAQHLNLPTEVVATAISFFRRFFLENSVMQIDPKSIVHTTIFLACKSENYFISVDSFAQKAKSTRDSVLKFEFKLLESLKFSLLNHHPYKPLHGFFLDIQNVLYGKVDLNYMGQIYDRCKKRITAALLTDVVYFYTPPQITLATLLIEDEALVTRYLETKFPSREGSQESVPGNEKEEPQNDASTTEKNKEKSTESEEYSIDSAKLLTIIRECKSIIEDCKPPSTEEAKKIAAKNYYCQNPSTLIQKLKRKLNGEDTSSTVEKKQKT

>Q4QAE3|Q4QAE3_LEIMA Cyclin 11, putative - Leishmania major.

MASLGAEHKLECKSMVLTLHTLFDIPRDLVSLHSRRESVELVLKPTAKYYSENKRFCKLDLPEQHVAFSPSSIIFKHDRFVLDFESAPPPCGLSFARSSTPHGAASFANSMPSNFVDLLAANTTLTAPGVASSTGKMPLHFESDARAGGALPPKPRLAFGESVDEDQDVNLRSPGSLKLRTSTTTLSSAMPPNQIVLRYPTRSDLKVIVEVMVGARQRRHDSSAVRWITPSWADRSIPGTISTFAAADGEVMRHKLVNRFVGLTVGHLSCTMELPHAADDLREVPAFAPTAIADAAERLCNDYERLRRFHTTDTEEQLNAVRSERPARLRDFVEQLSTGASEFSIYRGLLLEVCPSFFVRRIRRICAMYPPIPQSSSWVRTDAETLAAEHTGREMSLIRNLCAELGPDLSAISPRNRFLSYTNVQPLGDELWKWIKDNYAHQENDILDYEPDMFMESLALHYGTPEPAPIQYGFSPISYELDRRAYFCDSLRFPVNREHHRYRDEAMAARQMRVPLQLTPCERPLRPIRPTSRSFGEIDILLEKYKPEFVQLVPSIADAMECVTQQNEHLLRELLRESQPAVGAAGANASTRERMHALPYEVCPHGFHLFQSERPSGSYVALLTRFAEYTYISVSTLLASVIYLDRLCLRHPRLLLTTRNIEKLLVAAVRVASKIVDLRSVNNKNFASVFSVPVQDMNELESEFLKLMNFDFFLSPKEFNNYAHLVQLPAAYMQMPSSVRLSNTVSANGTVESSAPPPSASHDSADHVADVGHSGNGAALRPESNTNSSNTNKMLLNTPAVATPAPPRAPSPPSQQQGRSSSGAYSVDTSVAAQRALLSSKPSSTADGSTATGVEAGGKYTNGSGGAAGHRYIRSGVRGGGSGGGVRGSVSANGYGSSSSAGSAGGGGSGGTSGSKAPSAEFASGTRVLSIP

>P24866|CG11_CANAL G1/S-specific cyclin CLN1 - Candida albicans (Yeast).

MTSLQQQQQQQRVKYGPPHHIKRRPYHPILESLEFQTNQHLIQEYSLDIVNTLSQLESLTLVNPAMIDLQPEIQWFMRPFLLDFLIELHSSFKLQPTTLFLCLNIIDRYCAKRIVFKRHYQLVGCTALWIASKYEDKKSRVPTLKELTIMCRNAYDEEMFVQMEMHILSTLDWSIGHPTLEDCLQLAIDSNNLSNNTTNDIENKSVRPNRKSSISSAVTAVARFLCELSLYDKYFLSVPPSLIAITANLLSCSMLQIPHASITLKNLIEQEIINPQQKKQKKAFSSNSSRTTTASYTHQNQSDVRHSSFDEDIDLDSGDEGDDDEDYIDEFYETNNYDDTNATTFDESINKSTTINDENQPPQIHTPFLSGLDEDSILSIKKICLMLIIQLSKVTEVLSKKYENLGVIQVINNFHSNYKFIIQSIYENQELLLNTINDSTNNNEIDYKLIQSSEILLQFPKFDEYLTEDEDENVSTDDEANSQPQGYDGSGSDGNNQLFTPKSPNAFSSNSSLTLNNHPQSMVPVTPPSATSQYSLFSNKNNRTHESTSGLNSTCNTPTHISISSFAPPQPPPGSILKPKLTSINSTNSLKIKKLTSNSNSSNINIHHGHHNTKQEKRYSHISIGSNSSSKYDGFSPIKSISTNGS

>O14332|REM1_SCHPO Meiosis-specific cyclin rem1 - Schizosaccharomyces pombe (Fission yeast).

MNSNNKRVALQEISNYVSKSLNVKGWVNAQVREISLQNGCSPSDKHFPSQSWHGISSIEESQIQKDYDSFLASPLEEEIVFTEKQLLVDLDVCSISNVQSPNCSVSEFGIAIEESNDNCAEEDEEEELQMRYSADESVSVEYAKEILSHMEKLEIRFMPDYRHMSAQPYYVTEMRASVINWIVGVHTCINLLPESLFLSINVLDRFLSLQNVPASKMKLCGATALFIACKYEEIHPPTVKDLEIVLEGEWIGEDICGMEKYMLMVLQYQLGWPGPVSFLRLLTIVNKWESQLRIMIKYFLEVSLVEQRFSSLRASQLVATCAYTGQSILQEENWSNTLPQITGYDYMSLVSYVHLLLKCLENPFDHHYAIYSKYATPYFHGISRQVHDWIATNTNLMAMDGS

>Q01043|CGH2_SHV21 Cyclin homolog - Saimiriine herpesvirus 2 (strain 11) (SaHV-2) (Herpesvirus saimiri).

MADSPNRLNRAKIDSTTMKDPRVLNNLKLRELLLPKFTSLWEIQTEVTVDNRTILLTWMHLLCESFELDKSVFPLSVSILDRYLCKKQGTKKTLQKIGAACVLIGSKIRTVKPMTVSKLTYLSCDCFTNLELINQEKDILEALKWDTEAVLATDFLIPLCNALKIPEDLWPQLYEAASTTICKALIQPNIALLSPGLICAGGLLTTIETDNTNCRPWTCYLEDLSSILNFSTNTVRTVKDQVSEAFSLYDLEIL

>P42753|CCD31_ARATH Cyclin-D3-1 - Arabidopsis thaliana (Mouse-ear cress).

MAIRKEEESREEQSNSFLLDALYCEEEKWDDEGEEVEENSSLSSSSSPFVVLQQDLFWEDEDLVTLFSKEEEQGLSCLDDVYLSTDRKEAVGWILRVNAHYGFSTLAAVLAITYLDKFICSYSLQRDKPWMLQLVSVACLSLAAKVEETQVPLLLDFQVEETKYVFEAKTIQRMELLILSTLEWKMHLITPISFVDHIIRRLGLKNNAHWDFLNKCHRLLLSVISDSRFVGYLPSVVAAATMMRIIEQVDPFDPLSYQTNLLGVLNLTKEKVKTCYDLILQLPVDRIGLQIQIQSSKKRKSHDSSSSLNSPSCVIDANPFNSDESSNDSWSASSCNPPTSSSSPQQQPPLKKMRGAEENEKKKPILHLPWAIVATP

>Q4Q5K6|Q4Q5K6_LEIMA Cyclin 9, putative - Leishmania major.

MSSSPLSPVYSSPASTGFGAPYLPSFTRRYFRLSQSAFVISDYLVRKFCLTESVLLTSLCYWHEFVAAHGLRKVNEVVLATTCVFLAAKVEHAHIRLARLVETALDLDANTTAAAELEQWCRAVLDVELVLCDTIRFDFVRIFPLADTLRSVQTLAEAGQLQDAARQEIGTAVRRVFLFSFVTPLCTKVSMQRLCTSILYMIVTAARREPVPAFQSIWSAPEAEFPDEAELEGITAVLMDVFAYLHKKFGVPALDDVNEARYKRRRSQHGNDVGVASSVFSSASADFTPLMKMSEQ

>P47829|CG21_CANAL G2/mitotic-specific cyclin CYB1 - Candida albicans (Yeast).

MPQVTKTNNENEFRLTRSKVQHQESISTIKNTTISNSQHKQQTQQQISSPPQVSVTSSEGVSHVNTRQYLGDVSNQYITNAKPTNKRKPLGGDNAPLQKQQHRPSRPIPIASDNNNNGSTSSSSNSSNNNNNDANRLASLAVPSRLPQKRQATESSTNLVEKLRVPQPEVGERSQSYHKKSRLIDYEWQDLDEEDNDDQLMVSEYVNEIFSYYYELETRMLPDPQYLFKQTLLKPRMRSILVDWLVEMHLKFKLLPESLFLAVNVMDRFMSVEVVQIDKLQLLATAALFTAAKNEEVFSPSVKNYAYFTDGSYTPEEVVQAEKYMLTILNFDLNYPNPMNFLRRISKADDYDVQSRTLGKYLLEITIVDYKFIGMRPSLCCASAMYLARLILGKLPVWNGNLIHYSGGYRISDMRECIELMFQYLIAPIEHDEFFKKYAMRKFMRASTLCRNWAKKFQASGRDLFDERLSTHRLTLEDDDEEEEIVVAEAEE

>Q4JAI6|PCNA1_SULAC DNA polymerase sliding clamp 1 - Sulfolobus acidocaldarius.

MRIVYDDVRDLKNIVETLTKFIDEGLFEIGQDGIRLVAVDKAHVSLINIELYKELFKEYEVEDEFKFGFNSQYLAKILSIAKRKEEISIESDSPERVKITLGGALNRVFIINNIQVSPPEVPEVNLEFEVKASLSSKAFKTTINEISAVTDTVDIIAVEDKVILKGEGKEGSQIENEFSKDTGAISDMEFKNEAKSPYDVNYLSDILSLTNLSDYTRLAFSTEKPLELEFNMEGGGKVTYLLAPKLS

>Q9YFT8|PCNA1_AERPE DNA polymerase sliding clamp A - Aeropyrum pernix.

MFRYEAKVFKELVDSVSKILDEGLFIITGEGLRLRGMDPARVALVDIEIPSSSFFDFYMAGDVERVELGVNMETLKGVVARAKKGDQLEVRVREDKVLFIVESVVLRRYLLPNLEVIVDVPEDISLEFDATATVIADVVKKTLRDVELVGDIVEFDAGEDYLSIRSVGPERRRVETRLTRESPALIDLEVKEPATSRYDVGYLKRMLGVAKIAESIELSFSTDKPLKMVFKSPDGSRVTYLLAPSTG

>Q06374|CG1_COLGL Putative G1/S-specific cyclin - Colletotrichum gloeosporioides (Anthracnose fungus) (Glomerella cingulata).

MLKRATVDVSSPADVPQPKRRKAYLTGADQARDEYAEDAIKHMEYIESETTPDKRCIPASVLEMRPEVIRSLIKIHKELNLLPQTLLLSVNLLDRFCCKKDFLGQVYYRVFSCAALWIASKFVEDKDRVPSVCRVLDYVPSGMYCNSALFFRLEICMLNVLAWRVNHPTPWCFIQLFCPGEGNKTKVRELALDICTARLPENRWMAIRPSILARHCLLHACNNLFSDTSLVEMYGSMPRDKPSTH

>Q585A1|Q585A1_9TRYP Cyclin 3 - Trypanosoma brucei.

MNIRTTVTLNSIPGPFGDPSSIDYKSHPGEGDTVSHVAIFEMMKKKEMTPLDDIKKLKKSVYSYRNRKIITNWLRDVCVALNLKGTTLCLAIQLTDAFISGSLQTLPIEKCQLAAATCLWDAAKFEEMDNSLPSLKKIVRVCDDAYSSEQILEMEETILCFFKWRLPHTTVINHLYLQLHMLGSEDLVSCGSVVKREPGEIVILNILVVEEDVHKWKRLEATEDCIITSIVPQLCAIAGIPASNNIEVFQVFGENVLVTRRTALDTPLRSLYVDSKRESRLCLSNGGVNFVFVERGTLVVPRNINKRFLNQCQVLVQEVVLHVEFIQLPSHVTALGVLMLSMCILATNMVEGKSAINYIQGKLGISSSRCLAAARLLCVKYRETIEEYRKSVKLPQLPEDIMERLDMCLATRNS

>Q979S2|PCNA_THEVO DNA polymerase sliding clamp - Thermoplasma volcanium.

MIRMNISVRNLKEITDLLSTIVSEAKFKVDENGMSVTAVDPAHVAMIRLEVPKEVFSEFRSDGTEEIALDIDRLKSVIRLANSSENVGITKDKEKLKFDLGNISKSVSLLDPSTIVTPKIPNIASEYYAIIKRSDFERGLRAAEDISDSIRFVLSSEGFRATSHSESEESEMVLPKDMLSDLSCSDTIKSSYPLEYLLKFIKAVSSADSLKISFRDDYPLSIEFYLDQNPSAKIKGLFLLAPRMEQ

>Q5JFD3|PCNA2_PYRKO DNA polymerase sliding clamp 2 - Pyrococcus kodakaraensis (Thermococcus kodakaraensis).

MTFEIVFDSAREFESLIATLEKFFDEAVFQVNMEGIQMRAIDPSRVVLVDLNLPEMLFSKYSVESEEAIAFDLKRFLKVLKLARSRDTLVLRKGGENFLEVGLLGDENTWFKLPLIDANTPEIEIPSLPWTVKAVVLAGALKRAVKAAKLVSDSIYFMATPEKLTFKAEGNDSEVRTVLTMEDPGLLDLEHKMTKAKSAYGVAYLEDILRSLADADEVIIRFGFDIPLLLKYMVRDAGEVSFLIAPRVEEGRS

>P48961|CCND3_RAT G1/S-specific cyclin-D3 - Rattus norvegicus (Rat).

MELLCCEGTRLAPRAGPDPRLLGDQRVLQSLLRLEERYVPRGSYFQCVQKEIKPHMRKMLAYWMLEVCEEQRCEEDVFPLAMNYLDRYLSCVPTRKAQLQLLGTVCLLLASKLRETTPLTIEKLCIYTDQAMAPWQLREWEVLVLGKLKWDLAAVIAHDFLALILHRLSLPSDRQALVKKHAQTFLALCATDYTFAMYPPSMIATGSIGAAVLGLGACSMSADELTELLAGITGTEVDCLRACQEQQIEAALRESLREAAQTAPSPVPKAPGGSSSQGPSQTSTPTDVTAIHL

>Q810T2|CCNB3_MOUSE G2/mitotic-specific cyclin-B3 - Mus musculus (Mouse).

MPPPLLPKRSKLETEKAQSNKITPREEQQSEKIGKSNHAASSSSSSTQGAVKRRSVFEDVTNASHSQCVQSKEDNIELKSHVSKRTKKGVGEVTQKKIKSSKMGHVTSLSNMEKEFILDIPNKPKTLTTEEPSVFQKTLVLNEEPATKETCLMRKTLKSCAFHQETLLMEKPLTLLVETEDYNEFDTELMTSKKKDKPEDPTIIEEMTDLKKSVIRKVTLTSSPLWLKNKHVVQEEKPVIQEKSSFKRISLVSNVVTTKEKPPVKKPHFRKKKPTTEMKSLLQEPSLEEKYNTQEDASILKKPQVLQENTNNKDATLTEPVTFKGKHSANEATHTKKPSSSKNNPDPQGKGTNLRPLRVHPVTYENEPMSSKKSTTKKKDSHFHGPSVLPDKHSPQMEVSTVKKSLALPNPTTEEKMLHFPVATVLEKQHNMGEAPCLKKPSPLRKQQQLPKRRRFFSNSAVQETVIRKPLFFKMSTTEKDPPSQWPSALPKKHISPGELSKQKKQHVSPKHNMEEDSQCWLDSAFKKQLSREEPASTHTPLKLEMQQAITKETGFHLRNPLVLPTVTSEAKSLTKEPPSFREQNTSLLKRKSTTHTITLQQAQSEWQEMTDEDRNLFSIKPGSHRKEPIPEFLQNPLPPNENCLISQKLSHSMPFASQKTTSQERAHRKESVASNDDKNFFSQDLFSPFSSADEDTLKFHKSLDFQEQVDRKNDSHKKMFDSQDSVSEEESFLRKLFCKDRCSSTEELSQERTVALEQEFLLIKILNENTSSDVDEPLSHQSPHIQNHSDTTKEALEASEALEAPEALETLEALVASEDLEEPLNILEELSTENMVALMKMLVTEDESTKDSFSGNYTAAREAHAEKSLSLEETSINEAATLKESLSSQEKHRAELVTVLKELLVLMKNPSLKRVALAFQENPSNNVETLLREVLALVENSTADESTLQEKPSTKTDVTPKELLALEENSSNKKANPMDSLSFDHKPDTEMGEIARMVLTDEEYNIDTLYERVLALSQGLIAADQLSFTDLQNFEETKIVDEEEFFKSFLVFENKNSPNMSSNAFESRTDNSSAIMPSSKAFNPVENSNPYVSSSKSFKSTLGAKETEITIQDDSDSLERIEKEGQDPLLNTIYAKDVFNYLKEREEKFLVQKYMDGQMELTSDMRAILVDWLVEIQGSFQMTHETLYLAVKIMDLYLMKAQCKKNHLQLLGSTTYMIAAKFEESYPPSLSEFLFICEDMYEKSDMVSLESSILQTLNFDINIPTAYNFLRRYASCIHASMKTLTLSRFICEMTLQEYEYIEERPSKLAAASFILALYMRNLSNCVPTLEYFTGYKMAELHILVRKLNHLLNFRSHSILKNVFEKYSEETYFEVAKIPPLSKQDLENLLNCALFH

>Q8HXN7|CCNT1_PANTR Cyclin-T1 - Pan troglodytes (Chimpanzee).

MEGERKNNKRWYFTREQLENSPSRRFGVDPDKELSYRQQAANLLQDMGQRLNVSQLTINTAIVYMHRFYMIQSFTQFPGNSVAPAALFLAAKVEEQPKKLEHVIKVAHTCLHPQESLPDTRSEAYLQQVQDLVILESIILQTLGFELTIDHPHTHVVKCTQLVRASKDLAQTSYFMATNSLHLTTFSLQYTPPVVACVCIHLACKWSNWEIPVSTDGKHWWEYVDATVTLELLDELTHEFLQILEKTPNRLKRIWNWRACEAAKKTKADDRGTDEKTSEQTILNMISQSSSDTTIAGLMSMSTSTTSAVPSLPVSEESSSNLTSVEMLPGKRWLSSQPSFKLEPTQGHRTSENLALTGVDHSLPQDGSNAFISQKQNSKSVPSAKVSLKEYRAKHAEELAAQKRQLENMEANVKSQYAYAAQNLLSHHDSHSSVILKMPIEGSENPERPFLEKADKTALKMRIPVAGGDKAASSKPEEIKMRIKVHAAADKHNSVEDSVTKSREHKEKHKTHPSNHHHHHNHHSHKHSHSQLPVGTGNKRPGDPKHSSQTSNLAHKTYSLSSSFSSSSSTRKRGPSEETGGAVFDHPAKIAKSTKSSSLNFSFPSLPTMAQMPGHSSDTSGLSFSQPSCKTRVPHSKLDKGPTGANGHNTTQTIDYQDTVNMLHSLLSAQGVQPTQPTAFEFVRPYSDYLNPRAGGISSRSGNTDKPRPPPLPSEPPPPLPPLPK

>Q4XHU6|Q4XHU6_PLACH Cyclin, putative - Plasmodium chabaudi.

MNYPENTSHIKKWFFKSRQEIDDICLKKYNDFKEKFKTYNVNIPKYADIEKTKLYFCYQLTHFCDIKRLQPQIVECAIVLYNRFYLKEIILEYDPRILIFTCIILAIKLEGYGRLYKINEFFSDIDINLDKVLEHENVVCSSLDFELNFLYTKECIFYLKNQFLNYINKYINKDNEVKNSFENICDKIYVNTSLECLKLLENFFITFTYTPAQIALYCFINNIKINFNIVNADKFILEFITNNNQILFQKLKNKIDELHIKYKDHFDLRNTFDDENTTKQIGETLDMCIDIYDILKKKKSHKKSKKNKLNNEDNEQNESKKMASIK

>P54733|CCNE_DROME G1/S-specific cyclin-E - Drosophila melanogaster (Fruit fly).

MGLNAKSVCSTSSTEPNGSIVTTAPSNGEVSSSIVVVVSSSSISSSSDSPIAILPHPDPIPSTSFSSASQRSEEELPGTSAASRTDEMCSCDSQNLAASTAATSNGNKRKRRLSSDSNEDPELGFEPPSAKRQQRLPALYGSEQGNLSSVASSVYTSPVVSVDGQSTQELLSIRSSPAEDLSEAPHSPLPDSPDSPPSPDRGSKQTPVVVRYAAEQVVTSTVVTQKTEDDDLLDDSCEDYSYDEDDEDDVEEEDDDVEIYSSTISPASSGCSQQQAVNGERTPGLPKHQEQIHHPVSDLMINMRTPMSPAVENGLRQCPLPALAWANAADVWRLMCHRDEQDSRLRSISMLEQHPGLQPRMRAILLDWLIEVCEVYKLHRETFYLAVDYLDRYLHVAHKVQKTHLQLIGITCLFVAAKVEEIYPPKIGEFAYVTDGACTERDILNHEKILLQALDWDISPITITGWLGVYMQLNVNNRTPASFSQIGRQKSAEADDAFIYPQFSGFEFVQTSQLLDLCTLDVGMANYSYSVLAAAAISHTFSREMALRCSGLDWQVIQPCARWMEPFFRVISQKAPYLQLNEQNEQVSNKFGLGLICPNIVTDDSHIIQTHTTTMDMYDEVLMAQDAAHAMRARIQASPATALRAPESLLTPPASSHKPDEYLGDEGDETGARSGISSTTTCCNTAASNKGGKSSSNNSVTSCSSRSNP

>E_tenella|Et_v1_Twnscn_Contig6778.tmp2|GeneDB

MLIAGTFSSKYKALFSSLHKAISWRANAFAGAFAGLQNRPYGVLAAPAAAAAAAAAAAAKMFECSLDGLTLRRIFEALKDTCAEVNFVCDAEGCCMQAMDDSHVALVSTHWGIDLFHKYRCDAQVTLGTAAAVLLLQCCCCCKAAAVQVLLLLLQCCCCNAAAAAAMLLLQCCCCCCNAAAAMLLLLLQCCCCNAAAAAAMLLLQCCCCCCNAAAAAACAGLSVPTLLKALQPVKSNKQIVHLSTLKGSDEDGEVLHIAIEDPERTGLARIHAAQQQQHQQQLRQQQQQQQEAQQQQLRQQQQEQQEQIRGEKDSLVLSAKGENLAVTRVIHAPDMQCSESFEQDFAVRYLSNFVKGASLCERLEVAMTQGTPMRFTFPLPPPGHCSAAAAAMAVDAEDLSCLRFYVAPKIDED

>T_congolense|congo611d12.q1k_8|GeneDB

MNIRTTVTLNSIPGPFGAPAAIDYKSHLGEGDTVSAATIFESMKKKEMTPLDDIKKLKKSVYSYKNRKIITNWLRDVCVALNLKSTTLCLAIQLTDAFISGSLKTLPVEKCQLAAATCLWDAAKFEEMDNSLPSLKKIVRVCDDAYTGDQILDMEETILGFFKWRLPHTTVINHLYFQLHMLGSEELVKCGSVVERAPGERVVLQLLVVEEDMHIWKRVESTAERTIKEMIPHFCAVVGLSVSNTVEVFQVFGENLLVTRRTPLDTALGSLNVDSRNESRLCISNGHLNFVFVERGTLIVPRTINKCFLNQCQVLVQEVVLHVEFLQLSSHVTALAVLTLSMCILANNMVEGTHAINYIQNTLGISAGRSVAAARLLCTKYAETIAEYCKSVNLPELPEDILERLDKCVPRKAG

>T_cruzi|Tc00.1047053508277.150|TIGR

MLEAQVQQANLWKRLIECISGLVNEANFDCNPGGLSIQAMDTSHVALVHLLLRDDCFTKYQCERNSILGLNLASLSKVLKIVEGSDSLTLHHEDDSDVVVLTSENVEKSRKCEYQLKLLEIEGEAMGIPEMEYGSTVTLSSQEFAKIVRDMSVFGETVTIEIRKEGVKFSSSGDVGEGYAFLRAAGGTGRTVRTAPEVKKEEDDDTPIGRANASGTKNSHTAIGVEVRTDEPVTLSFALRFMNVFAKGSTLSDRVSLKFAPDSPCMVEFNIDQVGYLRYFLAPKMDDVM

>D_discoideum|DDB0187046|GeneDB

MNYKSNSHWFFSKEQVLKHYSLGIELKLEVAYRRASAAFIQDVGIKLKMPQLTIATAISYFHRFFIRHQLKDHDRFVCINIDSPVVATACLFLAGKVEETPRKLDDVIKVSYMIKNKKKDGDKMVAISQQEHNNLKNKILQNEHLILTTIAFELAVEHPYKYLLEYMKSIQGSKNLCQVAWNFVNDSLRTSLCLHYPPDLISYASIYLATRFLNYQLITENKKEWWEMLGIKFEVLEDISKQILDLYEANPLQQTATIPSSSSSTPTSTSTTTSTSSPSPNPSQPQPLPPQQNENSNNSNNSNNNTTTTSTSTSSNTITTTSTSSSANEKQPSLNESGDINKTPNKSLSATTANSISHHISPTSSKPSNRNSDHRYSPYKSTHHSNSNKRSLSRSPSPNRASVSN

>D_discoideum|DDB0217508|GeneDB

MSSSGKTEKMSFFKKMGNNNNSGSSGISSGSSGSSSSSKKKSSSSSSSSSSSSSSSSTAIPTSITNIKSRSQSTDGLGNQSWIFNLKISTYKTIEAKVGSKVKLNIKNRQDAPLKKGDRFFFSISTLNKQRTDRIVSQSTVIHDPQPINDTTSKGETERTFDVLVQFDKIFLDPIPRTIFEPHPVLSDLAHSKGSFTKIPDSQLSLFLDIISNYSTKTVKKEAMNFLTGISLGENNSKKNDTNINSGNNSTPILNLNPSIHSTPLKEPISLDNRDDTINNNNNNFDNDNENNNLENIFERDYENNKLNIYDKNDGIILDQIAQQQAQQNLQQQQQQQQQQQNQQQQQQQQQQQQQQQQQQQQQQQQQQQQQQQQQQQQQQQQQQQQQQQQQQQQQQQQQQQQQQQQQQQQQQQQQQQQQQQNNFYSGSPNSLPPNLLNQIQQRSEQQQSQQQQQQLLKNQNMIPPQPHLQYQQPPPKHQMSQLQLQSQQELSKQKPTKKRNTIEEYNSNYNLSIQQKIVFVTPNGHPISVFSLIKGESRKTKLKTSKKDHHHHHHSGIKNKENLNINSLNNANIDNNNMIMGGNINCSGSDGQMLINGGIGVGGSGNSSGGGGGGGSGGTNFYLISIFPFIKKGAIKEELNEQFRQKHQWISQPGITLYKIRKIKRKLKKITIQSGLEISTLGLAYVLIEKLIMKNLIPRSNFKLVASTCLLLAAKFNDTKAMENALGPFFEQVEKRFDISKKEVLASEFLVFSQLSFTLFVSQFDVLPHLNRLKSEAVEDEELIF

>T_cruzi|Tc00.1047053508385.30|TIGR

MISCERSSILFTRAQRPLTASTTTTSSSSSSSSTTTTSVGMNSCLFSGHTAAASFCFSRGDSYHAGSGNANANGSCNNSNNNNNNNNNNNNNRVMYTSSAVAAGMAAASHFAPSRPPDGDGGGQGYSTGGHHAKNFAGVGTATTVAIENVEGGMVDTGAMGTEDNNGEADDEGMKRTSSSNDEPTPETCTDTTPRTVIDEVFAQPHEGQQQHHHHQEEEEQQQQQQEGDALQEQQQGFTFSQDTPPLSCNSREDACKFEVSNEDSKDVDVAGATEEAEACEMCAFSPVVVSARKPLQSQIDLVTARMAVTLFLENLSEDNKAEPVLTSDFHSHRLPQMPIEAYLDRVVRHSGVSGETLIASLMLLLKYSHFINHPVSVYNVHRLTITSLLLGAKLRDDQYYSNEYYSRIGGISNAEINKLELRFCGCLEWDMWLDESEYETLEKLLLRLVTVFASNTEEIDEVTRRRFQRQFWVEELRPWKERFDVSSSQRRERIRRCAAEDFSSEQAKIQHLSPSGSLFPQNPLVYPGTGMPGMSLDQVILPAVYRQTSGGTVSNAHTTTLVTVIAQNATGSTTNIPTHYYHSYHPIYQPPPRQQKQYGCGVYDTNINNINNNTNSNVGSIGSGRSTNAGAGFGILERAVSLSPTTPTTRTTTTGLHHSGISIHAKPYHYKSRGSSTKKFFNKPHRYHYASSQQMDSGHAGEIAMMEATTATYGSSSSRQNNIHNNNYNNNDTEFASITSTVWRPSGTNNNNNNNNSSGRRPATSDSVTMERHSGGGGGGGNAVRGSGPLVFPSQPQPCTDAANEEVAERRNRRKRPKPDHYRDYR

>D_discoideum|DDB0218655|GeneDB

MNNFNSYDSKIINNFNYDNKYIAILNIEKFQSDIKNFYIYHQKNVNNSEEQDWKRFNNCFKRMLVFMRKVCDSLSLSSLAYLHSISLLKRFLNRKFIFDYNQYFVSMTCILICIKVNSSSILPMKVRDLLNVSYYILTNDRLLLNDDYYNFKDNILSMEQKILQILSYDISVSFKENFAGIYNNNNNNNNNNNNNNNNNCVGKIIDEAPPYLFHFLYFLDCEKNHVLSQISFNFLSDSSLIPFIESPFFKAPEIALFCILLAFEYTKIDLPLSRNNLFLILGLKINNNNNNNNNNNNNNNNNNNNNNNNNNTDLNDQKYEIFKNYLHSLYIDFKNNGEIHYNNNGKEEEEYNYKLISDFFKKENKKD

>T_cruzi|Tc00.1047053511025.120|TIGR

MNSTTLREVSNLSGSIGDVKQRLAQRIGGGWGSYRLGSSTTVSRSSLRDFGSGATERAKEMLVSPRADRYHAEDPVYCNDLVKDITVMYMEKEKQAEGQLANDQVVVSPKYLTYQPEINEKMRMILVDWLIDVHLKFKLHSETMYLAVNILDRYLSCVNTKQSSGTYVARSQLQLVGITAILLAAKYEEIWPPEVKECVHISANTYTREEVIKMERNVCAALSFRLTVPTPFPFLVRLLSVMEGLVHSGSLSEDYTLQLPLLRHTALFFLEHGMLDYKCLQFKSSQQANASLFLALVTLRIKQKGGSCSFAGETIWTRQLQHYSRARVHEFKACAEAILEFVNYVPTTKYQAVRRKYSSAKYGEVAKLIMPNEVPDY

>D_discoideum|DDB0218006|GeneDB

MGNCCSSSKRSNGYDSTDDRILEESLDIKGGHNNNNHGGGHHHHHHHHHHHIKRNSNGGLYNNEMKSISSENNNNNNNNNNNNNNNNNNNNNKRNSLKSSSQHKLLVDEEKEDLISASVHQKDPYDLSTSSTLTNHSSTNTLVSSGVQTTSSSSTSSIDNLLDATTTTTTTTSTTNNISSQSKNTGSHSYPHTHTRQMSTEKVGPQFKTPITSIPHHMRMEGFQRVKHNSTSSLYIKSTLSTPDNDEILRCMANALLYHIERGSQFPQKTVEIFSEEKYPITKNKIDLKSNPTVETIYKFIRDIFKAEKLDSECAIMCLAYIERIITYTGITLSSINWRRIVLSALILASKVWEDQSVWNVDFLPVFDNLTAADLNCLERQFLAMLQYNVSLNASIYAKYYFELRNFSKLDSNQFPLKPLDKSGVRRLEDHSKASEYRVKPFKRSASVDQINPPEIHTRAVIN

>D_discoideum|DDB0169197|GeneDB

MSKVYSKSQTVSPGKPQNIFVTALPSSVSAPSLLSLQKPNNGAGSHRKQRQNSGSSKNRPNLPLDQIKSTERKNSGNKSNNNNNKNRKYSKNNSNVNSNNINSSSIVIKCEEQQQQQQQQQQQQQQQQQQQQQQQQDGFLKSQEFPNLFKNKLYLKDDISSPSSSSSTSSPSCLSSSSSSTFSQSEIAENKDDINNNNCNNTNGNFEEEEEEEEKNKVEQQSIEEETFIESSFEETKEVVEEVPFEEQFEILPISIGEETLKEYTSFLSVLSELIVDMYNDDDKSSTSSQSSSSSSSSSAPSSPKPIKNNDNNTTTTTTTTATTTTTSKTATSSLSTIEKFQVFNVDQIPEISIEAYIERVFKYLPFGTDIFIFSTIYLDRLIQWNQEIQISPLNIHRLFMASIIVASKFHNDKALNNRYYAQVGGISLFEMNQLEIHFLLLLNWKLHIDPEIFDAFKTSIQSKIDILDMKIRKLSNNGNINNESNKENIVIKTTEEKVKNSEIHINSNNNDCNNNNNNATVTTNPTPNPNPTITTTSTTTTTTTTTIPTPSTQSNNNNCNNSTKRYVTLVETDKNNSKPKTKYNSSNKSTNNGGTSQFSRILNNRGSKNVSYHHPNYLTYPLAAPQTNYQPQQQQQQTYYQPPHHQQQYYQQQQYYSNYQYNQQLSYYQQQQQHYDDYYQQQQIPIPPQPQNYIYQQPYHSTSSTSSTNIYIGKSGTPSSPNMNDICGGVSESYDSQIPSSFGSSSGSGSVGSCTSSSIGSTTAGSASQYQPHSWQNAYYYGGVATSQFSYHTPTYQ

>T_cruzi|Tc00.1047053509507.49|TIGR

MNLSSNGNNSLRTIDINLEFFSNGKGFYLPRPQQNLGGKTEQQMARQVASGRSDTSTQLNYDEVRRGLQNALSRVELLCGNSAEGRRQLLQHELLRLFLSASEETRERLEFVLSDAPLSYRVLEVLYLRRRELCTGFEYALSDFKCWLSLAMLLDEPDFIRFMMQYAQELGPCSRQRLEDVMFVHESSSKSLCHAVLGIRAPGEGAVPFDFRYYNFMLDDLIAMGDEPPHASLISSVQREVTLSMRELHAEWLLQVTMAVNLKLETFFLAVAIVDRYLAKVAVKRERLHLVGCAALLLASKQEEIFPASLHALVRYGADHFTVEMLVATECQLFAQVGFDVVLPTLSAVGMGILLQQDTAPSEAQRSCLLYVLATLAIRTHYRQYKLSSLAAAAVYMSRVCFDIPTGRPCEEVLSHLPLIRAALSRNPAVGSGGVH

>D_discoideum|DDB0189937|GeneDB

MSYNESSQIKYWMFNNEGLKKLREQCNNQHKQVILEKTPSSEPNILSPDDELSLIHYYETKTLEIAMALNLPDKVSAPAIIYIKRFYLKNSIMQYGAKLVMLSCLFIACKTEDNHLDIDYYSNITKASPSDITNLEIIILESLNFNLIVYHPFRPMYGYILDINDNSAIYNNTNGVSPIKFDTLWETCKKSIQKSLFSDCCFEFHPQIIALACLNLNWDGFNMYCINNNNNNNNNNNNNNNNNNNNNNNNNNNNNNNNNNNNNNNNNNNNNNNNNNNNNNNNNLLL

>T_congolense|congo364e01.q1k_7|GeneDB

MSGEVMRHTPAQNRMATIVHMYVCHLMAACRNEGSQETSKGRKAEFFVNLFPPSKFFFTETVPSISLLSYVQHIVAHVNCSPEAYIFALVYMKRLSAAGFPLETRSVYRIFLTAVVVAARVRDDFLRSKKSYSVIGGVTTRDLNAMEFRFLADLLEYGVEVSIDEYRALCNEITILESSGKVDGLEGSPNISGSASDEEPLNSPTPQWILDCCLHW

>E_tenella|Et_v1_Twnscn_Contig11490.tmp2|GeneDB

MATQIVLVPRKLQQRTPSIVDGLTREVEISQRIYGCQLIQKAGILLKLEAVTVASAQTILHRFYYRKSLKKFDVRLVATASLLLACKLEEDPRRVKSLIDVVHILSKAEDTNKDITLENLDELLLDHDSTVRCCCCCCCCCCCCCAVAAAVLLLLLLLPRRLDPEFELARSETFRCERYILRELGFMVSQTLVHPHRYILQYIHALCKGDYIPTNRLSQIAWGYLNDRVREEGLLVMVLVLLLMGEVLLLLVLLLLMGEHADDAVLRSAAGSGGCGEHLPGCMRLVDSAREGDRLVRTLRRELGRCLKVCTRILSLYKREPPRYAKLAESRTAAAAAAAAAAAAAADDELSGERNGRKGDAAAAEAAQQAADAAAEDAAAADAADPAQDAAAAADSAAAAADAAAEPAAAADDDMQVDYGESERTAQTFKKEKEKEKEKEKEKEKEKEKGRSSQEEERARPRRPQLEGAFIVEKREMRE

>D_discoideum|DDB0169229|GeneDB

MDSNRKRKSPTSITTIDKITKKKNNINNNNNNYNNILPSIGYLEMPLENDKLVELPPLHTPPDENLKFQEYKQPQEPQEPQPQELQPQEPQQQELKPEELKPQELKPEELKPEELKPEELKPEELKPEPPQQPKSIEDLANTNNFLFKITQLIKIFKNSDKKNPLYLKSWLYERNPNYSNINHFYPIINDSQQIDYSSYTHNSSKILNELKKLYIIVDENSVFDSTTISTNNNDNNDNDDNNGDINVNNDNNDNNNNNNNNNNNNNNNNNNNNNNNNNNNNNNNNNTLIRASTFRKSVDPTNVEQKRKKISQIKRMLKLKLLNYIRSKLSKKQNNNNNDDGNNNNNNNNNNFNNVNDNSITPLPIAKALNENEIEEIDSEETESEELMNLSNINEDDIIVEFTKDPLKLVMSLNYHFEVSSIPQHLIDHYSNLINKQYEYQDRKTYNTSKSPCASHCRTDVIDFLVDLTSTIRMNKRTLQMTISIYDDFMDYIVGDVKPEKFEEVDVMQLVAVSSLSIASKLEELEEYQPSIRELNYSTNNSYTHDGIVKTEMGILSILKYKVMYPTPSHFLDYLLLITIQPYDFFDATKDSQITFANQFKLINDQILNISYKNLKYRQYLPSIIACASIASTRTIMGIEPAWKPSLRKISNIPMREIYPLYIIFIKEYNTLYKKSIIPEFISKKFKNYQFLQI

>D_discoideum|DDB0215898|GeneDB

MNQQVLKRQQFQNQQDYFYQQQLNNQPHLNLTQQINNSFPSSCQYYNNNSYYNLQQVPDYTFSTLSTMPNNNSNFFIDSSNLQFTNSGNNYLQNSYPYLMENGSCTSSIEQTTTTTSTTTNQLTQQQQLYSNNSQYFEFNNSQQQQHEQQQQTENRNIKYDIENGTYSYTNNNMVSKTSSVYQQNFYNTNNPSLQMAQENPTYYKQPIQQQQQQQQQQQQQQQQQQQQQQQQQQQRPVQQKSQPIAQQKQQQQQAQQFVQQQQQKSIIETQKQDDFVEKQQNKYQKTSNIKEIEKITIVKEKNNKNEKVIEKDLIEKTLEYQQQMVELKSNDQEKETIVQQQKQHQIEKEKHEKLYKKSYLMKAGVNEMFPKAISLESWNFVGETTSRIYQTLNSRAEFHKKSSYCRVLLVKKIFSWLVCHDKEFIKNHKTVDLVIAISDIINMIISLIELHRAPSSSTLYLIIYSSDRFVERTGINHHQIFNLLLTSAIVNLKFWNESLYIQNKTIADIFSFSVKDLNTMERRFLYGLDYNLCVTPNELELFINSLKKQSISSLNFLK

>T_cruzi|Tc00.1047053507677.150|TIGR

MELSVSPDVQRSKGYVSHSFHNLKVPFTATLADENAWPEAVFAGGISHDWTSSTGNLPSGQPVESLAACGGGKPSNSMKCTLSGSTLTKWKSDSNVAAKLLGNADIMRAGVAKTTPVMPIEDVRRMNCCGGCQVHLGRSECHCHHQDQHHHHQQQQQQKPSVMSAGNPSNNEILNGHFQSARLSFFKKAFSGNRPQHHGGNPRHHQAHHYQQHHGSHHNPIHVAGPARRSHRHRGFGAAAVEVIRNRTLAGNSSPHHKTRSPANRGRKRHWKPCHVNEQEGHTEFLVPLPRSRLSLDGRQESSTPVDEEVKTKFTASGSELGSGVVGDNTPSAFRLSNIAPFYECAIQQLITICGRRTSNAPEAAHGTSCNENPENLQKPILSSCLASIRSFDMASSVNLKSFSAPPSKFSLSQHALFTRTLCSSVGGSMQHGSGMEETFCSLNDMDPTQNEGQQNEEKEIQSVAMASGDAPKLVSEEVKEQEASPTMVQQLIESIGVHVAYGDAAPMVLIGALVYISRITLQSPSEDVGVTNANWYRLVAIAILIATKMYVDGSRKWNERISKATGISLKEVQKLELDFLFLIDFALLIKEEEVETWAEWMESVARKRGMSTQLRAFIFGRNSCPPSTATTPLSSFAGEDAFSRSFSHIPSGNLPSTPVSLTPANMTPQINAHVSPGTMKSACLSQRLSMSFVLDLAEKHPRPTSHLHPSPSSFHACETPNQFFPSPMYASLDFHRTERLFSIVHAPEEPSTPVKLLEVSQLFNDAPNSIPFTPPEQETRSPIEFFKRSFLAEGACNSDTSSGKARCGMDVFQKSTGKNDARRLDFDTFDAFDEEQTPPHRPLNVLSPPDQLNIALPPRAEYNCFANFKKNNSNRDIPFNNGKTFTVTAPQLLCSFPQKREDKNVREHATRSSASGSAEKRTSRTKADTNEVTPHVKIKSKYDKLVHAISNVRELFSAKPLCVRDTPFNVLSVATDGSGTANIGERDMYNNNNNSKDVDDRYVAECDEDEDHDEFLDEFEDDRDNFFLRCRPVLTHSPPA
