# Supplementary material for: CyclinPred: A SVM-Based Method for Predicting Cyclin Protein Sequences
Source: PLoS One. 2008 Jul 2;3(7):e2605. doi: 10.1371/journal.pone.0002605 (PMC2435623; doi:10.1371/journal.pone.0002605)
Supplement: Dataset S2 — Non cyclin sequences (Negative data set) used for training the SVMs. (0.08 MB DOC) [file pone.0002605.s004.doc]

**Data set S2. Non cyclin sequences (Negative data set) used for training the SVMs.**

>gi42568854|ref|NP_178237.2| MEE65 (maternal effect embryo arrest 65); RNA polymerase II transcription factor/ cation:chloride symporter [Arabidopsis thaliana]

MVWCKHCAKNVPKIRPFDGGLACDLCGRILENFNFSTDVTFVKNAAGQVCNIVTSVGNSSSRDRRRRKAIDELRNLKDALGIGDERDDVVDMAAVFYEAAMDQNFTKGRRAELVQSSCLYLACRDMKISLLFIDFSSYLRVSVYELGSVYLQLCEMLYLVQNKNYEELVDPSIFIPRFTNSLLKGAHAKAKDVANTAKNIISSMKRDWIQTGRKPSGICGAAIYMAALSHGIMYSRADIAKVVHMCEATITKRLNEFANTEAGSLTVDELDESEEILRKETFTPRPNSDKGVVNCKHKDLKRFGYGLCKSCHDDFIIISGGVVGGSDPPAYQRAEKERMEKAAREENEGGIGNLNHDEQVNVSKRAKKCSEKGEGETYGGERHAEYSDESDICSDDDDSEVEHVLLGEDETRLKTTAWNLQNKDYLEEQAEKEAALKAANCPEDARNLVEASKAAVANSRKEKRRKRAEEAKNAPPSATATEASYTETQRVNQHHVLDELLDTSPTQKKPRTETVTEKKKEEHEIVEDEEDEEDYAAPHADENFYEDEVEEEENGYDFGLY

>gi55819414|ref|YP_142898.1| transcription initiation factor TFIIB [Acanthamoeba polyphaga mimivirus]

MRMLIFTYKLERYIKNKILPKILVVPDRDKYQIKGSFRRRIPYITDIDIVNNVHPEYDDTNIYQRIVDLINSFTNDNQIKLIYVICGTDDRFLLTEYSDEEIEKIKILLNPTELVELNNVLSKYQDDLNKKVFYINEIIWDLYKLRWTSSEVLAGKKILRGGIEVSFQDVVKNNSILLLQYFVKIEYYPIGFDIAVRYKPINLITAYQNAAFYQLKLANYSKEYYFMLFPLRFYFKNDPTISKQLEYIIETKFGLYKQLLVRIDSYRTIYESGNLDLDTAKSIIISIIKDIRKLNGIDMNIIDKIQEVSNNSAGQDKIIAWNTLLTQLYTNINKSVNKQSKKYFTRYINIIPKEDRKLCCLEEEHVLQSGGINFESTNFLTKKKLIY

>gi4506435|ref|NP_000312.1| retinoblastoma 1 [Homo sapiens]

MPPKTPRKTAATAAAAAAEPPAPPPPPPPEEDPEQDSGPEDLPLVRLEFEETEEPDFTALCQKLKIPDHVRERAWLTWEKVSSVDGVLGGYIQKKKELWGICIFIARVDLDEMSFTLLSYRKTYEISVHKFFNLLKEIDTSTKVDNAMSRLLKKYDVLFALFSKLERTCELIYLTQPSSSISTEINSALVLKVSWITFLLAKGEVLQMEDDLVISFQLMLCVLDYFIKLSPPMLLKEPYKTAVIPINGSPRTPRRGQNRSARIAKQLENDTRIIEVLCKEHECNIDEVKNVYFKNFIPFMNSLGLVTSNGLPEVENLSKRYEEIYLKNKDLDRRLFLDHDKTLQTDSIDSFETQRTPRKSNLDEEVNIIPPHTPVRTVMNTIQQLMMILNSASDQPSENLISYFNNCTVNPKESILKRVKDIGYIFKEKFAKAVGQGCVEIGSQRYKLGVRLYYRVMESMLKSEEERLSIQNFSKLLNDNIFHMSLLACALEVVMATYSRSTSQNLDSGTDLSFPWILNVLNLKAFDFYKVIESFIKAEGNLTREMIKHLERCEHRIMESLAWLSDSPLFDLIKQSKDREGPTDHLESACPLNLPLQNNHTAADMYLSPVRSPKKKGSTTRVNSTANAETQATSAFQTQKPLKSTSLSLFYKKVYRLAYLRLNTLCERLLSEHPELEHIIWTLFQHTLQNEYELMRDRHLDQIMMCSMYGICKVKNIDLKFKIIVTAYKDLPHAVQETFKRVLIKEEEYDSIIVFYNSVFMQRLKTNILQYASTRPPTLSPIPHIPRSPYKFPSSPLRIPGGNIYISPLKSPYKISEGLPTPTKMTPRSRILVSIGESFGTSEKFQKINQMVCNSDRVLKRSAEGSNPPKPLKKLRFDIEGSDEADGSKHLPGESKFQQKLAEMTSTRTRMQKQKMNDSMDTSNKEEK

>gi55819128|ref|YP_142604.1| putative transcription initiation factor IIB [Acanthamoeba polyphaga mimivirus]

MSHHSETILTAILNTKYLENGSKHKIIELPKTNIKKLIKDFLSVNSNKKIQSDCVLINYFPNALLLDNPNKNSSVKKEQTNQVKIIEKPITNNINKPEDIWDIIDNMDKENNSESLENIQSENSENNDNFTDNNTKKSPTKSRICSGCGSKGTLLEDQSSSVLVCSECGMINDDLLDHGPEWRQYYNDDGRGEGVNRCGCPSNFFFPKSSQGTILAGTGSGRLKRKQKWNSTVYKERSLNDVFEKISTICSKSNIPRIIADTAKILYKKLSDCKHKSGNNVGKQIIIRGHNRISIIAACIYKACEMNKNPRTVKEIARFFGIDEKKVTKGNKQFEKIMKNTDDNMIILDPVNSNSTEDYIRRHCPRLKVNKDHTDIAVKISNNCCRMKLASDHNPQSIAAGAILVMVVFCELNIDKRKISRLFGISDVTIDKIYKKIAPYAPALVDDGATDHLINKLKING

>gi10047086|ref|NP_061821.1| mitogen-inducible gene 6 protein [Homo sapiens]

MSIAGVAAQEIRVPLKTGFLHNGRAMGNMRKTYWSSRSEFKNNFLNIDPITMAYSLNSSAQERLIPLGHASKSAPMNGHCFAENGPSQKSSLPPLLIPPSENLGPHEEDQVVCGFKKLTVNGVCASTPPLTPIKNSPSLFPCAPLCERGSRPLPPLPISEALSLDDTDCEVEFLTSSDTDFLLEDSTLSDFKYDVPGRRSFRGCGQINYAYFDTPAVSAADLSYVSDQNGGVPDPNPPPPQTHRRLRRSHSGPAGSFNKPAIRISNCCIHRASPNSDEDKPEVPPRVPIPPRPVKPDYRRWSAEVTSSTYSDEDRPPKVPPREPLSPSNSRTPSPKSLPSYLNGVMPPTQSFAPDPKYVSSKALQRQNSEGSASKVPCILPIIENGKKVSSTHYYLLPERPPYLDKYEKFFREAEETNGGAQIQPLPADCGISSATEKPDSKTKMDLGGHVKRKHLSYVVSP

>gi27363484|ref|NP_758961.1| E74-like factor 1 (ets domain transcription factor) [Homo sapiens]

MAAVVQQNDLVFEFASNVMEDERQLGDPAIFPAVIVEHVPGADILNSYAGLACVEEPNDMITESSLDVAEEEIIDDDDDDITLTVEASCHDGDETIETIEAAEALLNMDSPGPMLDEKRINNNIFSSPEDDMVVAPVTHVSVTLDGIPEVMETQQVQEKYADSPGASSPEQPKRKKGRKTKPPRPDSPATTPNISVKKKNKDGKGNTIYLWEFLLALLQDKATCPKYIKWTQREKGIFKLVDSKAVSRLWGKHKNKPDMNYETMGRALRYYYQRGILAKVEGQRLVYQFKEMPKDLIYINDEDPSSSIESSDPSLSSSATSNRNQTSRSRVSSSPGVKGGATTVLKPGNSKAAKPKDPVEVAQPSEVLRTVQPTQSPYPTQLFRTVHVVQPVQAVPEGEAARTSTMQDETLNSSVQSIRTIQAPTQVPVVVSPRNQQLHTVTLQTVPLTTVIASTDPSAGTGSQKFILQAIPSSQPMTVLKENVMLQSQKAGSPPSIVLGPAQVQQVLTSNVQTICNGTVSVASSPSFSATAPVVTFSPRSSQLVAHPPGTVITSVIKTQETKTLTQEVEKKESEDHLKENTEKTEQQPQPYVMVVSSSNGFTSQVAMKQNELLEPNSF

>gi22779868|ref|NP_683727.1| FYVE and coiled-coil domain containing 1 [Mus musculus]

MASSSTETQLQRIIRDLQDAATELSHEFKEGGEPITDDSTSLHKFSYKLEYLLQFDQKEKASLLGSKKDYWDYFCACLAKVKGANDGIRFVRSISELRTSLGKGRAFIRYSLVHQRLADTLQQCFMNTKVTSDWYYARSPFLKPKLSSDIVGQLYELTEVQFDLAPRGYDLDAAWPTFARRTLATSTSAYMWKPPSRSSSMSSLVSNYLQTQEMASSLDLNCSLNNEALESFDEMRLELDQLEVREKQLQERVQQLDRENQALRMLVSRQGGQLQVEKEMGYLAVEDSIGLVSLVAELQKQGDVSQATVKKLQSCLQALELNVDKKEYSPSALQLENMAKELDTVRGSLGRENQLLASLSERLARAEKGEKTPPDTELHQEPVPADLVLKFQELKGKLQALEGENTEAQELNRQQSIKLEQLAKELQLKEEARASLAHLVKDVVPLQEELSGKKQESAQLRRQLQESLAHLSSVEEELAEARQQEKQHREEKQLLEQEATSLTWQLQLLETQLGQVSQLVSDLEEQKKQLMQERDHLSQRVGTLEQLAEVHGPPQSAEMPEKRQQCLREEQVNNSTVSEAEQEELQKELQNMVDRNQLLEGKLQALQTDYKALQQREAAIQGSLASLEAEQASIRHLGNQMEASLLAVKKAKETMKAQVAEKEAALQSKESECQRLQEEADQCRLQAEAQAQELRALENQCQQQIQLIEVLSAEKGQQGLSLPQVNTDQLALSQAQLEIHQGEAQRLQNEVVDLQAKLQVALGDRDKLQSQLGVAETVLREHKTLVQQLKEQNEALNRAHVQELLQCSEREGILQEESIYKAQKQEQELRALQAELSQVRCSSEGAHLEHAELQDQLHRANTDTAELGIQVCALTAEKDRMEEALASLAQELQDSKEAALQERKGLELQVMQLQQEKEKLQEKVKAAEEAASSFSGLQAQLAQAEQLAQSLQETAHQEQDALKFQLSAEIMDHQNRLKTANEECGHLRAQLEEQGQQLQMTKEAVQELEITKAAMEEKLNCTSSHLAECQATLLRKDEESTMLQTSLERTQKELEKATSKIQEYYNKLCQEVTNRERNDQKMLADLDDLNRTKKYLEERLIELLRDKDALWQKSDALEFQQKLSAEEKCLGDMEVNHCHDCKREFSWIVRRHHCRICGRIFCYYCCNNYVVTKPSGKKERCCRACFQKFGEGSGSNDSSGSGTSQGEPSPMVSPAEASPQSIGSQGINSVCRPPDDAVFDIITDEELCQIQESGSSLPETPTETDSMDPNTAEQDTTSNSLTPEDTEDVPMGQDAEICLLKSGELMIKLPLTVEEVASFGEGSRELFVRSSTYSLITITVAEPGLTISWVFSSDPKSISFSVVFQETEDTPLDQCKVLIPTTRCNSHKENIRGQLKVRIPGIYLLIFDNTFSRFISKKVLYHLTVDRPVIYDGSDFP

>gi10047100|ref|NP_057387.1| WW domain binding protein 5 [Homo sapiens]

MKSCQKMEGKPENESEPKHEEEPKPEEKPEEEEKLEEEAKAKGTFRERLIQSLQEFKEDIHNRHLSNEDMFREVDEIDEIRRVRNKLIVMRWKVNRNHPYPYLM

>gi71754449|ref|XP_828139.1| transcription factor [Trypanosoma brucei TREU927]

MSSCSHPTSAQYVDRARGTITCTLCGDIVQDPQLELDPIFARGDKGGRLRALGHLRPTRGSVSVRMPSARPSIEAARRGMATIARQLDVSDDMVEAALGLYKLAVSLNAVSGARPAILCAVLYAMCRRERTSHMVFDFADATGESPYDILSYMHLVCEATRTEVPVIDPSCVVHRFAEQMNLGQMTRSVVVCALKVLRAMHDDWIACGRRPLGVCVAALLVACYMFNIPRSPDEVCGFVRLTAGTISRRLDEFAATSTAALDSIDKYTRDDSSLPPAFTSATKKYDGDKRDAELRQLSAMYYELVAEAKVSTPSTPDRCEKWRHFLLCHCALEGKTPSDASLDLTTLTPQQQLQILGLPNTKPIDSSKARASVKEEEDKIMVKLERVKQEPVRQEPSTPEKGAPPVPNAGMSLSEMTDYYRMLMTRDPNVLDIRRDFDMEAVNPDDVVPAPQLPEEEAVVKTEGAAEAKFKIDPCLAEVLYDNERTLALPWEFIVLQDPELDDMTDLEPYLVLDNEERLRRQKVGEALYGESWNLGAARTKEEIERLEESHSTRKRRREPIREHLTVQDAVSQALRRRGASTINVSQIDELIPGLAGLENSAEDEWVA

>gi51704823|ref|XP_484829.1| PREDICTED: similar to CDNA sequence BC016548 [Mus musculus]

MEELGGRTESPVQDRNSTGRPADSTNMDPWELSKTEPPTKEHTVWTEVPSPTPPYVADEQLSLHIDPQQQEQRLSLKLSPDYEIHSPIRFPYLALDKDVPNCEEN

>gi22035556|ref|NP_001510.2| transcription initiation factor IIIB isoform 1 [Homo sapiens]

MTGRVCRGCGGTDIELDAARGDAVCTACGSVLEDNIIVSEVQFVESSGGGSSAVGQFVSLDGAGKTPTLGGGFHVNLGKESRAQTLQNGRRHIHHLGNQLQLNQHCLDTAFNFFKMAVSRHLTRGRKMAHVIAACLYLVCRTEGTPHMLLDLSDLLQVNVYVLGKTFLLLARELCINAPAIDPCLYIPRFAHLLEFGEKNHEVSMTALRLLQRMKRDWMHTGRRPSGLCGAALLVAARMHDFRRTVKEVISVVKVCESTLRKRLTEFEDTPTSQLTIDEFMKIDLEEECDPPSYTAGQRKLRMKQLEQVLSKKLEEVEGEISSYQDAIEIELENSRPKAKGGLASLAKDGSTEDTASSLCGEEDTEDEELEAAASHLNKDLYRELLGGAPGSSEAAGSPEWGGRPPALGSLLDPLPTAASLGISDSIRECISSQSSDPKDASGDGELDLSGIDDLEIDRYILNESEARVKAELWMRENAEYLREQREKEARIAKEKELGIYKEHKPKKSCKRREPIQASTAREAIEKMLEQKKISSKINYSVLRGLSSAGGGSPHREDAQPEHSASARKLSRRRTPASRSGADPVTSVGKRLRPLVSTQPAKKVATGEALLPSSPTLGAEPARPQAVLVESGPVSYHADEEADEEEPDEEDGEPCVSALQMMGSNDYGCDGDEDDGY

>gi4503433|ref|NP_001940.1| E2F transcription factor 3 [Homo sapiens]

MRKGIQPALEQYLVTAGGGEGAAVVAAAAAASMDKRALLASPGFAAAAAAAAAPGAYIQILTTNTSTTSCSSSLQSGAVAAGPLLPSAPGAEQTAGSLLYTTPHGPSSRAGLLQQPPALGRGGSGGGGGPPAKRRLELGESGHQYLSDGLKTPKGKGRAALRSPDSPKTPKSPSEKTRYDTSLGLLTKKFIQLLSQSPDGVLDLNKAAEVLKVQKRRIYDITNVLEGIHLIKKKSKNNVQWMGCSLSEDGGMLAQCQGLSKEVTELSQEEKKLDELIQSCTLDLKLLTEDSENQRLAYVTYQDIRKISGLKDQTVIVVKAPPETRLEVPDSIESLQIHLASTQGPIEVYLCPEETETHSPMKTNNQDHNGNIPKPASKDLASTNSGHSDCSVSMGNLSPLASPANLLQQTEDQIPSNLEGPFVNLLPPLLQEDYLLSLGEEEGISDLFDAYDLEKLPLVEDFMCS

>gi13812036|ref|NP_113167.1| transcription initiation factor IIB [Guillardia theta]

MNTCINCGSKRFLEDYKQGDIICKNCGFIIESHIIDFGSEWRIFSDDNRSNNPVRIGLPENPLLGNSSSTLISKGLKGSNKINEKLLKAQNQNDNCKSEKYLASVFSIISFFLTNGSFSKLIKEKVQELFKNYYDYLTLKSNGSRIKTTLRKKDTFSIIAASIFIICKNESIPRSFKEISELTKVKKKDIGNRVRIMEKALEGIKISKKRDSDNFISRFCSKLGLSSTSSKIAEQIANFIKDKEGMYGRNYISVAAASIYVVSQIPNLSNNCNLKKIIEATGVSEITLRSAYKAMYPYRKEILLKIKNKESLICNSVFSNLTITN

>gi10092586|ref|NP_031371.1| zinc finger protein 236 [Homo sapiens]

MGLCGLLERCWLHHDPDGVLTLNAENTNYAYQVPNFHKCEICLLSFPKESQFQRHMRDHERNDKPHRCDQCPQTFNVEFNLTLHKCTHSGEDPTCPVCNKKFSRVASLKAHIMLHEKEENLICSECGDEFTLQSQLAVHMEEHRQELAGTRQHACKACKKEFETSSELKEHMKTHYKIRVSSTRSYNRNIDRSGFTYSCPHCGKTFQKPSQLTRHIRIHTGERPFKCSECGKAFNQKGALQTHMIKHTGEKPHACAFCPAAFSQKGNLQSHVQRVHSEVKNGPTYNCTECSCVFKSLGSLNTHISKMHMGGPQNSTSSTETAHVLTATLFQTLPLQQTEAQATSASSQPSSQAVSDVIQQLLELSEPAPVESGQSPQPGQQLSITVGINQDILQQALENSGLSSIPAAAHPNDSCHAKTSAPHAQNPDVSSVSNEQTDPTDAEQEKEQESPEKLDKKEKKMIKKKSPFLPGSIREENGVRWHVCPYCAKEFRKPSDLVRHIRIHTHEKPFKCPQCFRAFAVKSTLTAHIKTHTGIKAFKCQYCMKSFSTSGSLKVHIRLHTGVRPFACPHCDKKFRTSGHRKTHIASHFKHTELRKMRHQRKPAKVRVGKTNVPVPDIPLQEPILITDLGLIQPIPKNQFFQSYFNNNFVNEADRPYKCFYCHRAYKKSCHLKQHIRSHTGEKPFKCSQCGRGFVSAGVLKAHIRTHTGLKSFKCLICNGAFTTGGSLRRHMGIHNDLRPYMCPYCQKTFKTSLNCKKHMKTHRYELAQQLQQHQQAASIDDSTVDQQSMQASTQMQVEIESDELPQTAEVVAANPEAMLDLEPQHVVGTEEAGLGQQLADQPLEADEDGFVAPQDPLRGHVDQFEEQSPAQQSFEPAGLPQGFTVTDTYHQQPQFPPVQQLQDSSTLESQALSTSFHQQSLLQAPSSDGMNVTTRLIQESSQEELDLQAQGSQFLEDNEDQSRRSYRCDYCNKGFKKSSHLKQHVRSHTGEKPYKCKLCGRGFVSSGVLKSHEKTHTGVKAFSCSVCNASFTTNGSLTRHMATHMSMKPYKCPFCEEGFRTTVHCKKHMKRHQTVPSAVSATGETEGGDICMEEEEEHSDRNASRKSRPEVITFTEEETAQLAKIRPQESATVSEKVLVQSAAEKDRISELRDKQAELQDEPKHANCCTYCPKSFKKPSDLVRHVRIHTGEKPYKCDECGKSFTVKSTLDCHVKTHTGQKLFSCHVCSNAFSTKGSLKVHMRLHTGAKPFKCPHCELRFRTSGRRKTHMQFHYKPDPKKARKPMTRSSSEGLQPVNLLNSSSTDPNVFIMNNSVLTGQFDQNLLQPGLVGQAILPASVSAGGDLTVSLTDGSLATLEGIQLQLAANLVGPNVQISGIDAASINNITLQIDPSILQQTLQQGNLLAQQLTGEPGLAPQNSSLQTSDSTVPASVVIQPISGLSLQPTVTSANLTIGPLSEQDSVLTTNSSGTQDLTQVMTSQGLVSPSGGPHEITLTINNSSLSQVLAQAAGPTATSSSGSPQEITLTISGSRSVQHSVGPQECGSVEALYLENSSDKT

>gi68492125|ref|XP_710164.1| transcription factor TFIIB [Candida albicans SC5314]

MSNSNINNKKFTGPNLNVTLICPDCKQFPPDLIERFSEGDIICGQCGLVLSDRIVDTRSEWRTFQNDDKNTDDPSRVGDASNSLYDTEDLTTMISYAPNSNSGSSKNSLNKIQQKSLVDKKNYALQTAYQKISELCNGYQLPKSASDAAKHLYKAVREDRLLKGKTQDSIMAATILLGCRRADVPRTLEEIRALTNVPTKQIAKVTGLIKKIIRSHLNNDDASTSIVDETSSLTTHSAEDLIRRFCSHLGLSLAITSASEYVARKCHDLGVLAGRSPTTIAATSIFLAAKSLGDTKLTQQQIRDKTGVSIGTIKNSYKILQENAKDLIDPAWEANSKVKRENTPKS

>gi33239306|ref|NP_667071.1| olfactory receptor 161 [Mus musculus]

MGGTNQSSVSEFLLLGLSRQPQQQQQLIFLLFLIMYLATVLGNLLIILAISTDSRLHTPMYFFLSNLSFVDVCFSSTTVPKVLAIHILRNQAISFSGCLTQLYFLCVFADMDNFLLAVMAYDRFVAICHPLHYTTKMTHQLCAFLVVGSWMVASLNALLHTLLVAQLYFCGDNVIPHFFCEVTPLLKLSCSDTHLNELMILAVAGLIMLAPFVCILLSYILIACAILKISSTGRWKAFSTCGSHLAVVCLFYGTIISLYFNPSSSHSAGRDMAAAMMYTVVTPMMKPFIYSLRNRDMKGALRKVLTMRFISTQ

>gi15668963|ref|NP_247767.1| transcription initiation factor IIB (TFIIB) [Methanocaldococcus jannaschii DSM 2661]

MVWLMEALKTKENETTKEKKLTTKVEKSEKKEENVREEEIVCPICGSKEVVKDYERAEIVCAKCGCVIKEKLFDIGPEWRAFDHEQKIKRCRVGAPMTYSVDYNEPIIIKENGEIKVVKIGELIDKIIENSENIRREGILEIAKCKGIEVIAFNSNYKFKFMPVSEVSRHPVSEMFEIVVEGNKKVRVTRSHSVFTIRDNEVVPIRVDELKVGDILVLAKELPNIEEDIEIDKKFSKILGYIIAEGYYDDKKIVLSYDYNEKEFINETIDYFKSLNSDITIYSKDLNIQIEVKNKKIINLLKKLRVKNKRIPSIIFKSPYEIKKSFIDGIFNGKDAKVFVSKELAEDVIFLLLQIKENATINKKSINDIEVYEVRRITNIYTNRKLEKLINSDFIFLKIKEINKVEPTSGYAYDLTVPNAENFVAGFGGFVLHNTIHDKGLSTVIDWRNKDSYGKDLSANKRAQLYRLRKWQRRIRVSDAAERNLAFALSELDRITSKLGLPRHVRENAAIIYRGAVEKGLIRGRSIEGVVAAAIYAACRRCRVPRTLDEIAEASRVDRKEIGRTYRFLARELNIKLTPTNPIDYVPRFASELGLPGEVESKAIQILQQAAEKGLTSGRGPTGVAAAAIYIASVLLGCRRTQREVAEVAGVTEVTIRNRYKELTEHLDIDVTL

>gi30424964|ref|NP_780503.1| nuclear casein kinase and cyclin-dependent kinase substrate 1 [Mus musculus]

MSRPVRNRKVVDYSQFQESDDADEDYGRDSGPPAKKIRSSPREAKNKRRSGKNSQEDSEDSEEKDVKTKKDDSHSAEDSEDEKDDHKNVRQQRQAASKAASKQREMLLEDVGSEEEPEEDDEAPFQENSGSDEDFLMEDDDDSDYGSSKKKNQKMVKKSQPERKEKEMPQPRVKATVTPSPVKGQAKVGRPTASKKSKEKTPSPQEEDEEAESPPEKKSGDEGSEDEASSGED

>gi62751583|ref|NP_001015582.1| BRF2, subunit of RNA polymerase III transcription initiation factor, BRF1-like [Bos taurus]

MPGRGRCPDCGSAELVEDSHYSQNQLVCSDCGCVVTEGVLTTTFSDEGNLREVTYSRSTGENEQVSRSQQRGLRRVRDLCRVLQLPPTFEDTAVAYYQQAHQLAGIRTARLQKKEVLAGCCVLITCRQRNWPLTMGTICTLLYADLDVFSGTYMQIVKLLGLDVPSLCLVDLVKTYCSSFKLFEASPSVPAKYVEDKEKMLSRTLQLVELADETWLVTGRHPLPVITAATFLAWQSLRPSDRLTCSLARFCKLANVDLPYPASSRLQELLAVLLRMAEQLAWLQVLKLDKRSVVKHIGDLLQHRHMLVRKAFRDGTAEMDAGEKELQGQGQGQALGDEDVGSSSLELPAGKRPSSPALLLPPCMLKPPKRVCPAPPVSMVTGDEDISDSEIEQYLRTPQEVRDFQKAQAARQAAQGTPNPP

>gi21361411|ref|NP_036389.2| HMG-box transcription factor 1 [Homo sapiens]

MVWEVKTNQMPNAVQKLLLVMDKRASGMNDSLELLQCNENLPSSPGYNSCDEHMELDDLPELQAVQSDPTQSGMYQLSSDVSHQEYPRSSWNQNTSDIPETTYRENEVDWLTELANIATSPQSPLMQCSFYNRSSPVHIIATSKSLHSYARPPPVSSSSKSEPAFPHHHWKEETPVRHERANSESESGIFCMSSLSDDDDLGWCNSWPSTVWHCFLKGTRLCFHKGSNKEWQDVEDFARAEGCDNEEDLQMGIHKGYGSDGLKLLSHEESVSFGESVLKLTFDPGTVEDGLLTVECKLDHPFYVKNKGWSSFYPSLTVVQHGIPCCEVHIGDVCLPPGHPDAINFDDSGVFDTFKSYDFTPMDSSAVYVLSSMARQRRASLSCGGPGGQDFARSGFSKNCGSPGSSQLSSNSLYAKAVKNHSSGTVSATSPNKCKRPMNAFMLFAKKYRVEYTQMYPGKDNRAISVILGDRWKKMKNEERRMYTLEAKALAEEQKRLNPDCWKRKRTNSGSQQH

>gi15230282|ref|NP_191296.1| transcription factor IIB (TFIIB) family protein [Arabidopsis thaliana]

MTMKWGHSCRRCKQINVVTDHVTRRTRCFGCGLEFKYRPIGDLSPVAENDTVRLPDPTNTLLSNTDLSIVTTEHKNGSFDDSLSLNLGNSSKPRLDPVSIATAKLMNGSSNDFLSLGTSQNSETITASSDEFLFSDLGHLQKFSFDPLSMASTKPNKALSIVSIEAISNGLKLPATIKGQANEIFKVVESYARGKERNVLFAACIYIACRDNDMTRTMREISRFANKASISDISETVGFIAEKLEINKNWYMSIETANFIKRFCSIFRLDKEAVEAALEAAESYDYMTNGRRAPVSVAAGIVYVIARLSYEKHLLKGLIEATGVAENTIKGTYGDLYPNLPTIVPTWFANANDLKNLGAP

>P08796|CSA_DICDI Contact site A protein - Dictyostelium discoideum (Slime mold).

MKFLLVLIILYNILNSAHSAPTITAVSNGKFGVPTYITITGTGFTGTPVVTIGGQTCDPVIVANTASLQCQFSAQLAPGNSNFDVIVKVGGVPSTGGNGLFKYTPPTLSTIFPNNGRIGMILVDGPSNISGYKLNVNDSINSAMLSVTADSVSPTIYFLVPNTIAGGLLNLELIQPFGFSTIVTSKSVFSPTITSITPLAFDLTPTNVTVTGKYFGTTASVTMGSHIYTGLTVQDDGTNCHVIFTTRSVYESSNTITAKASTGVDMIYLDNQGNQQPITFTYNPPTITSTKQVNDSVEISTTNTGTDFTQISLTMGTSSPTNLVITGTNEKIVITLPHALPEGEIQFNLKAGISNVVTSTLLVTPVINSVTQAPHNGGSITISGIFLNNAHVSIVVDQNTTDIVCAPDSNGESIICPVEAGSGTINLVVTNYKNFASDPTIKTEATTSTTYTIPDTPTPTDTATPSPTPTETATPSPTPKPTSTPEETEAPSSATTLISPLSLIVIFISFVLLI

>O96759|ADAS_DICDI Alkyldihydroxyacetonephosphate synthase - Dictyostelium discoideum (Slime mold).

MSGEKKEYPKEHIDLYQQIKWNGWGDTRKFLHQLKPSGTIAMTTPEVSSVPLPSLRGFIKKELTLPGEEDKPFVLDETPALQIENIHVDPPKQYPEFVRELKAFFLPDQLKDDKLARITHTFGKSLRDLIRVRIGQVKNAPDLIVLPHSHEEVERLVQLAHKYNVVIIPMGGGSNIVGAIEPVSNERFTVSIDMRRMNKVLWVDRREMTACIQVGIMGPELEKQLHKQGVSLGHDPDSFEFSTLGGWLATCSSGHQSDKYGDIEDMAVSFRTVTPTGTLELRNGARSGAGINYKHIILGSEGTLGIITEAVMKVHAVPQAVEYYGFLFPTFAHAVSALQQIRSSEVIPTMIRVYDPEETQLSFAWKPSKGAVSEFTSAMVKKYLHYIRSFDFKNVCLSIIGFEGPKKVVDFHRTSVFDILSKNAAFGLGSAPGKTWAEKRYDLPYIRDFLLDHNMWVDVAETTVSYANLQTLWKDAKQTFVKHFKDQGIPAWICAHISHTYTNGVCLYFIFASKQNENKDMAQYIEAKKLMTDIIFKYGGSLSHHHGVGYEHVPWMTRYATRGWINVYRSLKETIDPKDICNPRKLIPTIKEENNKEPFLFDVVNVKYPKL

>P06660|HSP85_TRYCR Heat shock-like 85 kDa protein - Trypanosoma cruzi.

MTETFAFQAEINQLMSLIINTFYSNKEIFLRELISNSSDACDKIRYQSLTNQAVLGDESHLRIRVVPDKANKTLTVEDTGIGMTKAELVNNLGTIARSGTKAFMEALEAGGDMSMIGQFGVGFYSAYLVADRVTVVSKNNDDEAYTWESSAGGTFTVTPTPDCDLKRGTRIVLHLKEDQQEYLEERRLKDLIKKHSEFIGYDIELMVEKATEKEVTDEDEDEAAATKNEEGEEPKVEEVKDDAEEGEKKKKTKKVKEVTQEFVVQNKHKPLWTRDPKDVTKEEYAAFYKAISNDWEEPLSTKHFSVEGQLEFRAILFVPKRAPFDMFEPSKKRNNIKLYVRRVFIMDNCEDLCPEWLAFVRGVVDSEDLPLNISRENLQQNKILKVIRKNIVKKALELFEEIAENKEDYKKFYEQFGKNVKLGIHEDSANRKKLMELLRFHSSESGEDMTTLKDYVTRMKEGQKCIYYVTGDSKKKLETSPFIEQARRRGFEVLFMTEPIDEYVMQQVKDFEDKKFACLTKEGVHFEETEEEKKQREEEKTAYERLCKAMKDVLGDKVEKVVVSERLATSPCILVTSEFGWSAHMEQIMRNQALRDSSMSAYMMSKKTMEINPAHPIVKELKRRVEADENDKAVKDLVYLLFDTALLTSGFTLDDPTSYAERIHRMIKLGLSLDDEDNGNEEAEPAAAVPAEPVAGTSSMEQVD

>Q08168|HRP_PLABE 58 kDa phosphoprotein - Plasmodium berghei.

MDIEKIEDLKKFVASCEENPSILLKPELSFFKDFIESFGGKIKKDKMGYEKMKSEDSTEEKSDEEEEDEEEEEEEEEDDDPEKLELIKEEAVECPPLAPIIEGELSEEQIEEICKLKEEAVDLVENKKYEEALEKYNKIISFGNPSAMIYTKRASILLNLKRPKACIRDCTEALNLNVDSANAYKIRAKAYRYLGKWEFAHADMEQGQKIDYDENLWDMQKLIQEKYKKIYEKRRYKINKEEEKQRLKREKELKKKLAAKKKAEKMYKENNKRENYDSDSSDSSYSEPDFSGDFPGGMPGGMPGMPGGMGGMGGMPGMPGGFPGMPGGMPGGMPGGMGGMPGMPGGMPGGMGGMPGMPGGMPDLNSPEMKELFNNPQFFQMMQNMMSNPDLINKYASDPKYKNIFENLKNSDLGGMMGEKPKP

>P34137|PTP1_DICDI Tyrosine-protein phosphatase 1 - Dictyostelium discoideum (Slime mold).

MGSVESSNQMNGSIENKTNKIDISVLRPLPTRSNSSISLSSSSHSSFSRMGSLGSLPTNSGSSSPYYNNSSFDLVDEERIKSSIYNLKNHIKCIHKIKEEFRLLEESVGPSETSEGDKKHNTSKNRYTNILPVNHTRVQLKKIQDKEGSDYINANYIDGAYPKQFICTQGPLPNTIADFWRMVWENRCRIIVMLSRESENCRIKCDRYWPEQIGGEQFSIYGNGNEVFGTYSVELVEVIQDPEREIITRNIRLTFEGETRDITQYQYEGWPDHNIPDHTQPFRQLLHSITNRQNQIIPSSDRNVPIIVHCSAGVGRTGTFCTAVIMMKKLDHYFKQLDATPIDQVVDPFTHLPITEYQSDNLDLKGLGYHFKSSIYNSNGINNNNNNNLNNNNNINNNSNGSNNTPQTEPNNEEDDDDAAESDLKYAIMDKYNSRIDFNLFSIVLKLREQRPGMVQQLEQYLFCYKTILDEIYHRLNCKLGFSLPHVNNINNYNNYSNTTTTTTSSLASTTIIHPSTNSKLN

>P13816|GARP_PLAFF Glutamic acid-rich protein - Plasmodium falciparum (isolate FC27 / Papua New Guinea).

MNVLFLSYNICILFFVVCTLNFSTKCFSNGLLKNQNILNKSFDSITGRLLNETELEKNKDDNSKSETLLKEEKDEKDDVPTTSNDNLKNAHNNNEISSSTDPTNIINVNDKDNENSVDKKKDKKEKKHKKDKKEKKEKKDKKEKKDKKEKKHKKEKKHKKDKKKKENSEVMSLYKTGQHKPKNATEHGEENLDEEMVSEINNNAQGGLLLSSPYQYREQGGCGIISSVHETSNDTKDNDKENISEDKKEDHQQEEMLKTLDKKERKQKEKEMKEQEKIEKKKKKQEEKEKKKQEKERKKQEKKERKQKEKEMKKQKKIEKERKKKEEKEKKKKKHDKENEETMQQPDQTSEETNNEIMVPLPSPLTDVTTPEEHKEGEHKEEEHKEGEHKEGEHKEEEHKEEEHKKEEHKSKEHKSKGKKDKGKKDKGKHKKAKKEKVKKHVVKNVIEDEDKDGVEIINLEDKEACEEQHITVESRPLSQPQCKLIDEPEQLTLMDKSKVEEKNLSIQEQLIGTIGRVNVVPRRDNHKKKMAKIEEAELQKQKHVDKEEDKKEESKEVQEESKEVQEDEEEVEEDEEEEEEEEEEEEEEEEEEEEEEEEEEEEEEDEDEEDEDDAEEDEDDAEEDEDDAEEDDDEEDDDEEDDDEDEDEDEEDEEEEEEEEEESEKKIKRNLRKNAKI

>P08116|PVA_PLAFA Processed variable antigen (Fragment) - Plasmodium falciparum.

ETGESKETGESKETGESKETGESKETGESKETGESKETGESKETGESKETGESKETGESKETGESKETGESKETGESKETGESKETGESKETGESKETGESKETRIYEETKYNKITSEFRETENVKITEESKDREGNKVSGPYENSENSNVTSESEETKKLAEKEENEGEKLGENVNDGASENSEDPKKLTEQEENGTKESSEETKDDKPEENEKKADNKKKKK

>P35401|CRAC_DICDI Protein CRAC - Dictyostelium discoideum (Slime mold).

MGKTERKKELLELFEYEKIKGDVSYSSIMKKAGGNGKGFLDRYFALHRNYILYYKLGKSSLKPDDKQEPQGYINLMDCNPDDTKEIAPLMFQISHKHRTYIVKAKDESSMKQFLTLLIARIRSLEKIDIDKLGCTVVVLTKVKKFREVLTNPLILPDRVSPEMAEEWVKQMKNYNASFNLADPFIKQVEQISEFCRGEVKEYIDWFGGPEGPRLAMIRCEETVLSNWVEYINKTSSEITTYQDNRFFREDFKDIAVHLKNMTTFIDCYNDYMIHCRKYNNNKPNTKFLEEKQTFKEYIEKFIPKVASCNDVSLNQFYDRSLIQSSDGIVTINTSGIKKTLINQSNIISITSTTTTTTTTTTTTCSMPNMSNLIHSLDHTNLNIIDLNHSKSQQQLHPPPSPHHQHLHHQIVSNSKDFNISVSSNNFNDGNSEFPNLDINCDFDLTSASNLSSPILSSEVPSNVVDPIGSGQGGGGSGGGGVTAVTEEAINEKWHFDCNTSMIFKPPSEDGRNEGSNMSTSSITSKMSLSLNGGFDMKWVYQCGYFKSKNMGSISWNGKHWCWSHPRTSYKIKYIWDPTKQSFLNIPFKSRVGATGGGSVPSSQSTNNLQSSTSSMSSLSSSSTSTTKRSHPTTLYPDYQFKDNLLTPIIIEGRHQPSLTLIDSPLTIPNACLLTIAMTQYIQDALIHLSLGPKVLSSK

>P14325|SYQ_DICDI Probable glutaminyl-tRNA synthetase - Dictyostelium discoideum (Slime mold).

MSTKPTINKDELVTLFSQIGLDSSKAKETTNNATLSSNLQEIIKEAGAESGCEKSVGLLLYTLATKYPANAMKHRATLVDYIANKKSVNSINLQACLDYLRRTANEELNVAEFEQSCGVGVVITREQVAQAVSDYINKNKSDLLEKRYQFNIGGILMEIKNSLKWANAKDIKEEVDAAILSLLGPKTDADKAPPAKPVKPTTPTAVATTTAATTTTGDLSPIIPAELKPAKEEIKFPDPSDNIQNTPKLLADHLKTTGGKIVTRFPPEPNGYLHIGHAKAMHLNFGYAKKNGGKCYLRFDDTNPEKENQEYIDSIIDSVKWLGHEPCEITYSSSQFDTLYEMANELIRRGYAYVCHQTASEISEGREKMTDSPYRNRTVEENLKLFEDMRLGKFEEGKAILRMKGDMKHPNPCMRDLIAYRIKYHHHPMSGDKWCIYPSYDYTHCLVDSIENITHSLCTLEFEIRRLTYNWLIDVLGLYRPVVWEYARLNLTHTVLSKRKIITLVQNKIVNGWDDPRLSTLNAFRRKGYTPEAINLLCDTIGVTRTNGTTISYELLELCCRQDLDGKATRAMAVFDPIKVVITNYPEDKSEEINAPNIPSKPEKGTHKIDFSRIVYIERSDFRMEDNKDFFGLAPGKEILLKYAYNIKCEKVIQDADGKVTELHVTYDKDNSSKKLKTIHWVSSVAGTEPMKAEVRLYEHLFKDSEIGDDWLNNINPNSLRIIPNAFIDKTVLASKEYDRYQFERVGYFVVDKDTTSDKMVFNRTVSLKENKEKSKSRN

>P50650|RIR2_PLAF4 Ribonucleoside-diphosphate reductase small chain - Plasmodium falciparum (isolate Dd2).

MADVINISRIPIFSKQEREFSDLQKGKEINEKILNKESDRFTLYPILYPDVWDFYKKAEASFWTAEEIDLSSDLKDFEKLNENEKHFIKHVLAFFAASDGIVLENLASKFLREVQITEAKKFYSFQIAVENIHSETYSLLIDNYIKDEKERLNLFHAIENIPAVKNKALWAAKWINDTNSFAERIVANACVEGILFSGSFCAIFWFKKQNKLHGLTFSNELISRDEGLHTDFNCLIYSLLDNKLPEQIIQNIVKEAVEVERSFICESLPCDLIGMNSRLMSQYIEFVADRLLECLGCSKIFHSKNPFNWMDLISLQGKTNFFEKRVADYQKSGVMAQRKDQVFCLNTEF

>P90520|TOP2M_DICDI DNA topoisomerase II, mitochondrial - Dictyostelium discoideum (Slime mold).

MSKLLNNNNHKNLTNYLKFGKGIINNLNNKSKQVGIISFISQSSIQSQSSIQSQSFLSINNNSNNKYFSTKLNKNEKISEKTTTRKIEDIYQKKTPTEHVLLRPDSYIGTIEKIEDDMWVLSNSMFNKEKKTIELNNDNNEKNVESTTTTTTKTNKKPLTYIHPIKATYIPGLLKIYDEILVNAADNKKRDSKMSFIKVEINPNENSISIMNDGKGIPVVMHQTENCYVVEMVMGNLMSGSNFNDSELKVVGGRNGFGAKLTNIFSKEFTVETVDKSSGKKYFQRWSNNMGDRSEPIITPIGEGESDYTKITFKPDLEKFKIKSLWDDNILQLMERRLYDIAGCNTELMVTLNGKRLNYNFQSYVKLYEHHLNNSTKREDNEEQYREESFEFGEISPRWKIGIGLSETGQFTQVSFVNSINTVKGGTHVNFLADQIVRYVGEKLKKKHSDLEIRPMNIKHHLALFVNCLVDNPSFDSQSKETLTTKPMLFGSTPEIPESLLAQFVKNSKIIERVAGWALMKQKADLIHSTSGRQSKTTLIKSISKLDDANWAGGLKSKECTLIITEGDSAKSLALAGLSVVGRNSYGVFPLRGKLLNVRDVASKQLLSNEEINNLTTILGLSHKNSYDTDESMEDLRYGRVMIMADQDHDGSHIKGLVMNFIHYFWPNLLKRGFLVEFVTPIIKATKSSTQKKSFFTIKDYEKWRETISSDQLKQYTIKYYKGLGTSTSAEAKEYFSNLDKHVIKFIWGDEADDLIKMAFAKDLSSLRQRWIKETDMSQGIDHSIKEITYPDFINKELIHYSWAANLRSIPSLIDGLKPGQRKILFASFKRRLTNEIKVSQLSGYVAEQTSYHHGEQSLNSTIVKMAHNFVGSNNLPLLTPSGQFGTRLQGGSDSASARYIFTKLEPVARYLFNELDDPLLNYLEEEGESIQPDYYIPIIPMLLVNGSEGIGVGMSTSIPLFSPIDIIDQLMLRLNNQVALKKLIPWYRGFKGTISPDRHTYRTNGVIKLVGRNLEITELPIGRWTSDYKEVLNDLIDKDVIKSFQESNTENSVHFTILLNNNQLEQMEDLTENELIKLFKLSASLNFHLTCFDENSKIQKLESVEEIIDQFYKVRLQFYGKRREYLLKSLDNQIKRLTTTIQFLEVIASGKLKIQGRSKQDLIKELESGEIVGFENFGTHPPEVYQHLFSLSILDITKERIDNLTNQLTKRKSEHQSISSSDPKSLWTADLQQLKEYLEKSDKEFQKKPLKTSSSSSFDVSSSSESAKLSSTRKSKTDKIKSK

>O96935|AMP1_PLAFQ M1 family aminopeptidase - Plasmodium falciparum (isolate FcB1 / Columbia).

MKLTKGCAYKYIIFTVLILANILYDNKKRCMIKKNLRISSCGIISRLLKSNSNYNSFNKNYNFTSAISELQFSNFWNLDILQKDIFSNIHNNKNKPQSYIIHKRLMSEKGDNNNNNHQNNNGNDNKKRLGSVVNNEENTCSDKRMKPFEEGHGITQVDKMNNNSDHLQQNGVMNLNSNNVENNNNNNSVVVKKNEPKIHYRKDYKPSGFIINNVTLNINIHDNETIVRSVLDMDISKHNVGEDLVFDGVGLKINEISINNKKLVEGEEYTYDNEFLTIFSKFVPKSKFAFSSEVIIHPETNYALTGLYKSKNIIVSQCEATGFRRITFFIDRPDMMAKYDVTVTADKEKYPVLLSNGDKVNEFEIPGGRHGARFNDPHLKPCYLFAVVAGDLKHLSATYITKYTKKKVELYVFSEEKYVSKLQWALECLKKSMAFDEDYFGLEYDLSRLNLVAVSDFNVGAMENKGLNIFNANSLLASKKNSIDFSYARILTVVGHEYFHNYTGNRVTLRDWFQLTLKEGLTVHRENLFSEEMTKTVTTRLSHVDLLRSVQFLEDSSPLSHPIRPESYVSMENFYTTTVYDKGSEVMRMYLTILGEEYYKKGFDIYIKKNDGNTATCEDFNYAMEQAYKMKKADNSANLNQYLLWFSQSGTPHVSFKYNYDAEKKQYSIHVNQYTKPDENQKEKKPLFIPISVGLINPENGKEMISQTTLELTKESDTFVFNNIAVKPIPSLFRGFSAPVYIEDNLTDEERILLLKYDSDAFVRYNSCTNIYMKQILMNYNEFLKAKNEKLESFNLTPVNAQFIDAIKYLLEDPHADAGFKSYIVSLPQDRYIINFVSNLDTDVLADTKEYIYKQIGDKLNDVYYKMFKSLEAKADDLTYFNDESHVDFDQMNMRTLRNTLLSLLSKAQYPNILNEIIEHSKSPYPSNWLTSLSVSAYFDKYFELYDKTYKLSKDDELLLQEWLKTVSRSDRKDIYEILKKLENEVLKDSKNPNDIRAVYLPFTNNLRRFHDISGKGYKLIAEVITKTDKFNPMVATQLCEPFKLWNKLDTKRQELMLNEMNTMLQEPNISNNLKEYLLRLTNKL

>P16893|TRAP_PLAFA Thrombospondin-related anonymous protein - Plasmodium falciparum.

MNHLGNVKYLVIVFLIFFDLFLVNGRDVQNNIVDEIKYSEEVCNDQVDLYLLMDCSGSIRRHNWVNHAVPLAMKLIQQLNLNDNAIHLYVNVFSNNAKEIIRLHSDASKNKEKALIIIRSLLSTNLPYGRTNLTDALLQVRKHLNDRINRENANQLVVILTDGIPDSIQDSLKESRKLSDRGVKIAVFGIGQGINVAFNRFLVGCHPSDGKCNLYADSAWENVKNVIGPFMKAVCVEVEKTASCGVWDEWSPCSVTCGKGTRSRKREILHEGCTSEIQEQCEEERCPPKWEPLDVPDEPEDDQPRPRGDNSSVQKPEENIIDNNPQEPSPNPEEGKDENPNGFDLDENPENPPNPDIPEQKPNIPEDSEKEVPSDVPKNPEDDREENFDIPKKPENKHDNQNNLPNDKSDRNIPYSPLPPKVLDNERKQSDPQSQDNNGNRHVPNSEDRETRPHGRNNENRSYNRKYNDTPKHPEREEHEKPDNNKKKGESDNKYKIAGGIAGGLALLACAGLAYKFVVPGAATPYAGEPAPFDETLGEEDKDLDEPEQFRLPEENEWN

>P34940|CH60_PLAFG Chaperonin CPN60, mitochondrial - Plasmodium falciparum (isolate FCR-3 / Gambia).

MRMKRIHILFVVIFLLCLRYGYSIKKKRSPNNKNRLFINKRLKYINSKIISRRKENYVKMKMTENKVKGKDIIYGNECRNELLKGILTVSDVVKLTLGPRGRNVLLEKEYGSPLIINDGVTIAKNISLKDRKKNNGVKLMQESTNISNDKAGDGTSSTALMTATITKKGIEQVNRNHNPIPIQRGIQLASKMIIEKIKSLSTPIKTYKDILNIATIASNNDVHMGQIIANAYDKLGKNAAIILDDNADINDKLEFTEGYNFDRGIINPYLLYNENKDYIEYSNVSTLITDQNIDNIQSILPILEIFAKNKQPLCIIADDFSNEVLQTLIINKLKGAIKVLCIVTNSKYISADVGLDLNNLHNNMSSFDNNYLSLLGSANTLIVKKDRTSLITKEEYKKEIDERINVLKKEYEETTSKYDKEKLNERIAALSGGIAKILIGGNSETEQKERKFKYEDATNAVKSAIDIGYVPGGGVTYLEIIKSNFIQEIHKKIEEDLQISSNNDEKKYLELIGNLESEMELQKMGANIVVSSLDVITKQIADNAGVNGDNVVKIILNSKDKYGFGYDVNTNKFVNMVEKGIIDSTNVIISVIKNSCSIASMVLTTECMMVDHEKKDKGILDSSINSPNYLSKHRRTYKHKLHDDEDTDEDDEEDEDDEDDEDDLDDDDYDDEDEEDEEDEEDEDDEDDEDSMNDEYNYDE

>Q7M3S9|RNGB_DICDI RING finger protein B - Dictyostelium discoideum (Slime mold).

MKIDCKRVSLIGSPEPRWGHTGTTLPNGSGFIVFGGNSNRAFNDIQYYNIFNNSWSKIEAVGNAPSERYGHSAVLYQSQSRPYSDSYQIIFFGGRATSKPFSDINILYVNSNRSFIWKQVTTKSIEGRAGHTAVVYRQNLVVFGGHNNHKSKYYNSVLLFSLESNEWRQQVCGGVIPSARATHSTFQVNNNKMFIFGGYDGKKYYNDIYYLDLETWIWKKVEAKGTPPKPRSGHSATMIQNNKLMIFGGCGSDSNFLNDIHILHIEGANEYRWEQPSYLGLEIPQARFRHTTNFIGGRVYIYAGTGSGNLMGDLHTLEFLDDNNTPLIPITISIPITNSNSIVGSPNTSISCGVSNSGASSSSGGGISGHPSILSSSSSSSYLSTSPLSTSSLASSYQSSQSLQFNQNQNQNNNNNNNNNNNNNIQTTTTTTTNNNNNNNNNNNNNNNNNNVESNQQQQQIQHQTSPMSVLSRSNSNISLNSLNSSSSSILSTPSTLSTTTTTTTTSHASHTSHTSNRSNGSRGGIPSIPPFNGRSSNHNNNNNSNSNNYNNHQQTKTNSAEELILEELKSLNIYDQAACNKDFQTNLKRVEELFNQKIKHEQKYRQSLEEKLGKANHQVSLLTNQIQSIIQKDELTSLKKEYSELKKKHSLLYSEDIDDLPTETCLKLEEIHVKSLEKLRVKKLPSSNQLSTLQQQIPQQPTTIICNNSQIIQQQPLPPLQQQQQQQQQQQQQQQQQQQPLEIQEQLTMLQLQLSQLSQQQQNQIDKQQKQEKLQQEQQQQQLKNINRLSISSNSSTLSSKDSFYFESKIQELSNQLKEKQQAITDRDNKIKDFENQLNKYKLIGLDSLDHYQLLELESSFHNGLKQIGSIKDQRYLNRLVSLEKEKDQLKDQNSCVICASNPPNIVLLPCRHSSLCSDCCSKLTKCPICRSHIENKISIYQ

>P34715|EF1G_TRYCR Elongation factor 1-gamma - Trypanosoma cruzi.

MSLTLWSGVNPENARTHKLLAAAALANVAVTLKACEYGRENETAEYCRNCSPCGRYPVLQTEEGCVFESNAILRHIARLDRSGGFLYGRTPLEGSQVDMWLDFSATELDAASEPFVHHAFRGEPLPANAMDRVHEVLRALEAWLETRTFLVGERMTVADVAVAFALQWHYRLNGAEGEALTKKYRNAYRMYNTVMQQPKTVEVLRSQGATFGAREGGAKGQGRGCARPGREEAERAAAAADGAEEEDEAPREKKKPNPLDELPPSPFVLDAFKREYSNTDTRTVAAPYFFQHYDAAGYTTFWCRYKYNEDNKMQFMTANLIRGWFQRMEHVRKYAFGVALIIGEERRHDIVALWVFRGRGMPAIVEDVEDTELFDWEEVADVAAQRERITDYLSWEGPTIPRPVLEGRVFK

>P30840|ALDH1_ENTHI Aldehyde dehydrogenase 1 - Entamoeba histolytica.

MEAYVLSLSDVLFNILFIGVCILSVLLLISHALKYIIGDSKEKKLFNQRLEQIKNQQPLEPTKYQDIQTICKTLKESYSTNALRHLDARKEVLYCLYRMVLDNKQAISNAIREDLHRDVGMCVAEVNSVIHEINFLRKNLNKYLKRKQVPTVCAQLFGKSFVEREPYGCVCVISPWNFPANLSLIPCAGALACGNTVFLKMSKYSMATSKLIAELCDKYIPSEYLRCEYLTGREAIQECCSASFDYYFFTGSTYVGKLINQAAAEKMVPATLELGGKNPAIVDKSVNLKVAAKRIAWAKSINAGQICVCVDHVFVPRSIKNEFCEAVKNSFIKFFGEDQKKSEDFGRIITKSAAKKMKEIIDQSDVYYGGEVDIENKYVQPTILQNVKIDDLCMKEEIFGPILPVIEYDTLDEVFEMVKQHPNPLACYVFTEDNDMFEHVIANINSGAIYNNDSIVHLLNPNLPFGGNCQSGIGCYHGKYTFDTFSRPRAVCNGHTSFDLSLKDWPFTSFQSWAVDRMAASEIPVVSYL

>P32256|TBB_DICDI Tubulin beta chain - Dictyostelium discoideum (Slime mold).

MREIVQIQAGQCGNQIGSKFWEVISEEHGIQSDGFHAGGEDEHLKRLQLERINVYYNEARDGKYVPRSVLVDLEPGTVDTIKASPYGKLFRPDNFIHGQSGAGNNWAKGHYTEGVELVESVLDVVRRETEGCDCLQGFQVTHSIGGGTGSGLGTLLISKIREEFPDRMMCTFSVVPSPKVSLTVVEPYNATLSVHQLVENADEVMCIDNEALHDICFRTLKLTQPNYGDLNHLISSVMSGITCCLRFPGQLNSDLRKLAVNLIPFPRLHFFLVGFAPLTAKGASSYNRITVPELTQQMFDAKNMMAASDPHNGKYLTASALFRGKIFTKEVDEQMHNIQTKNSSYFVEWIPHNIKSSICDIPPKGTPMAVTFIGNNTAIQDLFKRISIHFQAMFRRKAFLHWYTLEGMEELEFTEAESNMNDLVYEYQQYSNQETEEDGGEYQEEHEEHEEQAEN

>P05095|ACTN_DICDI Alpha-actinin, non-muscular - Dictyostelium discoideum (Slime mold).

MSEEPTPVSGNDKQLLNKAWEITQKKTFTAWCNSHLRKLGSSIEQIDTDFTDGIKLAQLLEVISNDPVFKVNKTPKLRIHNIQNVGLCLKHIESHGVKLVGIGAEELVDKNLKMTLGMIWTIILRFAIQDISIEELSAKEALLLWCQRKTEGYDRVKVGNFHTSFQDGLAFCALIHKHRPDLINFDSLNKDDKAGNLQLAFDIAEKELDIPKMLDVSDMLDVVRPDERSVMTYVAQYYHHFSASRKAETAGKQVGKVLDTFMLLEQTKSDYLKRANELVQWINDKQASLESRDFGDSIESVQSFMNAHKEYKKTEKPPKGQEVSELEAIYNSLQTKLRLIKREPFVAPAGLTPNEIDSTWSALEKAEQEHAEALRIELKRQKKIAVLLQKYNRILKKLENWATTKSVYLGSNETGDSITAVQAKLKNLEAFDGECQSLEGQSNSDLLSILAQLTELNYNGVPELTERKDTFFAQQWTGVKSSAETYKNTLLAELERLQKIEDSLVEFAKRAAQLNVWIEAADDHVFDPINVDSVQGVQEIQEKFDAFLHDQSQQFAELEALAALTQQLRELGRSENDYSVISYDELSAKWNNLLAGIEERKVQLANELTTQTNNDVLCQSFSVKANEISDYVRVTLDAISQNTSSDPQEQLNNIRAIITAHAEKKPELDELYTIASQLEEAQVVDNKHTQHSLESIKLKWDKLNTLAKKNEQVVEGEILAKQLTGVTAEELSEFKACFSHFDKDNDNKLNRLEFSSCLKSIGDELTEEQLNQVISKIDTDGNGTISFEEFIDYMVSSRKGTDSVESTKAAFKVMAEDKDFITEAQIRAAISDSKQIDYLLASMPAVEGGFDYNSFAEKLYQ

>P16405|ORA_PLAFN Octapeptide-repeat antigen (Fragment) - Plasmodium falciparum (isolate NF7 / Ghana).

KKVLSFSHSLNTYEGTGVPEKIYNEEKNNGKFRLLGLYGNNSTNWLITDCACMISGVTTLVMHSKFSIDIIIDILNNTKLEWLCLDLDLVEGLLCRKNELPYLKKLIILDNLTKRSEMKIENEEKSNGSRKSSNKQKYNESDKREDISLCALECDKEKIEKINSLKEKAKTLGLSIIVFDNMTENKIANVTVQNEDPNFIASIVYTSGTSGKPKGVMLSNRNLYNGVIPPCDCNIIKKYPLTTHLSYLPVSHIYERVIFFIALFLGVKINIWSRDIKFLNTDICNSKAEIILGVPKVFNRMYATIMTKINNLSRCKKWIAKQAINLRKGKNNGNFSKVVEGITNISRKIKDKINPNMDVILNGGGKLSPEVAEGLSVLLNVKYYQGYGLTESTGPIFLQDVDDCNTESMGVAVSPSTRYKVRTWEIYKATDTIPKGELLIKSDSMFSGYFLEKESTEHAFTNDGYFKTGDIVQINDNGSLTFLDRSKGLVKLSQGEYIETEMINNLYSQIPFVNFCVAYGDDSMDGPLGIISVDKHKLFTFLKNDNMLKTTGVDEKNFSEKLIDETLNDPIYVDYVKGKMMEIYKKTNLNRYNVINDIYLTSKPWDTTNYLTPTLKIRRFNVFKDFSFFIDEVKKKYEEKLSGSSTGSMNNGKSGSKSDIKGGSKDDIKSGSKDDIKSGSKADIKSGSKDDIKSGSKDHIK

>P13021|CAPZB_DICDI F-actin-capping protein subunit beta - Dictyostelium discoideum (Slime mold).

MTEKQLSCCLDLMRRLPPSQIEDNLAGLLDLVPDLTEDLLSSIDQPLKVAYDAVSKKDYLLCDYNRDADSYRSPWSNKYDPPLSGACYPSSKLRDIEVQANEIFEIYLNLYFEGGVSSVYCWDLDDNFAAVVLMKKTQDQSKKGQPMRGTWDSIHVVEVKLGKKDKAVYKLTSTVMLSIETDNDNTGKVNLAGSLTRQDEKEYTFNEVDTHCVNIGKMVEDMESKLRQTLETIYFGKTKEVVNTLRNATGNSELEKRKNLSNQIGSAIGNRG

>Q25998|PRI1_PLAFK DNA primase small subunit - Plasmodium falciparum (isolate K1 / Thailand).

MKMEIVGDIKDSIVNENDLIFYYRSLCPINDLYNWLNYKNDIKGKYTKLNDPHFFSKREFSFTCKKSDQGKEEIYIRWLSFSNPEEFKNKLLSDLVPIKFDIGAIYNFPVSQKDQKGDIFLPVQKELIFDIDMNDYDDIRTCCTDKKVCKLCWKFLTVAIVLLDTALREDFSFEHILWVYSGRRGIHCWVADESCRYYTTDARAALADYLNILSGSDTKKKKVSIWGKDKYPMFERAFDICYKYFDVLMEEQDFFKKGSPHVQKLIDYLPYASGKVTDPLKAMKLNELKEYINNNNFNSREIFEKFSSIYNFLTPSNYFKRKNVSGNINMPSFVKEIVFHFTYPRLDINVSKEINHLLKSPFCIHNSTGRVCVPLDIKNINNFNPQSVPTLKLLREQFDDPKNSHIEAENRTSLKPYIDYFRRHFIENILLSCVEKKKRLNENSKYVDYNNI

>P21303|PK66_PLAKU Merozoite receptor PK66 - Plasmodium knowlesi (strain nuri).

MNKIYYILFLSAQCLVHMGKCERNQKTTRLTRSANNASLEKGPIIERSIRMSNPWKAFMEKYDLERAHNSGIRIDLGEDAEVGNSKYRIPAGKCPVFGKGIVIENSNVSFLTPVATGAQRLKEGGFAFPNADDHISPITIANLKERYKENADLMKLNDIALCKTHAASFVIAEDQNTSYRHPAVYDEKNKTCYMLYLSAQENMGPRYCSPDSQNKDAMFCFKPDKNENFDNLVYLSKNVSNDWENKCPRKNLGNAKFGLWVDGNCEEIPYVNEVEARSLRECNRIVFEASASDQPRQYEEELTDYEKIQEGFRQNNRDMIKSAFLPVGAFNSDNFKSKGRGYNWANFDSVNNKCYIFNTKPTCLINDKNFFATTALSHPQEVDNEFPCSIYKDEIEREIKKQSRNMNLYSVDKERIVLPRIFISTDKESIKCPCEPEHISNSTCNFYVCNCVEKRAEIKENNEVIIKEEFKEDYENPDGKHKKKMLLIIIGVTGAVCVVAVASLFYFRKKAQDDKYDKMDQAEAYGKTANTRKDEMLDPEASFWGEDKRASHTTPVLMEKPYY

>Q02752|PHPA_PLACH Acidic phosphoprotein - Plasmodium chabaudi.

MKAISLGLISSIIFSIVLAKNSSGSGSSTGCFGCFRKKPKKKILATEVAKPVKAPETADFDPKLPNLKFIEEFEPITIEGCKSRLHELDEPFVSETDGMIIDKVTGFSRRENDSVLSGWYIRPYEEGYENMIKVNFIPLREYYKRMENRPPKQYDGPPPIPDMPQGYVPPKKEEIPVEQYVIQLSEEDPYLLQEEDALSLMEYDAETLNEGDAETLNEGDAETLNEYDAGTLNEEDAGTTNEAGEGTTNEEGEGAANEYDAETLNEYDADTLNEYDAGTLNEYDAGTLNEEEGSTTNEAGEGTSNEAGEGTANDDEELDEEVASIFDDDEHADDLSLLDYDENSNENQENVKKGNENEGEQKGNENEGEQKGKKKKAKEKSKKKVKNKPTMTTKKKKKKEKKKKKKEKEKKKEKKVKVEVIMDHFSEMEKMMNNKIKHWNK

>P14132|RS9_DICDI 40S ribosomal protein S9 - Dictyostelium discoideum (Slime mold).

MSSNYSKTSHTPRRPFEKERIDAELKVVGEFGLKNKNEVWRVQYALAKIRKAARELLVLDEKDPKRIFEGSALLRRLHKLGVMEESKNKLDYILNLKVQDFMERRLQTLVFKNGLAKSIHHARVLIKGRHIRVGKQLVNVPSFLVRVESQKHLGLASTSPLAGGRPGRRARKMAKNNSSKGEEEN

>Q01501|VDAC_DICDI Outer mitochondrial membrane protein porin - Dictyostelium discoideum (Slime mold).

MNPGLYADLTKPTADFIKKDFAETFKLDTTFKGKYGSIVAVTDIKDSGVVASIQPKADFTKYLGKVSNGNFTVDTNGVKKGEFTIENIIPGLKAVANGDSKQNFSTEFQYKKDKIAFTLFGHNNKSFNTSLAFLINPTFSVGVQAEGNAKNTLKNVNATITIRPRPDVFVSIVDRFMDKQILLSTLYTATSKLSFAGDVTVDLKASEKAPSFNVGTQYKIDSASLLKAKVNNNRKVNISYIYNTSNNTKFVLGWNVNTKNFKQGNTFGATVNLTL

>P22620|ABRA_PLAFC 101 kDa malaria antigen - Plasmodium falciparum (isolate Camp / Malaysia).

MMNMKIVLFSLLLFVIRWNIISCNKNDKNQGVDMNVLNNYENLFKFVKCEYCNEHTYVKGKKAPSDPQCADIKEECKELLKEKQYTDSVTYLMDGFKSANNSANNGKKNNAEEMKNLVNFLQSHKKLIKALKKNIESIQNKKHLIYKNKSYNPLLLSCVKKMNMLKENVDYIQKNQNLFKELMNQKATYSFVNTKKKIISLKSQGHKKETSQNQNENNDNQKYQEVNDEDDVNDEEDTNDDEDTNDEEDTNDDEDTNDDEDTNDEEDTNDEEDHENNNATAYELGIVPVNDVLNVNMKNMITGNNFMDVVKNTLAQSGGLGSNDLINFLNQGKEIGENLLNITKMNLGDKNNLESFPLDELNMLKDNLINYEFILDNLKTSVLNKLKDLLLRLLYKAYVSYKKRKAQEKGLPEPTVTNEEYVEELKKGILDMGIKLLFSKVKSLLKKLKNKIFPKKKEDNQAVDTKSMEEPKVKAQPALRGVEPTEDSNIMNSINNVMDEIDFFEKELIENNNTPNVVPPTQSKKKNKNETVSGMDENFDNHPENYFKEEYYYDENDDMEVKVKKIGVTLKKFEPLKNGNVSETIKLIHLGNKDKKHIEAINNDIQIIKQELQAIYNELMNYTNGNKNIQQIFQQNILENDVLNQETEEEMEKQVEAITKQIEAEVDALAPKNKEEEEKEKEKEKEKEEKEKEEKEKEEKEKEKEEKEKEKEEKEEEKKEKEEEQEEEEEEIVPENLTTEESK

>P54637|PTP3_DICDI Tyrosine-protein phosphatase 3 - Dictyostelium discoideum (Slime mold).

MISSSMSYRHSTNSVYTLNPHLNIPISTSTTIPPTSFYANNTPEMIQSQSENTNTNNINNSSSNINNNNNNTPDSMSMSTSLSSSPSVSFNHLDLNSINNKINNNTTTNNNNNNNNNNDDKFDTNALKLSNTMIIKNNNNNNNNNNNNNNNNNNNNNNNNNNNNNNNNNNNNNNNNNNNNNNNNNNNSNSNIEINVPSIQFDNEPAMEVDSVAPLNVPSNHTRTTLAMHNTKSLSTSNIGLLNILPNQQSSSSSSLSSTTTTTTTTSSSLLMPQSLFNNSTYNNHHNNNNSSNAGIVGGLNGSTSSLPTQAQVQLQQMQQQMQQHQQHQYKKANLSSLSTVVDNNLNNNPMNTSTSSPAQPNASPFSFSSSSLFSNSSLSNSGSGSASTTSTSTSSSNSMSSSPPPSLKTSFSQLDEDREKMRLEFEMIKKPEMASKKSHKHHQRHYSHNDLDNRKHDEEKFFSALQPNNYGKNRYHDVLPNESTRVRLTPIESGDGDYINANYINGEVPNSYRYYIACQAPLPSTIKDFWRMVWEERSSVIVCLTKLEENGKKKADVYYPETSQAQEYGSFWIHLHKKVMFKDIGVSSLHLYKKGEEFPREVVLLHYTQWPDCGAPPSSSHIRTLSVMVNTFKARGSAKNTNGPVIVHCSAGIGRSGTFISININMAKIERFGNDPSQMNISIKDSVLELRRQRRGMVQTLDQYIFIFKVINDVLTDMGIRSLSSPSKRRSCEMIKSTPMPRLDISIPPPLTFTPKDFQSSISPSTDMIASLSIITQMTQTLKFPPQQQQDNPFSKSSIKISPSPLNSTNISIPKNQQFQHPFQIQPQLDLNLQQQQQQSSQQLNDNPPLNMSSNSIKFPPVTSLSSCHLFEDSKNNDNNNKQQQQQQQQQQKNNQQCSGFSHFLNNNNNNDNNGSSGGGFNGSFLFNSNNSGSSSTNSECSNNNKNNNNNSNNNNNNNNNKNSDNNGTKDKDENDSCESPRVTPIKCF

>Q94469|G3P_DICDI Glyceraldehyde-3-phosphate dehydrogenase - Dictyostelium discoideum (Slime mold).

MVVPIGINGFGRIGRLVLRASLENPECRVLAINEPFMDVKYMVYMFKYDSTHGRFKGTVEDINGEFVVNGNKIHVFAEKDPANIKWSSVGAEYIVESTGLFLSTEKAGVHLKGGAKKVVISAPSTDAPMYVMGVNEETYESSHDVISNASCTTNCLAPLAKIIHENFGIVEGLMTTVHAITATQKTVDGPSGKDWRAGRSALSNIIPASTGAAKAVGKVLPALNGKLTGMSFRVPNCDVSVVDLTVRLEKKATYEEIKKVMKAASESDKYKRYIGYTEDEVVSTDFIGDTHSSIFDAHAGIALNDNFVKLVSWYDNEMGYSTRVIDLLVYISKKN

>P69192|SERA_PLAFG Serine-repeat antigen protein - Plasmodium falciparum (isolate FCR-3 / Gambia).

MKSYISLFFILCVIFNKNVIKCTGESQTGNTGGGQAGNTVGDQAGSTGGSPQGSTGASQPGSSEPSNPVSSGHSVSTVSVSQTSTSSEKQDTIQVKSALLKDYMGLKVTGPCNENFIMFLVPHIYIDVDTEDTNIELRTTLKETNNAISFESNSGSLEKKKYVKLPSNGTTGEQGSSTGTVRGDTEPISDSSSSSSSSSSSSSSSSSSSSSSSSSSSSSSSSSSSESLPANGPDSPTVKPPRNLQNICETGKNFKLVVYIKENTLIIKWKVYGETKDTTENNKVDVRKYLINEKETPFTSILIHAYKEHNGTNLIESKNYALGSDIPEKCDTLASNCFLSGNFNIEKCFQCALLVEKENKNDVCYKYLSEDIVSNFKEIKAETEDDDEDDYTEYKLTESIDNILVKMFKTNENNDKSELIKLEEVDDSLKLELMNYCSLLKDVDTTGTLDNYGMGNEMDIFNNLKRLLIYHSEENINTLKNKFRNAAVCLKNVDDWIVNKRGLVLPELNYDLEYFNEHLYNDKNSPEDKDNKGKGVVHVDTTLEKEDTLSYDNSDNMFCNKEYCNRLKDENNCISNLQVEDQGNCDTSWIFASKYHLETIRCMKGYEPTKISALYVANCYKGEHKDRCDEGSSPMEFLQIIEDYGFLPAESNYPYNYVKVGEQCPKVEDHWMNLWDNGKILHNKNEPNSLDGKGYTAYESERFHDNMDAFVKIIKTEVMNKGSVIAYIKAENVMGYEFSGKKVQNLCGDDTADHAVNIVGYGNYVNSEGEKKSYWIVRNSWGPYWGDEGYFKVDMYGPTHCHFNFIHSVVIFNVDLPMNNKTTKKESKIYDYYLKASPEFYHNLYFKNFNVGKKNLFSEKEDNENNKKLGNNYIIFGQDTAGSGQSGKESNTALESAGTSNEVSERVHVYHILKHIKDGKIRMGMRKYIDTQDVNKKHSCTRSYAFNPENYEKCVNLCNVNWKTCEEKTSPGLCLSKLDTNNECYFCYV

>Q37311|CYB_DICDI Cytochrome b - Dictyostelium discoideum (Slime mold).

MRLVKKNVVINGIYEAGVRYPEPANISYLWNFGFFSLICLIIQLVSGILLAMHYSAHVDLAFNSIERLVREVDYGWLLRYIHANGASFFFIVVYIHMLRGLYFGSYQKPNAMLWVSGVVIFLLLIITGFLGYVLPWGQMSYWAATVITNLVTVLPVIGEDIVIWLWGGFNVDNPTLNRFFSLHYLCPFIIVGLVGLHIIFLRENGSTNPLGVKSHVDQIPFTPYFTIKDLFSFMIFLVLFFTFVFFAPNYLGHPDNYLMADSNVTPAHIVPEWYLLPFYAMLRSIPNKVLGVLALVLAIVVLAFLPFLTIAEVRSSYFRKIHKHLFWSFLALCFFLGFLGSQPAAAPYLICGLYSTIAYFIYILVLFPCIYIVEKMIIKTIMKTTVKKA

>P54705|SNWA_DICDI Protein snwA - Dictyostelium discoideum (Slime mold).

MTSLSSLLPKPKNVYSNEEEDPLFQPKPKPQQQKQQQQQQQELNDKPKKVIPTYGNRKGYLPKNIEDFGDGGAFPEIHIVQYPLDMGRKGKSKSSNSNTSNMNGGGTTTSIVPVSVDSTGRVKHEAILGEKGSLHSQYKDLIPKQHTEHELQRPDDDELQETLDRTKNALEKIVNGKIKSSKSTNYVEVEKKSATYIKYTPSNQLGSNNGSALNSKIVRMVDVAQDPLEPPKYKIKKKIMEHGSPPAPVMHSPTRKLSVQDQQDWTIPPCVSNWKNPKGFAISIDKRLVSNGGGLQDVEINDKFAHFTQALYIAESNAREEVSARAELERKLAQKEKERKQDMLRKLAEDVRNERSGIIQQRYTRKDNSDSDNDNDNDSSSDEDKNKRTPPMNRRSRSRSTERIPSRNDNDDDDDRYRIKDNRDNRGRDNIDSRDNRDSRDSRDSRDSRDSRDSRDSRDNRDSRDSRDNRDNRDNRRRDDSNDRDRYSKRRSDSDSDSDSDSSDSEDERVRRERKEKLERDKIRMEKKRELEREYRLEASGKKSKFNRDQDRDISEKIALGQASIKRTEDSIYDQRLFNQSESLTSGFGNDDSYNVYSKPLFGGAVSNSIYRPKSNQEDNTSIQDVLSNSRFGKEGGSGSGGVPRPNKEFSGTDRSKDRTGPVAFEKEKKKSDDPFGFDDFSKKR

>P42522|MYOC_DICDI Myosin IC heavy chain - Dictyostelium discoideum (Slime mold).

MAQQKPEWGNQMKNEGLDDMTLLSKVSNDQILDNLKKRFEKDIIYTNIGDVLISVNPFKFIDGMYSDEVLQEYIGKSRIELPPHVFAVAEQTYRSMINEKENQCVIISGESGAGKTEAAKKIMQYIADVSGERGSSSNQKVEHVKSIILETNPLLEAFGNAKTLRNNNSSRFGKYFEIQFNQKNEPEGGKITNYLLEKSRVVFQLKGERNFHIFYQFCRGATPQEQQEFGIYGPENFAYLTKGDTLDIDGVDDVEEFALTRNAMNVIGIPANEQKQIFKLLAAILWIGNIDFKEQAGDKVTIADTSVLDFVSQLLDVPSHFLKTALEFRQMETRHGNQRGTQYNVPLNKTQAIAGRDALAKAIYDRLFNWLVDRINKEMDNPQKGLMIGVLDIYGFEVFDRNGFEQFCINYVNEKLQQIFIEFTLKMEQEEYVREGIKWEPIPFFDNKIVCELIEGKNPPGIFSILDDVCRAVHSQAEGADQKLLQSIAVCKSNPHFDTRGNAFCVKHYAGDVVYEGPGMIEKNKDTLLKDHLEILQMSANNFLVGLFPDVIDTDSKKLPSTAGFKIKSQAAELVATLMKSTPHYIRTIKPNDLKKPNILEGGRVLHQVKYLGLLDNIKVRRAGFAYRATFDRFFQRYYLLSDKTCYAGNNIWKGDALSACRAILASQNVDNTQYQIGKTKIFIRYPEMLFSLEETRERYWHDMASRIKNAYRNYKAFQFECSNRIKNAFRNYKLYRQRCAQTIQGYFRAWKQASPFFDLRMQNEQLFQGRKERNRFSMISVRKYFGDYLDVRSQSYFLDAMAEGRNEDVIFSSKSQVMVHPILSANKLSPRFLIVTKQAIYLIKLKQKKNLATYLLDRRVPLAEVTSFSLSSLADNLLVIHTSTQFDVAVTTEFKTELVALINKQKGTTLAVNFGQSIQYFKKKGSNNTVTFLKDEMHKEIFLKKNQFHIASGLPASTTVAKVRKNPSQVSTPSKPIAKPVAKPMVAKPSGGSVIMKKPAPAAPPSGPPVMKKPAPTAPGGAPMMKKPAPAPGGAPMMKKPAPVPGGPAPGGSAIMKPAGGVSKPLPSPTGAPMMKKPAPTAPGGPAPAGAPTPMMKKPAGQPMMKPIAKPQPTPMKKPAAPPPQQYIALYEYDAMQPDELTFKENDVINLIKKVDADWWQGELVRTKQIGMLPSNYVQQI

>Q95ZG4|SPC98_DICDI Spindle pole body component 98 - Dictyostelium discoideum (Slime mold).

MNSTFVQIPKLNINKQVKKKTDSITANTLLPKLMNDTSIQQPNQQQQQQQPQQPQQVTNGITILQNYQPQQPQPATTTTQQLNSANNTPTLVSTKKSVIGINEIPEHLLIRDIIYVFQGIDGTYIKYNKQSDSFKIDENTSNTLVNGEPAYISKPKRDLVYRLCEFGWLFKKVRLFITNNDFKKTGLTNQSFCSAINDELIELYRIIAILETQVYKKFDMVNYGGGGGGSGGSGSGSGLESPSSVSSGGTTTSTEIPFIDGDSLTLIRLFVWIQSPLKRLKVLGTCVDSITVDMKGGEILSKIDTLSKHGDQDIRILIHNIMFKICQPLFSMIRLWMFKGEINDPYQEFFIRQYESVQLEKTWKEKFAIVARLLPSFISLPLSKRILIIGKSINYMKQFCNNFKEDKNDRYYYYNQEDDDDDDEDHDDNDDDDENENQGEDDEIIERKLLIKESKIIKEKTKELNYINKEVLQEIIELVSRQSSERLLKIVLNRFKFMNHVKALKKYLLLGQGDFIQYLMDLIGEDLLKPTSQIQRHKLVGWMDTAIRNSNAQFEEQDIVNRLDIALLPERPGNIGWDIFSLDYHVDTPLNTILSPNDILRYKKIFHFMWGIKRVEYSLASIWRKIRSSTSLSILSPIGGDIHKSHLIMNEMVHFISNFQYYLMFEVLECSWKNLEKFIDQEATDLDQLIEAHHQYLQDICNKMFLSNSDSCYECFKKLLSIIIKFTLLQTKLINLSIAIQNEKNFNETHQAQVNKEFNSFRNHLNNLYQEYTTSFYKFQSEILKVKVNQDLNPISLQYMLDFNEYYEEKKDN

>Q01957|CPP1_ENTHI Cysteine proteinase 1 - Entamoeba histolytica.

MFTFILMFYIGYGIDFNTWVANNNKHFTAVESLRRRAIFNMNARIVAENNRKETFKLSVDGPFAAMTNEEYNSLLKLKRSGEEKGEVRYLNIQAPKAVDWRKKGKVTPIRDQGNCGSCYTFGSIAALEGRLLIEKGGDSETLDLSEEHMVQCTREDGNNGCNGGLGSNVYNYIMENGIAKESDYPYTGSDSTCRSDVKAFAKIKSYNRVARNNEVELKAAISQGLVDVSIDASSVQFQLYKSGAYTDTQCKNNYFALNHEVCAVGYGVVDGKECWIVRNSWGTGWGEKGYINMVIEGNTCGVATDPLYPTGVEYL

>O15736|TIPD_DICDI Protein tipD - Dictyostelium discoideum (Slime mold).

MFSSQNNSYMMMMGGGGIGNINNNQFYSPIISTSAQSFNSIVEWKRDIIRQLNDRNQNQTNNYSEFMRIYTDLLKRERTLNDRTLLYEKEIVSLRNEKKTQQQPPSGSSKMDSSSSSSSSNRVSGMGSTIEEMEQKLFKLQEDLTNSYKRNADNASSILLLNDKNKDLQNELMSKEIEIERIRSTIQQDLDSIKRLEMVVIEKENVSQIIRDELSSLQTEFLHNESKVVKLEQENSSLVERWLRKKNEEASKMNEANDFYQKMVEQRDSTPAKAAVQLSESISNLVVKLPDANDVPIPIVLERGVFSSEAMLPSKAKKRWTGHNSEIYCMAFNSIGNLLATGGGDKCVKVWDVISGQQKSTLLGASQSIVSVSFSPNDESILGTSNDNSARLWNTELGRSRHTLTGHIGKVYTGKFINSNRVVTGSHDRTIKLWDLQKGYCTRTIFCFSSCNDLVILGGSGTHLASGHVDHSVRFWDSNAGEPTQVLSSIHEGQITSITNSPTNTNQILTNSRDHTLKIIDIRTFDTIRTFKDPEYRNGLNWTKASWSPDGRYIASGSIDGSICIWDATNGKTVKVLTKVHNNGSSVCCCSWSPLANIFISADKDKNIIQWE

>P02895|GBP_PLAFG Glycophorin-binding protein - Plasmodium falciparum (isolate FCR-3 / Gambia).

MRLSKVSDIKSTGVSNYKNFNSKNSSKYSLMEVSKKNEKKNSLGAFHSKKILLIFGIIYVVLLNAYICGDKYEKAVDYGFRESRILAEGEDTCARKEKTTLRKSKQKTSTRTVATQTKKDEENKSVVTEEQKVESDSEKQKRTKKVVKKQINIGDTENQKEGKNVKKVIKKEKKKEESGKPEENKHANEASKKKEPKASKVSQKPSTSTRSNNEVKIRAASNQETLTSADPEGQIMREYAADPEYRKHLEIFYKILTNTDPNDEVERRNADNKEDLTSADPEGQIMREYASDPEYRKHLEIFYKILTNTDPNDDVERRNADNKEDLTSADPEGQIMREYAADPEYRKHLEVFHKILTNTDPNDEVERRNADNKEDLTSADPEGQIMREYAADPEYRKHLEIFHKILTNTDPNDEVERRNADNKEDLTSADPEGQIMREYAADPEYRKHLEVFHKILTNTDPNDEVERRNADNKELTSSDPEGQIMREYAADPEYRKHLEIFHKILTNTDPNDEVERRNADNKEDLTSADPEGQIMREYAADPEYRKHLEIFYKILTNTDPNDEVERRNADNKEELTSSDPEGQIMREYAADPEYRKHLEIFHKILTNTDPNDEVERRNADNKEDLTSADPEGQIMREYAADPEYRKHLEIFYKILTNTDPNDEVERRNADNKEDLTSADPEGQIMREYASDPEYRKHLEIFYKILTNTDPNDDVERRNADNKEDLTSADPEGQIMREYAADPEYRKHLEIFHKILTNTDPNDEVERQNADNNEA

>Q8I3H7|TIP_PLAF7 T-cell immunomodulatory protein homolog - Plasmodium falciparum (isolate 3D7).

MYNFLSCKKKSIILQVLLIICTYNILLNFVNIFVNNNEKNHKNKYENRIKSFYVEAYNWNFLEKWKSINTNEKLEYKINYNIGLNIDAEIGDFGDYNSDVKTDLILFKYDKDKLLSTIFIYVFSVKENKFIYHTEVSFEGKIMNVTAIDLNFDGALDVLVLFKDNKDSSKSNKYYVAAFLQNDNDQLEEIWNSKKKEQNDESITDNEEDNIYYTNIHPLICDINNDGLPDIIGQQSGGPDGFFRFIWINTRNGFKSFLWKNINIFKYSELDEITNPNSSAIVDINGDCKSDLVFTVYNSYEKRIGLEIWLNKIIDGKSFYVKYSQDYLLPPNSLQVLFGDFNGDGSIDLVVPTCVKSSFCNYCCVSDDKIYFIPNIQKKICDSSWKKPDETKCRPASNLCSESDFEFQQNLTDDFISVVDTSGLHLSGNADYPYYLSVGDIDDDGYLDLLITLKNDKGQKYVRIYKNELKIHYEENSLEVRGFYNFYQFVTSPEESVTDVYNAAFFDIFENGVLDILIFGKYITSNKKTKYAAVGFIRNNETDSLFLKSTALNGICVNDCYKEKDKITTKTLGGNAHGPTFKITVIDVNGVKSSRIGVQKSQSAHFPLQLPYVLFGLGRTSNYVEEFYVGMPTHEQKYYNMWVSIIPNSHIIVIPYPLNNSNKWQIQLSVNPSKKFYSILYITLICLSVIGVLIFILDRKEKVEDSKEELGFKSHFVIG

>P24639|ANXA7_DICDI Annexin A7 - Dictyostelium discoideum (Slime mold).

MSYPPNQGYPPQSNSPQPGQYGAPQQGYPPQQGYPPQQGYPPQQGYPPQQGYPPQQGYPPQQGYPPQQGYPPQQGYPPQQGYPPQQGYPPQQGYPPQQGYPPQQGYPPQQGYPPQQGYPPQGYPPQQGYPPVGVPVGVPVGFAPGMVVGYHQGYFVGTITHDCKHDAEVLRKAMKGIGTNESDLIKVLANRNWAEREQIKREFSAKYSKDLIQDIKSETSGNFEKCLVALLTEPAHFDVEQIHSACAGAGTNENTIIEILVTRSNVQMEYIKQIFKNKHGKSLKDRLESEASGDFKKLLEKLTEPRDESPVINPMQVSKDAEDLYKAGEGKIGTDEKEFIKILTSRSLPHIAAVASEYIKHHKKHSLIKAIDSEFSGSIKTGLIAIVTYALNPYGYFAEILNKSMKGAGTNDNKLIRTVVTQMHNMPQIKTAYSTLFKNSLAHDIQADCSGDFKKLLLDIIS

>O96436|1433_EIMTE 14-3-3 protein - Eimeria tenella.

MIEDIKTLREEHVYRAKLAEQAERYDEMAEAMKNLVENCLDQNNSPPGAKGDELTVEERNLLSVAYKNAVGARRASWRIISSVEQKEANRNHMANKALAASYRQKVENELNKICQEILTLLTDKLLPRTTDSESRVFYFKMKGDYYRYISEFSNEEGKKASAEQAEESYKRATDTAEAELPSTHPIRLGLALNYSVFYYEILNQPQKACEMAKLAFDDAITEFDSVSEDSYKDSTLIMQLLRDNLTLWTSDLQTQEQQQQPVGEGAEAPKVEATEQQ

>P18240|G6PI_PLAFA Glucose-6-phosphate isomerase - Plasmodium falciparum.

MNMEITNLKSYKELVTLSAEEKTKDLKDYLNDKNRSESLIKKFKNFYMDLSRQRYSEKTLNKLVEYAEEVELKKKVEKTFMGEKVNMTENRSVLHTALRIPIEKINTHKIIIDNKNVLEDVHGVLKKIEKYSDDIRNGVIKTCKNTKFKNVICIGIGGSYLGTEFVYEAMKYYYYNMELNKNEKDQVNNFNNNYDQDNVFNVRFLANVDPNDVNRAIQNLDQYDTLVIIISKTFTTAETMLNARSIKKWLSLKIKDDENLSKHMVAVSTNLKLTDEFGISRDNVFEFWDWVGGRFSVTSSVGILPLSIAFGYKNMRNFLNGCHDMDEHFLHADLKENIPVLLALTSFYNSHFFDYKNVAILPYFQNLLKFSAHIQQLSMESNGKSVDRNNQPIHYNTCQVYFGEPGTNGQHSFYQLIHQGQVIPVELIGFKHSHFPIKFDKEVVSNHDELMTNFFAQADALAIGKTYEQVKEENEKNKMSPELLTHKVFNGNRPSTLLLFDELNFYTCGLLLSLYESRIVAEGFLLNINSFDQWGVELGKVLAKEVRNYFNDTRNQKKSDNTYNFNESTKNFIKLLLVQIKKKKKINTNLK

>P25805|CYSP_PLAFA Trophozoite cysteine proteinase - Plasmodium falciparum.

MVAIKEMKELAFARPSLVETLNKKKKFLKKKEKRTFVLSIYAFITFIIFCIGILYFTNKSSAHNNNNNKNEHSLKKEEIELLRVLLEKYKKQKDGILNESSNEEDEEKYTLNSETYNNKNNVSNIKNDSIKSKKEEYINLERILLEKYKKFINENNEENRKELSNILHKLLEINKLILREEKDDKKVYLINDNYDEKGALEIGMNEEMKYKKEDPINNIKYASKFFKFMKEHNKVYKNIDEQMRKFEIFKINYISIKNHNKLNKNAMYKKKVNQFSDYSEEELKEYFKTLLHVPNHMIEKYSKPFENHLKDNILISEFYTNGKRNEKDIFSKVPEILDYREKGIVHEPKDQGLCGSCWAFASVGNIESVFAKKNKNILSFSEQEVVDCSKDNFGCDGGHPFYSFLYVLQNELCLGDEYKYKAKDDMFCLNYRCKRKVSLSSIGAVKENQLILALNEVGPLSVNVGVNNDFVAYSEGVYNGTCSEELNHSVLLVGYGQVEKTKLNYNNKIQTYNTKENSNQPDDNIIYYWIIKNSWSKKWGENGFMRLSRNKNGDNVFCGIGEEVFYPIL

>P54638|ARGE_DICDI Acetylornithine deacetylase - Dictyostelium discoideum (Slime mold).

MTKPVASYELDEKRFLTLLGKLIGETENLQNRPPALIPIEDNAGRHVIEALTPYLKANGGVLELEQVHCDPVNYPKRGNIIIEYPGTSKGTSSPKTISFVGSHLDVVPADKTAWDRNPFQLIIEGDKLYGRGTTDCLGHVALLTDLFIQLATEKPALKHSIFAVFIVSEENDEIPGIGVDALDHSGKMNPCKNGPVYWVDSADSQPTIGTGGAQTWNLTAHGKNMHSAMPYRTVNSVELVNEALAEIQRRFYIDFKPHPKEAEYKFDCSSTMKPTLWKPIAGSYNTIPGESTICGDIRLTPFYDMKEMRAKVEGYIKDINANITELRNRGPYSKYDVPASEGVEPVKGSVSIEWLGEASAGVACKLDSDGYKALGKATSEILGSLTPVATCGTLPLVRDLQDSGFDIQITGFGKEETYHADNEYALLSDFKNAIKILSRTIDLLEKN

>P51058|PPCK_TRYCR Phosphoenolpyruvate carboxykinase [ATP], glycosomal - Trypanosoma cruzi.

MPPTIHRNLLSPELVQWALKIEKDSRLTARGALAVMSYAKTGRSPLDKRIVDTDDVRENVDWGKVNMKLSEESFARVRKIAKEFLDTREHLFVVDCFAGHDERYRLKVRVFTTRPYHALFMRDMLIVPTPEELATFGEPDYVIYNAGECKADPSIPGLTSTTCVALNFKTREQVILGTEYAGEMKKGILTVMFELMPQMNHLCMHASANVGKQGDVTVFFGLSGTGKTTLSADPHRNLIGDDEHVWTDRGVFNIEGGCYAKAIGLNPKTEKDIYDAVRFGAVAENCVLDKRTGEIDFYDESICKNTRVAYPLSHIEGALSKAIAGHPKNVIFLTNDAFGVMPPVARLTSAQAMFWFVMGYTANVPGVEAGGTRTARPIFSSCFGGPFLVRHATFYGEQLAEKMQKHNSRVWLLNTGYAGGRADRGAKRMPLRVTRAIIDAIHDGTLDRTEYEEYPGWACTSRSTSPKCRSIC

>P13475|3F_DICDI Protein 3F - Dictyostelium discoideum (Slime mold).

MKLLSKLILTLALATYASASESFRYINWDNPPHDVTFYEGDVLQFTTNEGRNSTITLISDTENGDKSFDGVLNEDQRSFVQKALPPGRYTFKDLNSGSKSIIRVKESKELAKEVRPIDRLKDNADAANTENAQKSPNTQSTQKGSPKSDAKEASPKTDAKEASPKSDAKEASPKTDTKQGSSPKTDTKSSTQKPSSSSDSSKAKAEANTAANNEEAEHVEKGASNTLKASLSIISAACVLSLGYLL

>P10901|FUCO_DICDI Alpha-L-fucosidase - Dictyostelium discoideum (Slime mold).

MKMIIIFFILLILNLIKSQQYGPTWDQINSRPLPGWYDDVKFGIFIHFGIYSVPAFANGGYAEWYWWTLKNPSSDGGATQRYHEKEFGANFTYQDFVSRFDCRLFDANEWASIIEKSGAKYVVLTSKHHEGYTLWNSEQSWNWNSVETGPGIDIVGELTKSVKNMGLHMGLYHSLFEWFNPLYLADAETGKNPTTQVYVDEILMKQLKDIVTTYEPELIWADGDWMQLSNYWKSTEFLSWLYTNSSVKDTVIVNDRWGSECRDKNGGFYTGADHFNPYKLQSHKWENCATIGYSYGYDEYEQATDYQNATELIIDLVTTVACGGNFLLDVGPDAQGTIPNNMVDRLLEIGNWLSINSESIYGSSPWRVQNMTFNIWYTTNTTNGNVYAFVFELPDDGVLILSDPIGNNKTEATLLGLKGEKGVEVSLPIESTKPGITLNIPMVAPQDYPPYVYVFRLTDVE

>P51136|GSK3_DICDI Glycogen synthase kinase-3 - Dictyostelium discoideum (Slime mold).

MSSKDQILEKDKKETDDNGNKKTTTTTSSSSSSSSSSKPRSNKFDKVIIKSNGVCYITEGVIGNGSFGVVTQAIVADTKEVVAIKKVLQDQRYKNRELQIMKMLNHINIVSLKNSFYTSDNDEVYLNLVLEYVPDTVYRVSRHYSMSKQPVPNIFVKLYIYQLCRSINYIHSLGICHRDIKPQNLLLDTSTSTLKLCDFGSAKILIKGETNVSYICSRHYRAPELIFGSTNYTTTIDVWSLGCVLAELLLGQPLFPGENGIDQLVEIIKVLGTPTKEQIHAMNPYYTSFKFPEIKANPWPRVFKAKDVPAESIDLISKILLYDPSSRLKPVEICAHPFFDELRDPKTCLPDGKPLPPLFNFTIAEQTSIGPKLAKTLIPSHAMNQIELPSPLFPNLAISSSNQSSSSNSNANVSSNLNSHSASPSTTSSSSSTPNSIPVQSPSTTNTTSSTTNNTTTTTTTTTTSNH

>P02889|PSMD8_DICDI Probable 26S proteasome non-ATPase regulatory subunit 8 - Dictyostelium discoideum (Slime mold).

MDFSSIEQNLNNFKKLVAGNSDKAQITPVLVQLKLAATIHLEKPTSLSNVSDKVKKDLVLAREILELISLYSIKIKDIDSFERTFNQLKTYYYDYKSIIAPSTLEYQIIGLNLMRLLAKHKTSEFHSEIELIEFNNLDNSFIKFPLLVEKSITEGSYNKIIQSRSGVPSEYYQVFLDILADSIKEDIANCSEKSFKTLSLKDAEKVLLFNDNNQFQQYIKERNWKVQGDVIQFGNNDNQTVEIPSLQLIHQTLHYAKELERIV

>Q08210|PYRD_PLAF7 Dihydroorotate dehydrogenase homolog, mitochondrial - Plasmodium falciparum (isolate 3D7).

MISKLKPQFMFLPKKHILSYCRKDVLNLFEQKFYYTSKRKESNNMKNESLLRLINYNRYYNKIDSNNYYNGGKILSNDRQYIYSPLCEYKKKINDISSYVSVPFKINIRNLGTSNFVNNKKDVLDNDYIYENIKKEKSKHKKIIFLLFVSLFGLYGFFESYNPEFFLYDIFLKFCLKYIDGEICHDLFLLLGKYNILPYDTSNDSIYACTNIKHLDFINPFGVAAGFDKNGVCIDSILKLGFSFIEIGTITPRGQTGNAKPRIFRDVESRSIINSCGFNNMGCDKVTENLILFRKRQEEDKLLSKHIVGVSIGKNKDTVNIVDDLKYCINKIGRYADYIAINVSSPNTPGLRDNQEAGKLKNIILSVKEEIDNLEKNNIMNDESTYNEDNKIVEKKNNFNKNNSHMMKDAKDNFLWFNTTKKKPLVFVKLAPDLNQEQKKEIADVLLETNIDGMIISNTTTQINDIKSFENKKGGVSGAKLKDISTKFICEMYNYTNKQIPIIASGGIFSGLDALEKIEAGASVCQLYSCLVFNGMKSAVQIKRELNHLLYQRGYYNLKEAIGRKHSKS

>P36412|RB11A_DICDI Ras-related protein Rab-11A - Dictyostelium discoideum (Slime mold).

MTSKGSQEEYDYLYKIVLIGDSGVGKSNLLSRFTRNEFSLETKSTIGVEFATRTIQTEGKTIKAQVWDTAGQERYRAITSAYYRGAVGALLVYDIAKQATYKSVERWILELRENADRNIEIMLVGNKSDLRHLREVSTDEAKEFSEKHKLTFIETSALDSSNVELAFQNILTQIYHIMSRPSHSTGPQTTIDSNTETIILPTTSEPPAAKSGCC

>P54672|AP2M_DICDI AP-2 complex subunit mu - Dictyostelium discoideum (Slime mold).

MISALFLMNGKGEVLISRIYRDDISRGVANAFRLEVIGSQETRSPVKLIGSTSFMYIKVGNIYIVGVSRQNVNACMVFEVLHQLVDIFKSYFDNLDEDSIRNNFVLVYELLDEILDFGYPQNCSTDVLKLYITQGQGKLKSLDKLKQDKISKITIQATGTTPWRTPDIKYKRNELYIDVVESVNLLMSAEGNILRADVSGQVMMKCFLSGMPECKFGMNDKVIMDREKSTNGGSAARSGARRANGIEIDDITFHQCVRLGKFDSDRTVSFIPPDGEFELMRYRTTEHINLPFKVIPIVREMGRTRLECSVTVKSNFSSKMFGANVKVIIPTPKNTAVCKIVVAAGKAKYMPEQDAIIWRIRRFPGDTEFTLRAEVELMASVNLDKKAWSRPPISMEFQVTMFTASGFSVRFLKVVEKSNYTPIKWVRYLTKAGTYQNRI
