# Supplementary material for: CyclinPred: A SVM-Based Method for Predicting Cyclin Protein Sequences
Source: PLoS One. 2008 Jul 2;3(7):e2605. doi: 10.1371/journal.pone.0002605 (PMC2435623; doi:10.1371/journal.pone.0002605)
Supplement: Dataset S3 — Independent sequences (Independent data set) used for blind-test performance. (0.05 MB DOC) [file pone.0002605.s005.doc]

**Data set S3. Independent sequences (Independent data set) used for blind-test performance**

>O15995|CCNE_HEMPU G1/S-specific cyclin-E - Hemicentrotus pulcherrimus (Sea urchin).

MSRRSGRLQSRQDNQPLTECISDENNLPMCTRKRKTREQDTTGVSKAEEVQRRRQQFTIENRWVPISESSSIETSLLVPMQTKEPSTPSEELMDTANWVTFRNLFPAHVSDRASPIPLLHWDDLPEVWTIMTRKEALCPRKHDCLKSHPSLGERMRAILLDWLIEVCEVYRLHRESFYLAADFVDRYLAAKENVPKTKLQLIGITSLFVAAKLEEIYPPKLHEFAYVTDGACTDDQILDQELIMLMTLNWDLTPITVNTWLNAFMQICNAEEIANRKTNFHFPSYSSTEFVQVAQLLDVCTLDIGSMDFDYSILAASALYHVTNEEVTLSVTGLKWDDIAACVQWMSTFAMTIREVGVAQLKNFKNIYAGDAHNIQTHCSSLELLDKSHEKQRLLREASCCSPVQVPGVLTPPQSDKKSKKGVL

>O60563|CCNT1_HUMAN Cyclin-T1 - Homo sapiens (Human).

MEGERKNNNKRWYFTREQLENSPSRRFGVDPDKELSYRQQAANLLQDMGQRLNVSQLTINTAIVYMHRFYMIQSFTQFPGNSVAPAALFLAAKVEEQPKKLEHVIKVAHTCLHPQESLPDTRSEAYLQQVQDLVILESIILQTLGFELTIDHPHTHVVKCTQLVRASKDLAQTSYFMATNSLHLTTFSLQYTPPVVACVCIHLACKWSNWEIPVSTDGKHWWEYVDATVTLELLDELTHEFLQILEKTPNRLKRIWNWRACEAAKKTKADDRGTDEKTSEQTILNMISQSSSDTTIAGLMSMSTSTTSAVPSLPVSEESSSNLTSVEMLPGKRWLSSQPSFKLEPTQGHRTSENLALTGVDHSLPQDGSNAFISQKQNSKSVPSAKVSLKEYRAKHAEELAAQKRQLENMEANVKSQYAYAAQNLLSHHDSHSSVILKMPIEGSENPERPFLEKADKTALKMRIPVAGGDKAASSKPEEIKMRIKVHAAADKHNSVEDSVTKSREHKEKHKTHPSNHHHHHNHHSHKHSHSQLPVGTGNKRPGDPKHSSQTSNLAHKTYSLSSSFSSSSSTRKRGPSEETGGAVFDHPAKIAKSTKSSSLNFSFPSLPTMGQMPGHSSDTSGLSFSQPSCKTRVPHSKLDKGPTGANGHNTTQTIDYQDTVNMLHSLLSAQGVQPTQPTAFEFVRPYSDYLNPRSGGISSRSGNTDKPRPPPLPSEPPPPLPPLPK

>O60583|CCNT2_HUMAN Cyclin-T2 - Homo sapiens (Human).

MASGRGASSRWFFTREQLENTPSRRCGVEADKELSCRQQAANLIQEMGQRLNVSQLTINTAIVYMHRFYMHHSFTKFNKNIISSTALFLAAKVEEQARKLEHVIKVAHACLHPLEPLLDTKCDAYLQQTQELVILETIMLQTLGFEITIEHPHTDVVKCTQLVRASKDLAQTSYFMATNSLHLTTFCLQYKPTVIACVCIHLACKWSNWEIPVSTDGKHWWEYVDPTVTLELLDELTHEFLQILEKTPNRLKKIRNWRANQAARKPKVDGQVSETPLLGSSLVQNSILVDSVTGVPTNPSFQKPSTSAFPAPVPLNSGNISVQDSHTSDNLSMLATGMPSTSYGLSSHQEWPQHQDSARTEQLYSQKQETSLSGSQYNINFQQGPSISLHSGLHHRPDKISDHSSVKQEYTHKAGSSKHHGPISTTPGIIPQKMSLDKYREKRKLETLDLDVRDHYIAAQVEQQHKQGQSQAASSSSVTSPIKMKIPIANTEKYMADKKEKSGSLKLRIPIPPTDKSASKEELKMKIKVSSSERHSSSDEGSGKSKHSSPHISRDHKEKHKEHPSSRHHTSSHKHSHSHSGSSSGGSKHSADGIPPTVLRSPVGLSSDGISSSSSSSRKRLHVNDASHNHHSKMSKSSKSSGSSSSSSSSVKQYISSHNSVFNHPLPPPPPVTYQVGYGHLSTLVKLDKKPVETNGPDANHEYSTSSQHMDYKDTFDMLDSLLSAQGMNM

>O75909|CCNK_HUMAN Cyclin-K - Homo sapiens (Human).

MKENKENSSPSVTSANLDHTKPCWYWDKKDLAHTPSQLEGLDPATEARYRREGARFIFDVGTRLGLHYDTLATGIIYFHRFYMFHSFKQFPRYVTGACCLFLAGKVEETPKKCKDIIKTARSLLNDVQFGQFGDDPKEEVMVLERILLQTIKFDLQVEHPYQFLLKYAKQLKGDKNKIQKLVQMAWTFVNDSLCTTLSLQWEPEIIAVAVMYLAGRLCKFEIQEWTSKPMYRRWWEQFVQDVPVDVLEDICHQILDLYSQGKQQMPHHTPHQLQQPPSLQPTPQVPQVQQSQPSQSSEPSQPQQKDPQQPAQQQQPAQQPKKPSPQPSSPRQVKRAVVVSPKEENKAAEPAPSQHLW

>O88874|CCNK_MOUSE Cyclin-K - Mus musculus (Mouse).

MKENKENSSPSVTSANLDHTKPCWYWDKKDLAHTPSQLEGLDPATEARYRREGARFIFDVGTRLGLHYDTLANGIIYFHRFYMFHSFKQFPRYVTGACCLFLAGKVEETPKKCKDIIKTARSLLNDVQFGQFGDDPKEEVMVLERILLQTIKFDLQVEHPYQFLLKYAKQLKGDKNKIQKLVQMAWTFVNDSLCTTLSLQWEPEIIAVAVMYLAGRLCKFEIQEWTSKPMYRRWWEQFVQDVPVDVLEDICHQILDLYSQGKQQMPHHTPHQLQQPPSLQPTPQVPQGPQSQPSQGSEAAQPPQKDSQQSAQQQQQQAQQPKKPSPQPSPPRQAKRAVVVSPKEENKATEPPPPPKIPKLEATHPPLPPAHPPPDRKPPLAPALGEAEATGPVETSDLPKVQIPPPAHPAPVHQPPPLPHRPPPPPPSSYMTGMSTTSSYMSGEGYQSLQSMMKTEGPSYGALPPASFPPPTIPPPTPGYPPPPPTYNPNFPPPPPRLPPTHAVPPHPPPGLGLPPASYPPPAVPPGGQPPVPPPIPPPGMPPVGGLGRAAWMR

>O94503|SRB11_SCHPO RNA polymerase II holoenzyme cyclin-like subunit - Schizosaccharomyces pombe (Fission yeast).

MAANYWASSQLTQLFLSTDLESLEPTCLSKDTIYQWKVVQTFGDRLRLRQRVLATAIVLLRRYMLKKNEEKGFSLEALVATCIYLSCKVEECPVHIRTICNEANDLWSLKVKLSRSNISEIEFEIISVLDAFLIVHHPYTSLEQAFHDGIINQKQLEFAWSIVNDSYASSLCLMAHPHQLAYAALLISCCNDENTIPKLLDLIKSTDAFKVILCVQRIISIYYFEDIE

>P0C242|UDG2_MOUSE Uracil-DNA glycosylase 2 - Mus musculus (Mouse).

MVTPCPASPGSPAAGAGRRDSHQNLRAPVKKSRRPCLRRKKPLRPLNACSLPGDSGVCDLFESPSSSSDGADSPAVSAARDCSSLLNPAQPLTALDLQTFREYGQSCYDFRKAQENLFHPRESLARQPQVTAESRCKLLSWLLQVHRQFGLSFESLCLTVNTLDRFLLTTPVAADCFQLLGVTCLLIACKQVEVHPPRLKQLLALCGGAFSRQQLCNLECIVLHKLHFSLGAPTINFFLEHFTQWRMEAGQAEVTEALEAQTLARGVAELSLTDYAFTTYTPSLMAICCLALADGLLQHQHEMDLRLGEHPEATLQDCLGKLQTLVSINSSSLPRILPPQIWERCSLPQSWQ

>P22674|UDG2_HUMAN Uracil-DNA glycosylase 2 - Homo sapiens (Human).

MVTPCPTSPSSPAARAGRRDNDQNLRAPVKKSRRPRLRRKQPLHPLNPCPLPGDSGICDLFESPSSGSDGAESPSAARGGSPLPGPAQPVAQLDLQTFRDYGQSCYAFRKAQESHFHPREALARQPQVTAESRCKLLSWLIPVHRQFGLSFESLCLTVNTLDRFLTTTPVAADCFQLLGVTSLLIACKQVEVHPPRVKQLLALCCGAFSRQQLCNLECIVLHKLHFTLGAPTISFFLEHFTHARVEAGQAEASEALEAQALARGVAELSLADYAFTSYSPSLLAICCLALADRMLRVSRPVDLRLGDHPEAALEDCMGKLQLLVAINSTSLTHMLPVQICEKCSLPPSSK

>P24863|CCNC_HUMAN Cyclin-C - Homo sapiens (Human).

MVAPRPLRRVVLFYQGKLCSMAGNFWQSSHYLQWILDKQDLLKERQKDLKFLSEEEYWKLQIFFTNVIQALGEHLKLRQQVIATATVYFKRFYARYSLKSIDPVLMAPTCVFLASKVEEFGVVSNTRLIAAATSVLKTRFSYAFPKEFPYRMNHILECEFYLLELMDCCLIVYHPYRPLLQYVQDMGQEDMLLPLAWRIVNDTYRTDLCLLYPPFMIALACLHVACVVQQKDARQWFAELSVDMEKILEIIRVILKLYEQWKNFDERKEMATILSKMPKPKPPPNSEGEQGPNGSQNSSYSQS

>P24867|PCL1_YEAST G1/S-specific cyclin PCL1 - Saccharomyces cerevisiae (Baker's yeast).

MCEYSKALHILLKSPVTDDIIKFLTDTTLRVVPSSNYPTPPGSPGEKHLTRLPSLMTFITRLVRYTNVYTPTLLTAACYLNKLKRILPRDATGLPSTIHRIFLACLILSAKFHNDSSPLNKHWARYTDGLFTLEDINLMERQLLQLLNWDLRVNTEDLILDLQPLLEPIKQDLARSSDQRKRINMMMSMNRRTCAGTSPIRSNNRFKLYEKQRNVSIASDLSSATLVDSCNDLRRLKDVTNIANNTVANTNYVRTVEKWNDNVNRQSWDLEQIMSQHGF

>P25008|CCNC_DROME G1/S-specific cyclin-C - Drosophila melanogaster (Fruit fly).

MAGNFWQSSHSQQWILDKPDLLRERQHDLLALNEDEYQKVFIFFANVIQVLGEQLKLRQQVIATATVYFKRFYARNSLKNIDPLLLAPTCILLASKVEEFGVISNSRLISICQSAIKTKFSYAYAQEFPYRTNHILECEFYLLENLDCCLIVYQPYRPLLQLVQDMGQEDQLLTLSWRIVNDSLRTDVCLLYPPYQIAIACLQIACVILQKDATKQWFAELNVDLDKVQEIVRAIVNLYELWKDWKEKDEIQMLLSKIPKPKPPPQR

>P25009|PUC1_SCHPO Cyclin puc1 - Schizosaccharomyces pombe (Fission yeast).

MLVSSNEEQLTAHTPTSSSSIEPKILAACSYSLSVGPCSLAVSPKGVNSKSPSLKNETAFVVDSVSTLSAESSALLYNTQSSLLTGLSMNGYLGEYQEDIIHHLITREKNFLLNVHLSNQQPELRWSMRPALVNFIVEIHNGFDLSIDTLPLSISLMDSYVSRRVVYCKHIQLVACVCLWIASKFHETEDRVPLLQELKLACKNIYAEDLFIRMERHILDTLDWDISIPTPASYIPVLDPIFFLVLDASMFVPNLFKFPASKIACSVMNIVNEHVGSFLLTHPSMESYRKDDNFLWPEDLDTVTSYMENMSKRYANEECTDLLFSSLGRISSILAKKYPEQCAMAAWCNMTEKDTERTL

>P25693|PCL2_YEAST G1/S-specific cyclin PCL2 - Saccharomyces cerevisiae (Baker's yeast).

MSNYEALLKFNRKAVSKEMVQYLASTTASIIKIKKTNSMIDIALPAPPLTKFINRLIKHSNVQTPTLMATSVYLAKLRSIIPSNVYGIETTRHRIFLGCLILAAKTLNDSSPLNKHWAEYTDGLLILREVNTIERELLEYFDWDVTISTDDLITCLSPFLKPIKEEQLYKSQRDCRTLKNFSAQEKDIVNKTSISHSRSSSNMSIPSLASTSTLSTLESRRSNLSNYSNRIRTLPELHESNNISDKFSPRTYNIDSKHDNKENRPIPTIKPFNFSKARPVILKTGLNKQIIKEDTKVKKSNWSNYFKS

>P34624|YOJ1_CAEEL Hypothetical protein ZK353.1 in chromosome III - Caenorhabditis elegans.

MGNSSCCLRTRSSSGEDKSYNNDGQYIRTNQVEFQYVNQVFPRDETSTNFLPHISEREVTEGYEEDPSTNPTARPTFMERSKSEMKLKDNRRSCYMLDALAAGGHHPGILPRSLRKSSSCSTIYIDDSTVSQPHLKNTIKCISLAIYYHISNRKNRGHERLMEIFEERLHPIFRDPIPPEQMTRDPDHRNIYRFVRNLFSSAQLTAECAIITLVYIERLLNYAEMDLCPSNWRRVVLGSIMLASKVWDDQAVWNVDYCQILRDTNVDDMNELERRFLECLDFNIEVPSSVYAKYYFDLRTLALANDLQLPIQPLYKERAQRLEALSRVFEDKIQSSSLPKRARSAEHLVFEHPAVLS@

>P35190|CLG1_YEAST Cyclin-like protein CLG1 - Saccharomyces cerevisiae (Baker's yeast).

MANTFKYYPETMGNSSGYPISLPFPKGSATSAVNVARQLPKYLGHVPSQSVHTQLPSMASLGYFNQPSSTYYAPPAPLQQHQQPPILPPPGLMYTSNNNSNVIPPPVQMIRDGQQQPQQSNQVNGGVSENLDYDISIMSKFIMENAFVAFNANYSTDDQTTDLFFKGISSVLNATRLPSATIFLAIDYLFKYINKLSNGIHSIGGNSINIIYQNTMIAFILANKFNDDKTFTNNSWSQATGILINVINDFERQWLRIFNWELYDSAFLYFEFVKNFEIFKQNQLKPAVAVPTLLSPIVNVGDTRNVNFNLKPTSTNNLLSPVSNYETPMLMPHNMFSSPSYQSNSRSEFSSMNGYYNYYNYNQPRLNYYQQFPNIYSSPISETQFDYDFYNFSSQQQQQQQKQHSLLPAAPQLPPPHVHNQSYGHHLGWKSMDDTINHSRFERNYFPYSAVY

>P36613|CGM2_SCHPO Cyclin mcs2 - Schizosaccharomyces pombe (Fission yeast).

MSADKFRDSTHYRDWIFTEEDLSKTRAKVNEKFTNIVRERMLEELSLQNKEASLEVLPPTLTVEEELELVNYYSFQLNALSSALSLPTHIRSTAILFFKRFYLINSVMEYSPKIISFTSLFLATKCNDHYISIEQFCKNMPKTTPEEVLEYEFNVCQSLKWDLYVWLPFRPLQGFLLDCQTVLPKVAVEKFYECHDLSKKFLIETLHSDIYFLHSPSIIALGAIYHTNPTICLQYIEAKKIPELQPLIISISANLKATKKFKIEKKKAQDYGRKLYFCMNPLRNKSSALYLKRKAEEESTNNNKWAKKFSTSSNVLDKNPFE

>P38794|PCL5_YEAST G1/S-specific cyclin PCL5 - Saccharomyces cerevisiae (Baker's yeast).

MDGNHRFTPDSKEFNTVVKSKESSTGRNPYQTPPLEHNGTHHQTNYSRKKTNLAIIISNFLSEISRPLSNGKINNSTHNILKFLNEVLKRSKCSKENAVLATFYFQKIHQSRGVRDESSLPEFSHCSRRIFLCCLILSHKFLNDNTYSMKNWQIISGLHAKDLSLMERWCLGKLNYELAIPYDEFLLWETNTLMKAKLRVGTPANAPVKRPRESDNDYDANSWKQIKSC

>P39947|CCNC_RAT Cyclin-C - Rattus norvegicus (Rat).

MVAPRPLCRLVLLYQAKQYSMAGNFWQSSHYLQWILDKERQKDLKFLSEEEYWKLQIFFTNVIQALGEHLKLRQQVIATATVYFKRFYARYSLKSIDPVLMAPTCVFLASKVEEFGVVSNTSLIAATTSVLKTRFSYASPKEFPYRMNHILECEFYLLELMDCCLIVYHPYRPLLQYVQDMGQEDVLLPLAWRIVNDTYRTDLCLLYPPFMIALACLHVACVVQQKDARQWFAELSVDMEKILEIIRVILKLYEQWKNFDERKEMATILSKMPKPKPPPNSEGEQGPNGSQNSSYSQS

>P39950|CCNG1_RAT Cyclin-G1 - Rattus norvegicus (Rat).

MIEVLTTDSQKLLHQLNTLLEQESRCQPKVCGLKLIESAHDNGLRMTARLRDFEVKDLLSLTQFFGFDTETFSLAVNLLDRFLSKMKVQAKHLGCVGLSCFYLAVKSIEEERNVPLATDLIRISQYRFTVSDLMRMEKIVLEKVCWKVKATTAFQFLQLYYSLIRETLPFERRNDLNFERLEAQLKACHCRIIFSKAKPSVLALAIIALEIQALKYVELTEGVECIQKHSKISGRDLTFWQELVSKCLTEYSSNKCSKPNGQKLKWIVSGRTARQLKHSYYRITHLPTIPETMG

>P46962|CTK2_YEAST CTD kinase subunit beta - Saccharomyces cerevisiae (Baker's yeast).

MPSTFESQLFFSRPFLSKRQIQRAQKNTISDYRNYNQKKLAVFKFLSDLCVQLKFPRKTLETAVYFYQRYHLFNRFETEVCYTVATSCLTLGCKEVETIKKTNDICTLSLRLRNVVKINTDILENFKKRVFQIELRILESCSFDYRVNNYVHIDEYVIKIGRELSFDYKLCNLAWVIAYDALKLETILVIPQHSIALAILKIAYELLDNKNWSSKRYSLFETDEKSVNEAYFDIVNFYINSFDMCDLQRHLPADLLPIGVERFMELKKNAGPESGLPQIPDHLLNADPYITITRDNNVQERRYVLSLELINGESSINSSTRHA

>P47821|SSN8_YEAST RNA polymerase II holoenzyme cyclin-like subunit - Saccharomyces cerevisiae (Baker's yeast).

MSGSFWTSTQRHHWQYTKASLAKERQKLWLLECQLFPQGLNIVMDSKQNGIEQSITKNIPITHRDLHYDKDYNLRIYCYFLIMKLGRRLNIRQYALATAHIYLSRFLIKASVREINLYMLVTTCVYLACKVEECPQYIRTLVSEARTLWPEFIPPDPTKVTEFEFYLLEELESYLIVHHPYQSLKQIVQVLKQPPFQITLSSDDLQNCWSLINDSYINDVHLLYPPHIIAVACLFITISIHGKPTKGSSLASAASEAIRDPKNSSSPVQIAFNRFMAESLVDLEEVMDTIQEQITLYDHWDKYHEQWIKFLLHTLYLRPASAI

>P51945|CCNG1_MOUSE Cyclin-G1 - Mus musculus (Mouse).

MIEVLTTDSQKLLHQLNTLLEQESRCQPKVCGLKLIESAHDNGLRMTARLRDFEVKDLLSLTQFFGFDTETFSLAVNLLDRFLSKMKVQAKHLGCVGLSCFYLAVKATEEERNVPLATDLIRISQYRFTVSDLMRMEKIVLEKVCWKVKATTAFQFLQLYYSLVHDTLPFERRNDLNFERLEAQLKACHCRIIFSKAKPSVLALSILALEIQALKYVELTEGVECIQKHSKISGRDLTFWQELVSKCLTEYSSNKCSKPNGQKLKWIVSGRTARQLKHSYYRITHLPTIPETIC

>P51946|CCNH_HUMAN Cyclin-H - Homo sapiens (Human).

MYHNSSQKRHWTFSSEEQLARLRADANRKFRCKAVANGKVLPNDPVFLEPHEEMTLCKYYEKRLLEFCSVFKPAMPRSVVGTACMYFKRFYLNNSVMEYHPRIIMLTCAFLACKVDEFNVSSPQFVGNLRESPLGQEKALEQILEYELLLIQQLNFHLIVHNPYRPFEGFLIDLKTRYPILENPEILRKTADDFLNRIALTDAYLLYTPSQIALTAILSSASRAGITMESYLSESLMLKENRTCLSQLLDIMKSMRNLVKKYEPPRSEEVAVLKQKLERCHSAELALNVITKKRKGYEDDDYVSKKSKHEEEEWTDDDLVESL

>P51947|CCNH_XENLA Cyclin-H - Xenopus laevis (African clawed frog).

MYHNSTQKKHWTFLSEDEPLRRRIQANVRYRARIRATEKPRLSEIFSLEPHEELAICKYYEKRLLDFCNAFKPTMPKSVLGTACMYFKRFYLNNSVMEHHPRIIMLTCVFLACKVDEFNVSSIQFVGNLGENPLGQEKILEQILEYELLLIQQLNFHLIVHNPYRPFEGFLIDVKTRYPMLENPEVLRKSADEFLNRVALTDACLLFAPSVIALTAILSTASRAGLNMESYLTECLSLKDNQETMSHLLDGMRRLKILVSKYEPARPEEVAALKKRLDHCHSTEVTLSVHGRKRKGYEDDGYISKKPKTEEDEWTDEDFGDSL

>P51959|CCNG1_HUMAN Cyclin-G1 - Homo sapiens (Human).

MIEVLTTTDSQKLLHQLNALLEQESRCQPKVCGLRLIESAHDNGLRMTARLRDFEVKDLLSLTQFFGFDTETFSLAVNLLDRFLSKMKVQPKHLGCVGLSCFYLAVKSIEEERNVPLATDLIRISQYRFTVSDLMRMEKIVLEKVCWKVKATTAFQFLQLYYSLLQENLPLERRNSINFERLEAQLKACHCRIIFSKAKPSVLALSIIALEIQAQKCVELTEGIECLQKHSKINGRDLTFWQELVSKCLTEYSSNKCSKPNVQKLKWIVSGRTARQLKHSYYRITHLPTIPEMVP

>P55168|CCNC_CHICK Cyclin-C - Gallus gallus (Chicken).

MAGNFWQSSHYLQWILDKQDLLKERQKDLKFLSEEEYWKLQIFFTNVIQALGEHLKLRQQVIATATVYFKRFYARYSLKSIDPVLMAPTCVFLASKVEEFGVVSNTRLISAATSVLKTRFSYAFPKEFPYRMNHILECEFYLLELMDCCLIVYHPYRPLLQYVQDMGQEDMLLPLAWRIVNDTYRTDLCLLYPPFMIALACLHVACVVQQKDARQWFAELSVDMEKILEIIRVILKLYEQWKNFDERKEMATILSKMPKPKPPPNSEGEQGPNGSQNSSYSQS

>Q10654|CCNB3_CAEEL G2/mitotic-specific cyclin-B3 - Caenorhabditis elegans.

MLRSQAKNVDLTSQADSRHQQKRKQAEQLDALKNPSEPAAKKQHSKGLTELRAHISGFKIDSAKRDPLGKSRTSRRDVENLPPQKSRYVDPCPHYDYDLEEAGNPDSISDYAQGIFDYYRHREVHFRVRKYLHKHPEVDVKTRAILIDWMVEIQETFELNHETLYNAVKLTDMYLCKTKNVDKNTIQKLACVAIFIAAKYDERSPPLVDDLIYLSGDRFSRDELLAMERELFATVGYDLGSPLSYRYLRRFGRVCRVDMKTLTMGRFILETSLMVYEYAMVSQSRLAAAAFVLAMRMLDKNNEYEWNPVLEKYSGFTGEEVMPLVEHMNHILHFSKDKWAQLTSVRQKYSHEVFFHVASIPMLPDTLKVVDSHTYAPVPMLSYP

>Q14094|CCNI_HUMAN Cyclin-I - Homo sapiens (Human).

MKFPGPLENQRLSFLLEKAITREAQMWKVNVRKMPSNQNVSPSQRDEVIQWLAKLKYQFNLYPETFALASSLLDRFLATVKAHPKYLSCIAISCFFLAAKTVEEDERIPVLKVLARDSFCGCSSSEILRMERIILDKLNWDLHTATPLDFLHIFHAIAVSTRPQLLFSLPKLSPSQHLAVLTKQLLHCMACNQLLQFRGSMLALAMVSLEMEKLIPDWLSLTIELLQKAQMDSSQLIHCRELVAHHLSTLQSSLPLNSVYVYRPLKHTLVTCDKGVFRLHPSSVPGPDFSKDNSKPEVPVRGTAAFYHHLPAASGCKQTSTKRKVEEMEVDDFYDGIKRLYNEDNVSENVGSVCGTDLSRQEGHASPCPPLQPVSVM

>Q52KE7|CCNL1_MOUSE Cyclin-L1 - Mus musculus (Mouse).

MASGPHPTSTAAAAAAAAASASSAAPSAGGSSSGTTTTTTTTTGGILIGDRLYSEVSLTIDHSLIPEERLSPTPSMQDGLDLPSETDLRILGCELIQAAGILLRLPQVAMATGQVLFHRFFYSKSFVKHSFEIVAMACINLASKIEEAPRRIRDVINVFHHLRQLRGKRTPSPLILDQNYINTKNQVIKAERRVLKELGFCVHVKHPHKIIVMYLQVLECERNQTLVQTAWNYMNDSLRTNVFVRFQPETIACACIYLAARALQIPLPTRPHWFLLFGTTEEEIQEICIETLRLYTRKKPNYELLEKEVEKRKVALQEAKLKAKGLNLDGTPALSTLGGFSPASKPSSPREVKAEEKSPVSINVKTVKKEPEDRQQASKSPYNGVRKDSKRSRTSRSASRSRSRTRSRSRSHSPRRHYNNRRSRSGTYSSRSRSRSRSHSESPRRHHNHGSPHLKAKHTREDLKSSNRHGHKRKKSRSRSQSKTRDHSDVTKKHRHERGHHRDRRERSRSFERSHKGKHHGGSRSGHGRHRR

>Q5BKF8|CCNL2_XENTR Cyclin-L2 - Xenopus tropicalis (Western clawed frog) (Silurana tropicalis).

MAANSSAVSSDGILIGDKLYSGVMISLENCLMAEERCALTPSVVDGIDVNTEIDLRCVGCELVQAAGILLRLPQVAMATGQVLFQRFFYTKSFVKHSMEHVAMACVHLASKIEEAPRRIRDVINVFHRLRQLREKQKSTPLILDQEYVNLKNQIIKAERRVLKELGFCVHVKHPHKIIVMYLQVLECERNKHLVQTSWNYMNDSLRTDVFVRFNPETIACACIFLAARTLEIPLPNRPHWFYLFGASEEDIKEICLQILRLYTRKKADVALLENKVEKRKLFIEEAKAKAKGLLPDGTPRLENAPEFSPSLKNDSPKELKANKPSPLAVHALKNCKRKVDGTKRPTSSSPVNGRVSKGRDSRSGSRSRDQSYSRSQSRSQSPKRRKSQSYSPSSDSKSRSPSRSRSDSPPHKPNHGSYKSTKGHVYGNNSDYKYQGHKRRSRSRSSSPSHSRSRESSDSGKYKKKDHYYRRERSRSYDRVSHRGYDREYHGHSHHRR

>Q5I0H5|CCNL2_RAT Cyclin-L2 - Rattus norvegicus (Rat).

MAAAAAGASGLMAPALAACSSGSGGAAPGSQGVLIGDRLYSGVLITLENCLLPDDKLRFTPSMSSGLDIDTETGLRVVGCELIQAAGILLRLPQVAMATGQVLFQRFFYTKSFVKHSMEHVSMACVHLASKIEEAPRRIRDVINVFHRLRHLREKKKPVPLVLDQEYVNLKNQIIKAERRVLKELGFCVHVKHPHKIIVMYLQVLECERNQHLVQTAWNYMNDSLRTDVFVRFQPESIACACIYLAARTLEIPLPNRPHWFLLFGATEEEIQEICFKILQLYTRKKVDLTHLESEVEKRKHAIEEAKARAKGLLPPGSAPGLDSATAGFSPAPKPESPKEGKGSKSSPLSVKNAKRKMEGPKKAKGDSPVNGLLKGQESRSQSRSREQSYSRSPSRSASPKRRKSDSGSTSGGSKSQSRSRSRSDSPPRQVHRGAPYKGSEVRGSRKSKDCKHLTQKPHKSRSRSSSRSRSRSRERTDSSGKYKKKSHYYRDQRRERSRSYERTGHRYERDHPGHSRHRR

>Q5R5D0|CCNG1_PONPY Cyclin-G1 - Pongo pygmaeus (Orangutan).

MIEVLTTTDSQKLLHQLNALLEQESRCQPKVCGLRLIESAHDNGLRMTARLRDFEVKDLLSLTQFFGFDTETFSLAVNLLDRFLSKMKVQPKHLGCVGLSCFYLAVKSIEEERNVPLATDLIRISQYRFTVSDLMRMEKIVLEKVCWKVKATTAFQFLQLYYSLLQENLPLERRNNINFERLEAQLKACHCRIIFSKAKPSVLALSIIALEIQAQKCVELTEGIECLQKLSKINGRDLTFWQELVSKCLTEYSSNKCSKPNVQKLKWIVSGRTARQLKHSYYRIAHLPTIPEMVP

>Q5SCB5|CCND_OSTTA Cyclin-D - Ostreococcus tauri.

MNRSRTSSFSTDRSASTVALAPDGDYERERVSALASWPEYAVDALRSEQDFIVHQSAALWTHRTPALHDPDSQETQLDVAKGLLARERETHGSFVFDARAAHHCAFRSQLVEWILDVCAGERFGPTTADVAIAYTDRVLSKTVVPKTSLHLVALCCLHIAVKYEEIEERVPTMSKLRSWTSNMYSPEIIRKMELAVLIELGWDLGVLTPAHFLESFLALTNGGISDGDDIEHGDAYKERYREELRYFVCQLYSLCVQDTSLLNQPPSQIASAVIATARVHLGVKPMCSPELRAAGNVTPQQIYPLVAHMLKLWDEACAEDEAMDEVETSAEFNSLTIQVPKPIGHDIVSKMGYEHRVTENASPTCPFDMQWDEE

>Q61458|CCNH_MOUSE Cyclin-H - Mus musculus (Mouse).

MYHSSSQKRHWTFASEEQLARLRADANRKFKCKAVANGKVLPNDPVFLEPHEELTLCKYYEKRLLEFCSVFKPAMPRSVVGTACMYFKRFYLNNSVMEYHPRIIMLTCAFLACKVDEFNVSSPQFVGNLRESPLGQERALEQILEYELLLIQQLNFHLIVHNPYRPFEGFLIDIKTRYPMLENPEILRKTADDFLSRIALTDAYLLYTPSQIALTAILSSASRAGITMESYLSESLMLKENRTCLSQLLDIMKSMRNLVKKYEPPRSDEVAVLKQKLERCHSSDLALNAVTKKRKGYEDDDYVSKKPKQEEEEWTDDDLVDSL

>Q62447|CCNC_MOUSE Cyclin-C - Mus musculus (Mouse).

MVAPRPLCWLVHLYQGKQYYSMAGNFWQSSHYLQWILDKQDLLKERQKDLKFLSEEEYWKLQIFFTNVIQALGEHLKLRQQVIATATVYFKRFDARYSLKSIDPVLMAPTCVFLASKVEEFGVVSNTRLIAATTSVLKTRFSYAFPKEFPYRMNHILECEFYLLELMDCCLIVYHPYRPLLQYVQDMGQEDVLLPLAWRIVNDTYRTDLCLLYPPFMIALACLHVACVVQQKDARQWFAELSVDMEKILEIIRVILKLYEQWKNFDERKEMATILSKMPKPKPPPNSEGEQGPNGSQNSSYSQS

>Q6GN15|CCNL1_XENLA Cyclin-L1 - Xenopus laevis (African clawed frog).

MAAVPQLSAPSAPARSADGILIGDRQYSEVYLTIDYSLIPEERLSPTPSMSDGLDLNTETDLRILGCELIQSAGILLRLPQVAMATGQVLFHRFFYSKSFVKHSFEIIAMACINLASKIEEAPRRIRDVINVCHHLRQIRAKRTPSPLILDQSYINTKNHVIKAERRILKELGFCVHVKHPHKIIVMYLQVLECERNQTLVQTAWNYMNDCLRTNVFVRFDAETIACACIYLAARALQLSLPNRPHWFLLFGATEENIQDICITTLRLYSRIKPNYEFLEKEVDKRKVALQEAKLKAKGLNPDGTPAILSMGGFSPASKPSSPRDVKTEEKSPNFAKVKREMDDKQSSKSPYNGLRKENKRSRSVSRSRSRTKSRSRSHSPRRHYNNRRRSRSGTYSSRSRSRSRSHSESPRRHHNHGSPHMKLKHRVEDLRGRHAHKRKKSHSPSKSREPSELAKKHRHEHGHHRERRERSRSFERSHKNKHHGSSHSGHGRHRR

>Q7ZVX0|CCNL1_BRARE Cyclin-L1 - Brachydanio rerio (Zebrafish) (Danio rerio).

MSLGMLSPHLNTPPPNNQGILIGDKVYSEVFLAIDNSIIPEDRLSTTPSMLDGLDHETETDLRILGCERIQSAGILLRLPQVAMATGQVIFQRFFFSKSFVKHNFEIVAMACVNLASKIEESPRRVRDVINVFHHLKQGKGKKSTPLILDQNYINTKNQVIKAERRILKELGFCVHVKHPHKIIVMYLQVLECEKNQMLVQTAWNYMNDALRTSAFVRFEPETIACACIYLAARVLQIPLPSKPHWFLLFGATKEDIKEICINTMKLYSREKPHSEQLERQVEKRKIFLEEARLKARGQNPNGTPALASINGFSPASKPSSPRDVKMDDKSPNSKLKEPENRQLFAKSPLNGSIKKEDGKVFQNGKNHSRSRSRSTSRSPHRHRRSHSGTYSSHSSHSPSPRQKARRPSPISQLRTDRDRPSETSRHSNKRRRSRSRSRSNSRERVRDRDHIKHKQERSGSGHHWDHRDRERDRSRDHGRNKRQSRSHSGHSHSRHRR

>Q8BGU5|CFP1_MOUSE Cyclin fold protein 1 - Mus musculus (Mouse).

MGNTTSCCVSSSPKLRRNAHSRLESYRPDTDLSREDTGCNLQHISDRENIDDLNMEFNPSDHPRASTIFLSKSQTDVREKRKSLFINHHPPGQTSRKYSSCSTIFLDDSTVSQPNLKYTIKCVALAIYYHIKNRDPDGRMLLDIFDENLHPLSKSEVPPDYDKHNPEQKQIYRFVRTLFSAAQLTAECAIVTLVYLERLLTYAEIDICPANWKRIVLGAILLASKVWDDQAVWNVDYCQILKDITVEDMNELERQFLELLQFNINVPSSVYAKYYFDLRSLAEANNLSFPLEPLSRERAHKLEAISRLCEDKYKDLRKPMRKRSASADNLILPRWSPAIIS

>Q8ND76|CFP1_HUMAN Cyclin fold protein 1 - Homo sapiens (Human).

MGNTTSCCVSSSPKLRRNAHSRLESYRPDTDLSREDTGCNLQHISDRENIDDLNMEFNPSDHPRASTIFLSKSQTDVREKRKSLFINHHPPGQIARKYSSCSTIFLDDSTVSQPNLKYTIKCVALAIYYHIKNRDPDGRMLLDIFDENLHPLSKSEVPPDYDKHNPEQKQIYRFVRTLFSAAQLTAECAIVTLVYLERLLTYAEIDICPANWKRIVLGAILLASKVWDDQAVWNVDYCQILKDITVEDMNELERQFLELLQFNINVPSSVYAKYYFDLRSLAEANNLSFPLEPLSRERAHKLEAISRLCEDKYKDLRRSARKRSASADNLTLPRWSPAIIS

>Q96S94|CCNL2_HUMAN Cyclin-L2 - Homo sapiens (Human).

MAAAAAAAGAAGSAAPAAAAGAPGSGGAPSGSQGVLIGDRLYSGVLITLENCLLPDDKLRFTPSMSSGLDTDTETDLRVVGCELIQAAGILLRLPQVAMATGQVLFQRFFYTKSFVKHSMEHVSMACVHLASKIEEAPRRIRDVINVFHRLRQLRDKKKPVPLLLDQDYVNLKNQIIKAERRVLKELGFCVHVKHPHKIIVMYLQVLECERNQHLVQTSWNYMNDSLRTDVFVRFQPESIACACIYLAARTLEIPLPNRPHWFLLFGATEEEIQEICLKILQLYARKKVDLTHLEGEVEKRKHAIEEAKAQARGLLPGGTQVLDGTSGFSPAPKLVESPKEGKGSKPSPLSVKNTKRRLEGAKKAKADSPVNGLPKGRESRSRSRSREQSYSRSPSRSASPKRRKSDSGSTSGGSKSQSRSRSRSDSPPRQAPRSAPYKGSEIRGSRKSKDCKYPQKPHKSRSRSSSRSRSRSRERADNPGKYKKKSHYYRDQRRERSRSYERTGRRYERDHPGHSRHRR

>Q9JJA7|CCNL2_MOUSE Cyclin-L2 - Mus musculus (Mouse).

MAAAAAGAAGLMAPASAACSSGSAGAAPGSQGVLIGDRLYSGVLITLENCLLPDDKLRFTPSMSSGLDVDTETGLRVVGCELIQAAGILLRLPQVAMATGQVLFQRFFYTKSFVKHSMEHVSMACVHLASKIEEAPRRIRDVINVFHRLRHLREKKKPVPLVLDQEYVNLKNQIIKAERRVLKELGFCVHVKHPHKIIVMYLQVLECERNQHLVQTAWNYMNDSLRTDVFVRFQPESIACACIYLAARTLEIPLPNRPHWFLLFGATEEEIQEICFKILQLYTRKKVDLTHLESEVEKRKHAIEEAKARAKGLLPGTAPGLDSAAGFSPAPKLESPKEGKGGKPSPPSGKSAKRKMEGPKKAQGHSPVNGLLKGQESRSQSRSREQSYSRSPSRSASPKRRKSDSGSTSGGSKSQSRSRSRSDSPPRQVHRGAPYKGSEVRGSRKSKDCKYLTQKPHKSRSRSSSRSRSRSRERTDNSGKYKKKSHYYRDQRRERSRSYERTGHRYERDHPGHSRHRR

>Q9QWV9|CCNT1_MOUSE Cyclin-T1 - Mus musculus (Mouse).

MEGERKNNNKRWYFTREQLENSPSRRFGVDSDKELSYRQQAANLLQDVGQRLNVSQLTINTAIVYMHRFYMIQSFTQFHRYSMAPAALFLAAKVEEQPKKLEHVIKVAHTCLHPQESLPDTRSEAYLQQVQDLVILESIILQTLGFELTIDHPHTHVVKCTQLVRASKDLAQTSYFMATNSLHLTTFSLQYTPPVVACVCIHLACKWSNWEIPVSTDGKHWWEYVDATVTLELLDELTHEFLQILEKTPSRLKRIRNWRAYQAAMKTKPDDRGADENTSEQTILNMISQTSSDTTIAGLMSMSTASTSAVPSLPSSEESSSSLTSVDMLQGERWLSSQPPFKLEAAQGHRTSESLALIGVDHSLQQDGSSAFGSQKQASKSVPSAKVSLKEYRAKHAEELAAQKRQLENMEANVKSQYAYAAQNLLSHDSHSSVILKMPIESSENPERPFLDKADKSALKMRLPVASGDKAVSSKPEEIKMRIKVHSAGDKHNSIEDSVTKSREHKEKQRTHPSNHHHHHNHHSHRHSHLQLPAGPVSKRPSDPKHSSQTSTLAHKTYSLSSTLSSSSSTRKRGPPEETGAAVFDHPAKIAKSTKSSLNFPFPPLPTMTQLPGHSSDTSGLPFSQPSCKTRVPHMKLDKGPPGANGHNATQSIDYQDTVNMLHSLLSAQGVQPTQAPAFEFVHSYGEYMNPRAGAISSRSGTTDKPRPPPLPSEPPPPLPPLPK

>Q9R1Q2|CCNL1_RAT Cyclin-L1 - Rattus norvegicus (Rat).

MASGPHPTSTAAAASASSAAPSAGGSSSGTTTTTTTTTGGILIGDRLYSEVSLTIDHSVIPEERLSPTPSMQDGLDLPSETDLRILGCELIQAAGILLRLPQVAMATGQVLFHRFFYSKSFVKHSFEIVAMACINLASKIEEAPRRIRDVINVFHHLRQLRGKRTPSPLILDQNYINTKNQVIKAERRVLKELGFCVHVKHPHKIIVMYLQVLECERNQTLVQTAWNYMNDSLRTNVFVRFQPETIACACIYLAARALQIPLPTRPHWFLLFGTTEEEIQEICIETLRLYTRKKPNYELLEKEVEKRKVALQEAKLKAKGLNLDGTPALSTLGGFSPASKPSSPREVKAEEKSPVSINVKTVKKEPEDRQQASKSPYNGVRKDSKRSRNSRSASRSRSRTRSRSRSHTPRRHYNNRRSRSGTYSSRSRSRSRSHSESPRRHHNHGSPHLKAKHTREDLKSSNRHGHKRKKSRSRSQSKTRDHSDVTKKHRHERGHHRDRRERSRSFERSHKGKHHGGSRSGHGRHRR

>Q9UK58|CCNL1_HUMAN Cyclin-L1 - Homo sapiens (Human).

MASGPHSTATAAAAASSAAPSAGGSSSGTTTTTTTTTGGILIGDRLYSEVSLTIDHSLIPEERLSPTPSMQDGLDLPSETDLRILGCELIQAAGILLRLPQVAMATGQVLFHRFFYSKSFVKHSFEIVAMACINLASKIEEAPRRIRDVINVFHHLRQLRGKRTPSPLILDQNYINTKNQVIKAERRVLKELGFCVHVKHPHKIIVMYLQVLECERNQTLVQTAWNYMNDSLRTNVFVRFQPETIACACIYLAARALQIPLPTRPHWFLLFGTTEEEIQEICIETLRLYTRKKPNYELLEKEVEKRKVALQEAKLKAKGLNPDGTPALSTLGGFSPASKPSSPREVKAEEKSPISINVKTVKKEPEDRQQASKSPYNGVRKDSKRSRNSRSASRSRSRTRSRSRSHTPRRHYNNRRSRSGTYSSRSRSRSRSHSESPRRHHNHGSPHLKAKHTRDDLKSSNRHGHKRKKSRSRSQSKSRDHSDAAKKHRHERGHHRDRRERSRSFERSHKSKHHGGSRSGHGRHRR

>Q9XT26|CCNT1_HORSE Cyclin-T1 - Equus caballus (Horse).

MEGERKNNNKRWYFTREQLENSPSRRFGLDPDKELSYRQQAANLLQDMGQRLNVSQLTINTAIVYMHRFYMIQSFTQFHRNSVAPAALFLAAKVEEQPKKLEHVIKVAHACLHPQESLPDTRSEAYLQQVQDLVILESIILQTLGFELTIDHPHTHVVKCTQLVRASKDLAQTSYFMATNSLHLTTFSLQYTPPVVACVCIHLACKWSNWEIPVSTDGKHWWEYVDATVTLELLDELTHEFLQILEKTPNRLKRIRNWRACQAAKKTKADDRGTDENTSEQTILNMISQSSSDTTIAGLMSMSTSSTTSTVPSLPTTEESSSNLSGVEMLQGERWLSSQPPFKLEPAQGHRTSENLALIGVDHSLQQDGSNAFISQKQNSSKSVPSAKVSLKEYRAKHAEELAAQKRQLENMEANVKSQYAYAAQNLLSHHDSHSSVILKMPIEGSENPERPFLEKPDKTALKMRIPVASGDKAASSKPEEIKMRIKVHAAPDKHNSIDDSVTKSREHKEKHKTHPSNHHHHHNHHSHKHSHSQLPAGTGNKRPGDPKHSSQTSTLAHKTYSLSSSFSSSSSSRKRGPPEETGGALFDHPAKIAKSTKSSSINFFPPLPTMAQLPGHSSDTSGLPFSQPSCKTRVPHMKLDKGPTGANGHNTTQTIDYQDTVNMLHSLLHAQGVQPTQPPALEFVHSYGEYLNPRAGGMPSRSGNTDKPRLPPLPSEPPPPLPPLPK

>Q9Z2V9|CCNI_MOUSE Cyclin-I - Mus musculus (Mouse).

MKFPGPLENQRLSSLLERAISREAQMWKVNVPKIPTNQNVSPSQRDEVIQWLAKLKYQFNLYPETFALASSLLDRFLATVKAHPKYLNCIAISCFFLAAKTVEEDEKIPVLKVLARDSFCGCSSSEILRMERIILDKLNWDLHTATPLDFFHIFHAIAVSTRPQLLFSLPKLSPSQHLAVLTKQLLHCMACNQLLQFKGSMLALAMVSLEMEKLIPDWLPLTIELLQKAQMDSSQLIHCRELVAYHLSALQSALPLNSVYVYRPLKHTLVTCDKGAFKLHPSSVSGPDFSKDNSKPEVPVRGPAAFHLHLPAASGCKQTSAKRKVEEMEVDDFYDGIKRLYNEDNGPENVGSVCGTDLSRQEGHASPCPPLQPVSVM

>O02115|PCNA_CAEEL Proliferating cell nuclear antigen - Caenorhabditis elegans.

MSLQAMDSSHVALVSLKLEVGLFDTYRCDRTINLGLSLANMSKALKCANNDDTCMLKYEENEGDSIIFTFADPKRDKTQDVTVKMMDIDSEHLGIPDQDYAVVCEMPAGEFQKTCKDLSTFSDSLNITATKAGIVFTGKGDIGSSVVTYSPSSNTDDETEAVTLEVKDPVNVNFSIKYMNQFTKATALSDRVRLSLCNDVPVVVEYPIEENGYLRFYLAPKIDDDENMD

>P57763|PCNA2_SULOH DNA polymerase sliding clamp B - Sulfurisphaera ohwakuensis.

MIKATYSSAKDFYSLLSGLLRVTDEIILNFTEDSIFSRYLTDDKVLMVIFKIPKEYLEDYTIDKPLGIKININDLKKILGKAKTKSATVTLEETEAGLKVTVRDEKTGTRSNIYIKGEKTSIDQLTEPKVNLSVTFTTDGDILKDIARDLSLVGEEVEISADENTVTLSTEEAGRTYKSLLRQDKPLKSLNIESPSKAVYSIEVLKDVFKVTTISQNVTVGFGNNIPMRIEVPTDSGGQLIFWIAPRL

>P57766|PCNA2_SULSO DNA polymerase sliding clamp B - Sulfolobus solfataricus.

MFKIVYPNAKDFFSFINSITNVTDSIILNFTEDGIFSRHLTEDKVLMAIMRIPKDVLSEYSIDSPTSVKLDVSSVKKILSKASSKKATIELTETDSGLKIIIRDEKSGAKSTIYIKAEKGQVEQLTEPKVNLAVNFTTDESVLNVIAADVTLVGEEMRISTEEDKIKIEAGEEGKRYVAFLMKDKPLKELSIDTSASSSYSAEMFKDAVKGLRGFSAPTMVSFGENLPMKIDVEAVSGGHMIFWIAPRL

>Q74MV1|PCNA_NANEQ DNA polymerase sliding clamp - Nanoarchaeum equitans.

MRVTFPDAKALKKIVPIVADLISEGQFVATEEGIKLVAMDPASIAMVIWEMKPEAFIDYTIEGDKEIITVSMDDLKTIVKKLKQREMVVWETDREKNKLKILARGTIKKTFSIPLLEGEETETPIPSLEYNNVVELDSKAIKEIIDDASAIADSLKFKAEPPSKLIIKAEGEMKEMTVELTEGEDAVVSIDIQEEAYASYSIDYLKKFAKAADVSDIAILKLKTDYPLWLEYRYLDKMTLIFILAPRSD

>Q7T6Y0|PCNA_MIMIV Probable DNA polymerase sliding clamp - Mimivirus.

MSKKTGTKTSKSGSKKSNKDVEKLVDDEDIQDLSDKKNKISQSKNLVKNNKSSKNSKSSKSVKSTKTSTKTSLKKQVKTPPKKQTKTSSKKNKKDESDDDVTDNSDISDDDNTGNSDISDDENDQEEDNDISSDEEETETRQKSSGKKGNVNKDVGSSRKLKTDKNARNDNKVLEIRTTQTGALKQVFERVSGVISDCCLTFMPADKDINNAGDDNEYYEDESTKSSHKQSKTTDRPKNTGGIRIIRLTEDNNTLVKVVLEAANFEYFRCDEPKITVGVDMHTLHSHLKMINDDDPIVIYMKKDIQGSLYIRSLSENNDNSEEREIELFLMDIINPEIPVPKTEFQNRITMKSDKFHLICKHLSQNSTFVEITSINNEILFKGQSEGGKVTMTYKDTGYKKKEKPDQVIQGVYELRNLLGFSKCNKLCNTIEIYLKNDFPLVLVISVATLGKMYVFLSPIDNGN

>Q8ZYL6|PCNA2_PYRAE DNA polymerase sliding clamp B - Pyrobaculum aerophilum.

MSVRALFPKGKEPRYAFEVLIRMLPEAVLNFSSDGISLKALDPTKTALLDLTFYATALEDYSIDEETKVGIIFTTIKDVIKRIGATEKLELEVDKERNRFSFYIYPKKGREVGLVRRFSFPIVQVLEEEIPELAVSFDASFEIDSAVLDDILAMVDEVSDWIQITVSPDKVLFRGVGEGGKAAETELSYDSESVFNISAGEAASAKYSVEMLRDISGKMKSLSKRVKVELSANKPIRLTYEFTSGVFTATIAPRVD

>Q975M2|PCNA2_SULTO DNA polymerase sliding clamp B - Sulfolobus tokodaii.

MIKATYSSAKDFYSLLSGLLKVTDEIILNFTEDSIFSRYLTDDKVLMVIFKIPKEYLEDYTIDKPLGIKININDLKKILGKAKSKSATVTLEETEAGLKVTVRDEKTGTRSNIYIKGEKTSIDQLTEPKVNLSVTFTTDGDVLKDIARDLSLVGEEVEISADENTVTLSTEEAGRTYKSLLKQDKPLKSLNVESPSKAVYSIEVLKDVFKVTSISQNVTVGFGNNIPMKIEVPTDSGGQLIFWIAPRL

>Q9YEZ5|PCNA3_AERPE DNA polymerase sliding clamp B2 - Aeropyrum pernix.

MADARFYFSDARTWRYMVASIEKIIEEGVFVATGEGLSLRALDTSHVAMVDLYYPNTAFIEYDIGGESVEFGVSFDLLSKVLRRARKEDELVLEVEGSRLAVKLKSRGERTFRIPQVVMTYEKLPEPKVSFTVRARMLGSTFREAVRDLEPHSETLTLRALEDALLLVGSSEMATVEIELSQSRGSLLDYEAESQDRASYSIEYFSEMLSAAQAADAVVVSFSEDAPVRVDMEYLGGGRLTFYVSPKIE
